# Supplementary material for: What are the positive drivers and potential barriers to implementation of hospital at home selected by low-risk DECAF score in the UK: a qualitative study embedded within a randomised controlled trial
Source: BMJ Open. 2019 Apr 4;9(4):e026609. doi: 10.1136/bmjopen-2018-026609 (PMC6500229; doi:10.1136/bmjopen-2018-026609)
Supplement: Supplementary data [file bmjopen-2018-026609supp001.pdf]

Table of themes with quotations

Online supplementary report of full and additional quotations related to themes. Interviewer quotes are shown in bold.

Theme 1. Positive Drivers for HAH	Quotes
1.1 Availability of home comforts and maintaining independence	I couldn't even walk up the ward or anything you know because I only had this like little lead you know and I used to keep forgotten walking off then if you wanted to talk to anybody you had to either shout or they had to come to you, you know but that's the only thing just sort of sat there all the time (P22L145-148)<br><br>**And now in the home how would you describe this situation with the oxygen lead?**<br><br>Oh yes I can go upstairs do different pieces up there you know tidy my draws up and that's you know just a couple at a time just err no its nice and I can get up and make myself a meal you know (P22L145-153)<br><br>Yeah well what can you do in hospital you're either lying on a bed or sitting in a chair I can walk back and forward in here you know the and I can go upstairs and down bath wash shave that type of thing you know were as well you can do that in hospital get washed and shaved and that but you're stuck in the one place you're not going anywhere you know you're not getting no exercise nothing (P18L114-118)<br><br>In a lot of ways you're disempowering the patient by keeping them on a ward and keeping them within that sort of very tight sort of role, when you get them home then they're, they're dressed, they are doing more things themselves (N7L293-296)
1.1.1 Perception of a quicker recovery during HAH and positive effects on perceived breathlessness	Her recovery time was much quicker so it was easier for me because she was helping herself do you know what I mean (CP28L260-261)<br><br>Just so relaxed content, content with the care everything just made everything because when your anxious obviously it reacts on your breathing and your whole persona really and just everything was so relaxed I don't know how else to describe it (P6L109-112)
1.1.2 Improved sleep and nutrition	People recover better in their own home and there was one lady and I quote her often to the staff there's no bed like your own bed and that stuck because actually she was right absolutely right you know why be sitting in

	a hospital bed when you've got poorly people around you if you have that support to be able to recuperate at home (M19L128-132).<br><br>I was very surprised at the food it wasn't too bad. In fact I had more in the hospital than I did at home (P10L92-97)<br><br>You don't eat the same in hospital for a start and you certainly don't sleep the same you can be awake most of the night (P6L91-92)
1.1.3 More convenient place to be with friends and family	It's easier for my family to visit and everything you know getting cars parked you know when they have been to work all day by the time they get the hospital it's time to come home you know so it's definitely benefitted me you know (P22 L21-24)<br><br>But even the grand children when I'm in hospital I don't see the grandchildren I've got six grandchildren and three of them live next door and they're here every single day when I'm not here they do cry they get really upset (P13 L167-169)<br><br>They don't like fetching them in when they're so young I mean the youngest ones four and the twins and they miss us when I'm not here (P16L87-88)<br><br>It's better my husband being at home and getting the treatment at home instead of like going back and forward to the hospitals and especially with being a carer looking after two children as well you know it's a lot better<br><br>**So when you say you look after two children as well, so are you a carer for those two children and Jim?**<br><br>Yes<br><br>**So hospital at home how does that make it easier in regards to that?**<br><br>Like with looking after Jim and getting the support and like for visit and things you know like when he's at home he's at home and getting looked after at home and I can think of the children and it doesn't put as much

	pressure on us as well because like when you're visiting and you've got children to pick up at school as well you know it's all like the time as well (P23 L537-548)<br><br>It's been much better for me anyway because I used to get knackered go into hospital every day and coming back then having to go back again and coming back whereas been here I can go to work come in do whatever I wanted to do when I knew she was safe she was all right in the house do you know what I mean (P28L225-228)<br><br>Financial yes, yes because you see were on a low income my partner only works part time window cleaner and we have to pay full rent and everything and money is really tight and for him to keep coming back and forth to hospital I mean sometime if there's no money he can't go you know so that's the benefits because he even though he's a part time window cleaner he can still go to work when I'm poorly he works local (P13L56-61)<br><br>At first I was a bit concerned because I look after the grandchildren not every day four days a week sometimes erm and I was more concerned about them whether it would interfere with personal life sort of thing err and then went and thought about it, it obviously wouldn't I'd get to see them more than what I would actually been in hospital so that was the deciding factor really (P6L12-16)
1.2 Confidence in the continuity of HAH care<br>1.2.1 Feelings of safety, reassurance and appreciation	I had confidence in the team that came out I think I saw four different people over a short period of time they all seemed to me to be very well trained and putting me at ease and I really didn't feel like I was missing anything that I would have had in hospital you know (P1L70-73)<br><br>Now I feel what's the word more secure I don't know yeah more secure because I know all I've got to do is make a phone call like obviously there's name of doctor there's obviously the nurses if I have to there would be you there is more people to talk to now and that's something I never had before which is good' (P13L103-106)<br><br>Few have said but are you not nervous X like if you're in a hospital you're on a ward they get a doctor to you as soon as they can and they might not get it as soon as you can might be two three hours down the line but if your hospital at home if you take really poorly yes you phone up you've got that number they will still be here as quick as they would if you were in a hospital do you know what I mean it doesn't mean to say because you're on a ward that a nurse or a doctors going to get to you do you know and I would phone a the district nurse or community nurse and if they thought you needed a doctor or the hospital they would phone an ambulance straight away (P12L321-328)

	Telephone call before I came out I got a telephone call before the afternoon in the evening check that things were okay I was told on that evening who would be coming the next day so I knew who to expect so I don't have a problem at all with it (P1 P7 L320-323)
1.2.2 Personalised relation and specialist expertise of the RSN	A wash down behind the curtain I was frightening I was rushing (P32L53-55)<br><br>I mean you cannot get individual care can you really because there's 16 on the ward another five people in care and of course at home you're getting the care more attention (P16 L163-165)<br><br>They checked everything every day you know everything ankles everything whereas a rule it's just your blood pressure and your oxygen level in the hospital and your erm temperature yes but I don't know they were just really brilliant they were here quite a long time you know making sure I was feeling all right and I was confident on my own you know but no I found them really good and there are so nice you know really nice and caring (P22L44-49)<br><br>I'll be perfectly honest the times I've been in the hospital the nurses there been too busy or there's only been one on or two on and they can't do anything you know there to busy doing other things you don't get a one to one erm but in your home you do get one to one (P12L208-211)<br><br>She made me feel safe she made me feel I was getting a proper treatment erm comfortable is the word she made me feel really comfortable knew what she was doing explained everything even down to when you were getting your stats done you know when you were getting while you were getting your bloods taken erm explained everything you don't always get that in a hospital (P12L365-370)<br><br>You get told you can get out tomorrow and then they say you've got tablets and everything and by the time its tea time you're still waiting there somebody coming to get you or getting the tablets or getting the letter but everything's so well done there was didn't get you harassed at all and it just seemed like you go home today you've got your tablets you've got your letter you were taken down to the departure place the nurse was there they had been with the oxygen so well planned it was like err just sail a long and I like that because it didn't get as well erm and it made you feel more relaxed and thinking to yourself well if this is how it's going to be then that's okay I can cope (P20L423-431)
2. Potential Barriers and	

Negative Influences of HAH Care Pathway	
2.1 Fear of being alone at home	The hours I would be on my own you know if just say I got up to go the toilet and my legs went what do you do then if you can't breathe you know you've got no phone at hand you know you cannot pull an alarm switch or anything so no (Decliner 1 P2L39-41)<br><br>Maybe it's the generation thing I mean mention a social working oh no I haven't got one of them but I mean you know I think they think if there've got a social worker or they're involved with social services it's like there labelled but it's not that but they don't realise that so you know until its explained to them so there would be sat at home and they wouldn't realise they could just ring social services and ask for help (N4L100-104)<br><br>Some people maybe prefer to be in a cocoon of a hospital environment, they maybe worry if something goes wrong someone's seconds away from them (P1L226-229)
2.2 Privacy issues and not wanting visitors	I thought people coming to my house, I cannot I'm frightened that it wouldn't be tidy and things like that, that's what type of person I am I'm frightened that it wouldn't be tidy (D3L4-7)<br><br>My husband and I are divorcing and my house just having been sold the atmosphere at home wouldn't be conducive in recuperating…but in the future ... I would very much like to be involved (D9L14-21).
2.3 Resistance to change<br>2.3.1 Reluctance of the removal of nebulised therapy	The nebulisers seem to clear you and they clear you quickly and they seem as if erm you get relief after you've had them (P12L90-91)<br><br>I mean I take all my self-medication at home anyway but you know I'm on steroids and antibiotics and so I've got them at home anyway as I say it's just if I had a nebuliser at home that would sort of cure everything I think (P7L57-59)<br><br>I would still rather have home treatment you know if I had a nebuliser I would be happy as Larry I would be because once I'm on that and take a couple of doses I'm fine (P7L69-71)<br><br>I feel safer actually with that there (P27L311-313)

	I think a lot of them have the misconception that if they have a nebuliser they're better and that seems to stick in their mind so of course then we have to go down do you realise it increased your heart rate its higher mortality rate ideally it's for emergency you shouldn't be having them all the time so we do sort of stress to them you don't want to be on this treatment your inhalers are far better more practical more easier to use so we do try our best to get them off (N3L223-229)
2.3.2 Challenging clinicians' preconceptions, accepting a new model of care and operational concerns	The patient themselves may not be ready for it I may be ready for it but the patient may be two days away from it and also sometimes the patient needs would like if someone came into a hotel for their illness for a while they want to be away from their sick bed because their home is almost like a toxic area so all I'm doing is what I'm trying to tell the patient is I can actually bring the hospital to your home with some patients it's a good idea but some patients say actually I want to take a break from my home<br><br>**So is that the major difference in those patients who want to stay in hospital?**<br><br>Yeah because I think they need to stay in hospital where they feel safer not because it is safer but basically they feel safer and also it gives a break their carers would like to get rid of them for two or three days there's many different things going on in the patient the mind of the patient<br>(Co2 P2L53-66)<br><br>That's kind of often how people view patients with COPD that they want to come to hospital but actually it's not true (Co3 L198-201)<br><br>Our work in trying to get the centre to change the way erm we can record the data for hospital at home patients erm perhaps by us doing that it would make it easier for other trusts to go down the hospital home route in the future (M1P4 L151-153)<br><br>If we're employing consultants junior doctors respiratory nurses specialists nurses and health cares we're not having the patient in hospital so we're not getting the tariff for that but we're sending those patients out to look after them somebody has to pay the wage bill for that and it's about understanding that payment mechanism and if that's absolutely crystal clear then that becomes a lot easier for everybody because that's the kind of thing that will stop people moving forward (M17L188-193)

	Think the only concern I would have is that hospital at home is only as good as the nurses you've got on (P12L336-337)
2.4 Negative Influences of HAH	I mean for all I'm a smoker when I'm in hospital, things like that, I don't think about smoking whatsoever where I am at home I do but erm whenever I'm in a strange place like that I never smoking never crosses my mind (P14L273-276).<br><br>I've made some nice friends when I was in for a week you know still ring them you know erm but that was nice but I didn't wander round and talk to them you know but erm as I say erm the first time I went in it was nice (P22L497-499)<br><br>It's frightening for them to see when you're not well it's not nice for my husband to sit and watch us when I'm bad (P25L220-222)
3. Other Insights<br>3.1 Unintentional change in UC	There's a much more emphasis on getting people home early and I think even the people who are in the usual care arm so the people who we aren't doing in the hospital at home I think people are now more likely to send them home a lot earlier than they did when I did my stuff four years ago so I think general practice has changed we're not hanging on to these patients as long as we did erm and that's through external pressures erm but you know external pressures make you want to get people home as quickly as possible but the trial itself might be running the trial might also be changing peoples practice and may be making them send people home earlier (Co3 L121-129)
3.2 Early uncertainty with HAH selected by DECAF	They have certain reservations so you've got that conflict between there clinical impression and the predictive score. There are patients where as I said their risk is low but the clinical judgement is that they are not low risk now the difficulty here is that we shouldn't necessarily ignore our clinician judgement because well it's important and it's important to listen to however again and again the studies show that clinical judgement is not as good as prognostic scores so you've got research from the 1950s and literally hundreds of studies now from medicine and kind of baseball wine-making you name it completely different types of erm different types of expertise the simple score almost always out performs clinical judgement but clinicians don't like to think that's true (Co18L40-52).<br><br>For some cases it seems obviously a good thing to do then there's other cases where previously you might have

	thought better keep those inpatients in for a few days rather than sending them home so a couple of patients were that's realise that's different from what you normally do and you do think not anxiety but you do think oh I hope that things are going to alright but that's why we're doing a trial because we don't know (Co3L22-27)<br><br>As you see it works and as you get evidence suggesting it works and its safe you're more confident in doing it (Co6L154-155)<br><br>I think part of the main advantage from my point of view is making me more aware of the safety of the DECAF score and the ability that people can be treated out of the hospital so more whether that's because of the hospital at home trial or whether that's just because of the gradual change in practice because of the DECAF score I'm not sure (Co6L99-103)

Interview questions – Carers

Background to the carer

1) Can you describe your relationship to (X)?
2) Can you describe the care you give at home?
3) What sort of care do you provide?
4) Can you describe a typical day/how much of your day does it take?
5) How has your life changed since you have been providing care?
    - Has it affected your daily routine (what was it like before/how is it now – how would you describe what you do – how do you feel)?
    - What's good or bad about it?
    - Is it becoming easier/harder – in what ways?
    - What do you find hardest about caring for someone? (loneliness/fear/isolation/financial difficulties)
6) How has care changed over time?
7) Is there a change when your relative's health suddenly worsens?
8) What helps you in providing care?
    - (coping/support/faith/family)
    - Do you receive any help/support from anyone?
10) How do you feel about providing care? (burden/guilty/helpless)
11) What do you think about someone from a social services team coming in to help provide care?
12) What does the word carer mean to you/how do you feel about the word carer?

Project Questions

1) What are your perceptions of hospitals?
2) Has your relative been previously hospitalised?
3) Can you describe past experiences of hospital?
4) How do you feel if your relative has to go into hospital?
5) Can you describe a typical day on which your relative is hospitalised – what type of care do you provide?
6) Can you tell me what your initial thoughts were when you were told about HAH?
7) Initially your reasons for preferring home treatment were (QUOTE). Can you explain this in more detail?
8) How did you find (mode of treatment)?
    - Expectations of (mode of treatment)/different from your experience?
9) How was it being at (mode of treatment) with your relative?
10) How would you have felt if (X) was treated (mode of treatment)?
11) Can you describe what happened whilst (X) was being treated (mode of treatment)?
    - What did they do whilst at home? Relaxed or not?
12) What is your preference now following your recent experience?
13) If in the future HAH is available, would you choose to have your relative treated at home or in hospital? Why?

14) Can you describe the care you give your relative comparing when at home and hospitalised? – any differences?
15) Where would you prefer to care for your relative?
16) How does it benefit you?
17) Any disadvantages of having to care for your relative at (mode of treatment)?
18) What concerns would you have in the future if your relative is treated at (mode of treatment)?

Dr-patient Relationship

1) Can you describe your experience of the interaction between yourself and the doctors/nurses or others you have seen?
2) Can you describe the role of the nurse/doctor?
3) Can you describe the relationship you had with the nurse?
    - What do you talk about with the nurses – what do they say to you?
4) In what ways would the relationship differ between a nurse visiting your relative at home compared to on a ward?
5) Home/respiratory specialist nurse, what do you think about this?
6) Did you understand what was required from you? Concerns?
7) Anything missing/any disadvantages?
8) What would have made it better for you?
9) What went particularly well or what were the main benefits for you?

Nebulisers

1) What do you understand about nebulisers/inhalers?
2) Can you describe a situation in which your relative has needed to use one - in (mode of treatment) - What's the difference in using one at home versus in hospital?
3) Why would your relative use a nebuliser/inhaler, what would trigger its usage, anything in particular?
4) Is there any issue of using one at home? Are you using it more often/over using it/is it better at home?
5) How often do you find they use a nebuliser/inhaler at home?
6) What are the advantages/disadvantages of nebulisers/inhalers?

Summary question

1) Anything else you would like to tell me or anything else you think I should know?

Interview questions – Health care professionals (clinician interviews)

1) Can you begin by telling me what you understand about hospital at home (HAH)?
2) Would you be able to share your views of HAH – What do you feel about home treatment?
3) How did you become involved in the project and how long for/would you like more involvement?
4) Can you describe your role? And what affect if any the project has on it?
5) Have you been involved in the treatment of patients at home/in hospital – can you describe this?

6) What do you think about when considering if a patient is eligible for HAH?
7) Have you had any patients that you haven't put through to the trial/any situation where you would not recommend a patient for HAH?
8) What's the difference in care if any a patient receives in HAH?
9) Organisation wise how do you feel the project is running?
10) What were your expectations of HAH? Have these changed?
11) Is there anything that is going particularly well about it or what are the main benefits you can see?
12) Do you see any disadvantages or can you recommend an area of improvement?
13) Do you have any preferences as to where to treat patients?
14) What do you think about the future implementation?
15) Is there anything else you think I should know or anything you would like to tell me?

Interview schedule for managers

1) Can you begin by telling me what you understand about hospital at home (HAH)?
2) Would you be able to share your views of hospital at home – how do you feel about home treatment as a service for patients?
3) With regard to the current RCT of HAH for patients with an exacerbation of COPD, can you describe your role and your involvement in the trial?
4) From the perspective of this Trust, how do you think the trial is running?
5) What are your views on using DECAF to identify low risk patients?
6) Is there any conflict between clinician judgement and DECAF?
7) What were your expectations of HAH? Have these changed?
8) Can you recommend an area of improvement?
9) What are the advantages from a patients'/clinicians' perspective?
10) What are the disadvantages from a patients'/clinicians' perspective?
11) Is there anything that is going particularly well about it or what are the main benefits you can see?
12) Can you describe the role of a respiratory nurse?
13) Can you describe the interaction between the respiratory nurses and consultants?
14) What are the advantages, and challenges, from the perspective of a hospital manager or director/ hospital information services and management?
15) Have there been any issues with the trial?
16) How do we establish and support hospital at home as a clinical service?
17) What support do you think the clinical staff need to effectively implement the service?
18) Should it be possible, if the trial results are positive and no major issues are experienced, that this could be used as a model to establish and implement similar services in other trusts?
19) Are there any barriers towards implementation? If so, how do you think these should be tackled?
20) What do you think of it being a seven day service?
21) If other trusts are to implement the trial how do we support this?
22) What level of central engagement is needed if the trial is successful to make it easier for other trusts?

23) Anything else you would like to discuss?

Interview Questions for patients

Experience

1) Can you describe your experience of how you came to be in hospital?

Project information

1) Can you tell me your initial thoughts when asked to participate in the study?
2) Initially you stated a preference for (mode of treatment). Has your preference changed following your recent experience?
3) Initially your reasons for preferring (mode of treatment) were (quote) can you explain this in more detail?
4) How did you find (mode of treatment)?
5) If in the future HAH is routinely available, would you choose to have treatment at home or in hospital? Why?
6) What were your expectations previously and was your experience different from these?
7) How did you feel when at (mode of treatment)?
8) How would you have felt if treated (mode of treatment)?
9) How do you feel when the nurse or doctor comes to visit you at (mode of treatment)/would this differ if you were (in other mode of treatment)?
10) If any of your friends had any problems would you suggest to them to go for HAH?
11) Would there be circumstances with your friends where you think, "This would not suit this person but it suits me?"
12) Did you ever have to ring the nurse whilst at home – what was your experience?
13) Can you describe the role of the doctors/nurses or others you have seen?
    - Did you understand what was required from you? Concerns?
    - Anything missing/any disadvantages?
    - What would have made it better for you?
    - What went particularly well or what were the main benefits for you?

Nebulisers

1) Can you describe a situation in which you have needed to use a nebuliser? - Hospital/at home?
2) What's the difference between using one at home versus in hospital?
3) Why would you use one at home/what would trigger its usage, anything in particular?
4) (at home) Are you using it more often/over using it/how do you find using it/how often?
5) Any situation where you would prefer not to use one? Why?
6) What are the benefits/disadvantages?
7) Summarise - Is there anything else you would like to tell me?

Interviews Nurses

1) Can you begin by telling me what you understand about hospital at home (HAH)?
2) Would you be able to share your views of HAH – What do you feel about home treatment?

3) How did you become involved in the project – what's you role?
4) What affect does the project have on your role as a nurse – how has it changed your role?
5) Are you happy with your involvement would you like more/less?

Treatment

1) Have you been involved in the treatment of patients at home– can you describe this?
2) What's it like visiting patients in their homes/how do you feel when there?
3) How does this differ to treating someone in hospital? What's the main difference to you?
4) Have you ever done supported discharge visits?
5) How does HAH compare to this – similarities/differences?
6) Is there any anxiety over one or the other – why?
7) How do you feel making decisions for patient's treatment?
8) Have you had a situation where it has not benefited the patient or can you see were this may happen?

Nebulisers

1) Is there a difference in nebuliser treatment in patients at HAH versus UC?
2) Is there a difference in how patients use them? Why?
3) Is there any reluctance to take the nebuliser away from those at home? Difference in how you use them?
4) How often do patients use them at home? What happens if there not used?
5) Any issues with nebulisers that you can see?
6) Do you have a preference as to why you prefer to treat patients?

The project

1) Organisation wise how do you feel the project is running?
2) How has it changed normal procedures – what's now different about it?
3) Is there anything that is different about it that people may not necessarily agree on?
4) Are there any issues with the HAH patients having their clinical notes?
5) What were your expectations of HAH? Have these changed?
6) Is there anything that is going particularly well about it or what are the main benefits you can see?
7) Do you see any disadvantages or can you recommend an area of improvement?
8) How do you feel over all – pleasure/burden? Concerns?
9) What do you think about the future implementation?
10) What are your views on the on call respiratory physician?
11) How has this impacted?

Relationships

1) Can you describe the relationship between yourself and the patients when in a patient's home?
2) What do you speak to patients about?
3) What do they tell you?
4) Can you describe the relationship with patients when seeing them on the ward?
5) What's the relationship like with patients at home/compared to hospital?
6) Is there a difference in relationships between men and women?

7) Ever feel intimidated or are you ever more careful?
8) What do you feel is important for a good working relationship with patients?

Hospital A

1) What are you views on Hospital A?
2) How has Hospital A impacted the process of HAH? Easier/harder?

Anything else?
