# Supplementary material for: What are the positive drivers and potential barriers to implementation of hospital at home selected by low-risk DECAF score in the UK: a qualitative study embedded within a randomised controlled trial
Source: BMJ Open. 2019 Apr 4;9(4):e026609. doi: 10.1136/bmjopen-2018-026609 (PMC6500229; doi:10.1136/bmjopen-2018-026609)
Supplement: Author's manuscript [file bmjopen-2018-026609.draft_revisions.pdf]

BMJ Open is committed to open peer review. As part of this commitment we make the peer review history of every article we publish publicly available.

When an article is published we post the peer reviewers' comments and the authors' responses online. We also post the versions of the paper that were used during peer review. These are the versions that the peer review comments apply to.

The versions of the paper that follow are the versions that were submitted during the peer review process. They are not the versions of record or the final published versions. They should not be cited or distributed as the published version of this manuscript.

BMJ Open is an open access journal and the full, final, typeset and author-corrected version of record of the manuscript is available on our site with no access controls, subscription charges or pay-per-view fees (http://bmjopen.bmj.com).

If you have any questions on BMJ Open's open peer review process please email info.bmjopen@bmj.com

BMJ Open

Positive drivers and potential barriers to implementation of hospital at home selected by low risk DECAF score.

Journal:	*BMJ Open*
Manuscript ID	bmjopen-2018-026609
Article Type:	Research
Date Submitted by the Author:	18-Sep-2018

Complete List of Authors:	Dismore, Lorelle; Northumbria Healthcare NHS Foundation Trust, Research and Development; Teesside University, School of Social Sciences, Humanities and Law<br>Echevarria, Carlos; Northumbria Healthcare NHS Foundation Trust, Respiratory<br>van Wersch, Anna; Teesside University, School of Social Sciences, Humanities and Law<br>Gibson, John; Newcastle university, Institute of Cellular Medicine<br>Bourke, Stephen; Northumbria Healthcare NHS Foundation Trust, Respiratory

Keywords:	QUALITATIVE RESEARCH, Quality in health care < HEALTH SERVICES ADMINISTRATION & MANAGEMENT, Protocols & guidelines < HEALTH SERVICES ADMINISTRATION & MANAGEMENT, Respiratory infections < THORACIC MEDICINE

SCHOLARONE™ Manuscripts

Positive drivers and potential barriers to implementation of hospital at home selected by low risk DECAF score.

Authors

Corresponding author and lead author

Lorelle Louise Dismore[1]

Department of Research and Development, North Tyneside General Hospital, Rake Lane, North Shields, Tyne and Wear NE29 8NH, UK, 0191 293 4087;

lorelle_dismore@hotmail.com

Additional authors

Carlos Echevarria,[1,3] Anna van Wersch,[2] John Gibson,[3] Stephen Bourke[1,3]

[1] Northumbria Healthcare NHS Foundation Trust, Tyne and Wear, UK, [2]Teesside University, Middlesbrough, UK, [3]Newcastle University, Newcastle upon Tyne, UK.

Key words; qualitative, COPD, exacerbation, service development

Word count: 4402

For peer review only

Abstract

Objective

Hospital at home (HAH) for COPD exacerbation selected by DECAF score is clinically and cost effective. Up to 50% of admitted patients are suitable; a much larger proportion than earlier services. Introduction of new models of care is challenging, but may be facilitated by informed engagement with stakeholders.

Design

Facilitators and barriers to implementation of HAH were sought using semi-structured interviews with 44 patients, 15 carers, 15 physicians, 11 specialist nurses and 4 managers. Thematic-Construct Analysis was applied.

Results

Facilitators include: 1) availability of home comforts and maintaining independence (with positive influences on perceived rate of recovery, sleep quality and convenience for friends, family and carers); and 2) confidence in the continuity of HAH care.

Barriers include: 1) fear of being alone at home; 2) privacy issues and not wanting visitors; and 3) resistance to change. Clinician concerns occasionally delayed return home, principally during the early phase of the trial. Nurses cited higher workload and greater responsibility, but with additional resource and training overall they viewed HAH positively. Operational concerns included keeping medical records in a patient's home and inability to capture activity within current payment systems.

Conclusion

HAH selected by DECAF was preferred to inpatient care by most patients and their families. Implementation in other hospitals will require education, training and service planning, tailored to overcome the identified barriers.

ISRCTN29082260.

Strengths and limitations

- A large number of patients were interviewed, including those who declined involvement in the randomised controlled trial.
- To minimise the risk of observer bias, interviews were conducted by a health psychologist separate from the clinical team.
- Managers and clinicians identified operational concerns with this model of care; effective methods to address these are discussed.
- The results of this study strongly support, and will help facilitate, wider implementation of this model of care.

For peer review only - http://bmjopen.bmj.com/site/about/guidelines.xhtml

Introduction

In a randomised controlled trial (RCT) we have shown that Hospital at Home (HAH) selected by the DECAF prognostic score is cost-effective, safe, and preferred by 90% of patients.[1] DECAF is a prognostic score that can be used to risk assess patients with a Chronic Obstructive Pulmonary Disease exacerbation (ECOPD) using indices that are routinely available at admission: Dyspnoea, Eosinopenia, Consolidation, Acidaemia and atrial Fibrillation. It offers excellent performance and identifies a large proportion of patients (45-53%) as low risk (DECAF 0-1) and therefore candidates for HAH.[2, 3] This is of importance as ECOPD account for over one million “bed days” in UK hospitals each year and are associated with substantial morbidity and cost.[4-6]

In ECOPD, key differences of HAH to early supported discharge (ESD) are the inclusion of patients with higher medical dependency, replacing all or most of the inpatient stay, and the provision of 24/7 support. NICE endorse both services in patients with a low risk of death.[7] In an era of spiralling healthcare demands and costs, providers are open to new models of care.[8] Qualitative studies aid implementation of novel clinical services and add validity to the results of RCTs.[9, 10] Within our RCT, we undertook an embedded qualitative study to identify positive drivers and potential barriers to HAH for ECOPD selected by DECAF and assist wider implementation.

Methods

Participants

Participants included patients within the HAH and usual care (UC) arms of the RCT, their carers, patients who declined enrolment in the RCT, clinicians and hospital managers. HAH and UC patients were purposively selected (CE, LD) in relation to gender, age, socio-economic background, COPD severity (eMRCD, FEV1, previous admissions) and reasons for their preferred place of care. All respiratory specialist nurses (RSN) and consultants, and key acute physicians and managers were invited. Ethical approval was granted by NRES Committee North East-Sunderland (Ref: 13/NE/0275) and the trial was registered (ISRCTN: 290082260). Participants received a telephone call to confirm participation in interviews, informed consent was obtained for the interviews, and all participants were anonymised/ given pseudonyms.

Semi-structured interviews were carried out with 89 participants, which included 44 patients, 15 carers, 15 physicians, 11 RSNs and 4 managers. Eight patients and carers were interviewed together at their request; all other interviews were one-to-one. Most interviews were conducted within the participant’s home and a minority were inpatients at the time of interview. Participant characteristics are shown in Table 1. The term “decliners” refers to patients who declined participation in the RCT, but consented to interview within this study. Interviews were conducted until data saturation was reached.

Patients participating in the RCT who had a carer (unpaid individual who helped with daily needs) were identified. Carers were asked to complete the Zarit Burden Interview at baseline, 14 days and 90 days. The Zarit Burden Interview is a multidimensional scale that measures caregiver burden. We planned to present the median values and data range by group, without inferential statistics.

For peer review only - http://bmjopen.bmj.com/site/about/guidelines.xhtml

Interviews

Prior to the RCT, the interview schedules were developed within a pilot HAH service and further informed by feedback from inpatients recovering from ECOPD. The final semi-structured interview schedule (online supplement), contained a series of open-ended questions, such as, *"If in the future HAH is routinely available, would you choose to have treatment at home or in the hospital and why?"*

The interviews were designed to explore patients' experience of receiving HAH and UC, preference and perceived influencing factors. The role of the carer within both structures of care and carer-burden were assessed. Clinical and organisational factors that impact on professional's experiences were explored. Patient and carer interviews were arranged post discharge, and all interviews were audio-recorded and transcribed verbatim.

In the subgroup of decliners, interviews were shorter and more focussed on issues related to participation with HAH.

Participants experiences of HAH and UC were explored, with an aim to uncover the drivers and barriers towards the new care pathway, therefore the transcripts were analysed using a combined inductive-deductive method known as "Thematic-Construct Analysis".[11] Data were read for communalities working from a critical perspective.

Researchers remained as faithful as possible to the participants' own accounts using an inductive approach, whilst looking deductively for discourses that were in line with positive drivers and potential barriers of the new care pathway. Each interview transcript was read and re-read by three authors (LD, AvW, SB) to ensure familiarisation with the data; the authors independently coded the data and identified themes. The interpretations were discussed, and the final themes and subthemes were agreed upon (see Table 2).

Table 1: Description of participants

Participant role	n	Description
Patients participating	31	16 from UC, 15 from HAH. Mean (SD) age= 68 (9.9) years; 52% female; median (IQR) eMRCD score= 4 (3-4); mean (SD) FEV1= 43.4 (18.7) per cent predicted and previous admissions for an exacerbation= 0 (0-2).
Decliners	13	Mean (SD) age= 73 (10.8) years; 77% female; median (IQR) eMRCD score= 4 (4-4).
Carers	15	Mean (SD) age= 61 (11.3) years, 53% female, 80% were the patient's partner. 5 carers' relatives received UC and 10 carers' relatives received HAH.
RSNs	11	Mean (SD) age= 39 (10.8) years, mean (SD) years' experience= 7 (5) years.
Physicians	15	11 respiratory consultants, 3 acute care physicians, and 1 specialist registrar; mean (SD) age= 41.5 (6) years, 71.4% were male, mean (SD) years reported experience = 12.5 (5.6)
Managers	4	3 secondary and 1 social care; mean (SD) age= 50 (1.8) years, 50% were female.

Table 2: Social constructions of Hospital at Home

For peer review only - http://bmjopen.bmj.com/site/about/guidelines.xhtml

1. Positive Drivers for HAH

- 1.1 Availability of home comforts and maintaining independence
  - 1.1.1 Perception of a quicker recovery with HAH and positive effects on perceived breathlessness
  - 1.1.2 Improved sleep and nutrition
  - 1.1.3 More convenient place to be with friends and family
- 1.2 Confidence in the continuity of HAH care
  - 1.2.1 Feelings of safety, reassurance and appreciation
  - 1.2.2 Personalised relation and specialist expertise of the RSN

2. Potential Barriers and Negative Influences for HAH Care Pathway

- 2.1 Fear of being alone at home
- 2.2 Privacy issues and not wanting visitors
- 2.3 Resistance to change
  - 2.3.1 Reluctance to accept removal of nebulised therapy
  - 2.3.2 Challenging clinician's preconceptions, accepting a new model of care and operational concerns
- 2.4 Negative influences of HAH

3. Other Insights

- 3.1 Unintentional change in UC
- 3.2 Early uncertainty with HAH selected by DECAF

Member-checking was performed with physicians due to non-familiarity of the new care model. All physicians were sent their own transcripts and asked whether they still agreed with their responses to which three agreed to be interviewed again. Member checking was not performed in other groups because interviews with patients and nurses were longer, they were all familiar with the care provided, numbers were larger, and we did not want to overburden individuals.

Decliners were interviewed (with consent) to elicit their reasons for declining enrolment in the RCT and whether they would consider HAH in the future, if shown to be safe and effective.

Clinical structure of care

Hospital at Home

HAH provides care for patients within their home for a condition that would otherwise require inpatient care.[12] HAH involved treating patients at home supported by a nurse-led respiratory specialist service, after a brief inpatient assessment. Patients were not regarded as sufficiently well for discharge. Further information on the service is available including structured clinical assessment sheets and the HAH manual within the online supplement of the RCT.[1]

Usual Care

Patients were managed in accordance with usual hospital care. We engaged with clinical staff, emphasising that clinical decisions should not be influenced by trial participation.

For peer review only - http://bmjopen.bmj.com/site/about/guidelines.xhtml

Patient and public involvement

The semi-structured research interviews, intervention (HAH), and outcome measures were informed by interviews with patients and family members, including carers. Most were happy with treatment within HAH provided this was safe. Key concerns with HAH were: clinical deterioration at home, delayed treatment, and lack of social support/ increased carer burden. Direct telephone contact, 24/7, to the respiratory team and the availability of same day social support addressed these issues. Furthermore, we used a validated tool to assess carer burden. An expert patient and non-expert patients informed the selection of health related questionnaires and measures of service acceptability. Patients with AECOPD assessed different home-monitoring devices for acceptability and comfort and reviewed the written description of HAH for clarity which informed the patient information sheet.

Results

Quotes which best presented the established constructs were identified by capital letters using scripts from patients (P), decliners (D), carers (C), consultants (Co), RSN (N) and managers (M). The Respiratory Specialist Registrar's comments were coded Co for anonymity. Quotations have been shortened; both the full quotation and additional supporting quotations are provided in the outline supplement. All patients that were interviewed survived to the end of the study.

1. Positive Drivers for Hospital at Home

1.1 Availability of home comforts and maintaining independence

Patients, carers and RSNs, consistently highlighted that patients were comfortable in their home, with positive influences on mood and confidence. Hospital routines were avoided, and patients maintained their independence by engagement in usual activities. Patients requiring oxygen noted that the equipment provided within HAH allowed free movement, whilst provision in hospital limited mobility. "*I couldn't even walk up the ward… I only had this like little lead… I can go upstairs [at home]… it's nice and I can get up and make myself a meal" (P22L145-153).*

1.1.1 Perception of a quicker recovery with HAH and positive effects on perceived breathlessness

Patients and carers perceived that recovery occurred more quickly during HAH, and breathlessness was less marked, despite higher activity levels. This may reflect lower levels of anxiety within HAH: *"Just so relaxed… content with the care… because when you're anxious obviously it reacts on your breathing and your whole persona" (P6L109-112).* Compared to UC, delays in obtaining reliever therapy were also avoided within HAH.

1.1.2 Improved sleep and nutrition

Patients reported sleep disruption in UC related to disturbances from other patients, nurses obtaining regular observations and new admissions, whilst those who experienced HAH reported improved sleep: *"There's no bed like your own bed" (N19L129).*

Opinions were diverse concerning nutrition although most favoured HAH. *"(I) liked hospital food and ate more in hospital than at home" (P10L97)* and, *"You don't eat the same in hospital for a start" (P1L18).*

For peer review only - http://bmjopen.bmj.com/site/about/guidelines.xhtml

1.1.3 More convenient place to be with friends and family

Home is more convenient and saves time and money for family and friends. Travel, car parking, work absences, childcare issues, restrictive visiting hours and hospital TV costs are avoided. *"It's easier for my family to visit… getting cars parked… when they have been to work all day by the time they get the hospital it's time to come home" (P22L21-24).*

A key benefit of HAH was increased time with grandchildren: "*When I'm in hospital I don't see the grandchildren. I've got six… and three of them live next door and they're here every single day. When I'm not here they do cry; they get really upset" (P13L167-169).*

Carers reported that providing care at home was less disruptive, by avoiding hospital visits which take more time and organisation: "*It's better my husband being at home… instead of… going back and forward to the hospitals and especially with being a carer looking after two children" (P17 L537-548).*

1.2 Confidence in the continuity of HAH care

1.2.1 Feelings of safety, reassurance and appreciation

Patients felt safe and reassured during HAH due to daily visits from the RSNs, the 24-hour telephone support line, confidence in the HAH clinical team, and the availability of emergency services if return to hospital was needed. The evening phone call (9pm) was reassuring to those patients living alone: *"I had confidence in the team that came out... they all seemed to me to be very well trained and putt me at ease" (P1L71-74)*, and, *"If they thought you needed a doctor or the hospital they would phone an ambulance straight away" (P12L320-327).*

Most patients were not unduly concerned about potential delays in being seen by a doctor/clinician in the event of deterioration. *"If your hospital at home, if you take really poorly yes you phone up… they will still be here as quick as they would if you were in a hospital" (P12L319-327).*

Within HAH, patients appreciated the ability to take their medication at their usual time, with immediate access to reliever therapy.

1.2.2 Personalised relation and specialist expertise of the RSN

Patients in HAH appreciated the better continuity in healthcare professionals compared to inpatient care and described the relationship with RSN as "personal", "individual" and "one to one care". Clinical assessments conducted at home provided privacy and were less rushed with "time to develop rapport". Patients valued the clear explanations provided by RSNs during HAH about all aspects of their care, including monitoring, investigations, and management, commenting that this may be lacking in hospital.

Patients experienced a lack of dignity and privacy during UC. *"A wash down behind the curtain, I was frightened I was rushing" (P32L53-55).* UC patients acknowledged time constraints as a barrier towards personalised care, which was also recognised by the RSNs.

Discharge from UC incurs delays due to waiting for medication, discharge letter and transport. Such concerns were not raised about transfer home under HAH or subsequent discharge.

For peer review only - http://bmjopen.bmj.com/site/about/guidelines.xhtml

2. Potential Barriers and Negative Influences for HAH Care Pathway

2.1 Fear of being alone at home

A common reason for declining participation in the RCT was fear of being alone when unwell, even when offered (same day) social support. The RSN stated that some patients were reluctant to accept social services because of perceived stigmatisation. "*The hours I would be on my own… say I got up to go the toilet and my legs went what do you do then if you can't breathe… you've got no phone at hand" (D1, P2L39-41)* and, *"They think if they've got a social worker or are involved with social services it's like they are labelled... they wouldn't realise they could just ring social services and ask for help" (N4L102-104).*

Participants recognised there may be a longer delay getting help if they deteriorated at home compared to in-hospital care. *"Some people maybe prefer to be in a cocoon of a hospital environment; they maybe worry if something goes wrong someone's seconds away from them" (P1L226-229).*

2.2 Privacy issues and not wanting visitors

Other decliners referred to privacy issues. "*I thought people coming to my house, I cannot… I'm frightened that it wouldn't be tidy*" (D3L4-7). For some, personal circumstances affected participation: *"My husband and I are divorcing and my house just having been sold the atmosphere at home wouldn't be conducive in recuperating… but in the future... I would very much like to be involved" (D9L14-21).*

2.3 Resistance to change

2.3.1 Reluctance to accept removal of nebulised therapy

During HAH patients received similar treatment to those within UC, including controlled oxygen therapy, nebulised bronchodilators and intravenous medications if required. Patients reported that nebulised bronchodilators helped chest clearance as well as relieving breathlessness. Patients in HAH were more likely to object to the removal of nebulised therapy than controlled oxygen. Similar objections were not raised to the same extent in UC. The challenge for the RSN was to reassure patients that such interventions were usually not required long term. *"I think a lot of them have the misconception that if they have a nebuliser they are better… Your inhalers are far better more practical" (N3L222-229).*

2.3.2 Challenging clinicians' preconceptions, accepting a new model of care and operational concerns

Clinicians' preconceptions included the view that hospital care provides respite for carers and ensures patients feel safer. *"The patient themselves may not be ready for it… I think they need to stay in hospital where they feel safer not because it is safer but basically they feel safer and also it gives a break (to) their carer" (Co2 L53-66).* Another physician disagreed, stating: *"That's kind of often how people view patients with COPD that they want to come to hospital but actually it's not true" (Co3 L198-201).*

Operational concerns included keeping medical records in a patient's home and the inability to capture activity within current payment systems. *"Our work in trying to get the centre to change the way… we can record the data for hospital at home patients… perhaps by us doing that it would make it easier for other trusts to go down the hospital home route in the future" (M1L149-153)* and, *"If we*

For peer review only - http://bmjopen.bmj.com/site/about/guidelines.xhtml

are employing consultants, junior doctors, respiratory nurses, specialists nurses... and we are not having the patient in hospital so we are not getting the tariff for that… somebody has to pay the wage bill… and it's about understanding that payment mechanism… then that becomes a lot easier for everybody because that is the kind of thing that will stop people moving forward" (M17L183-193).

A different model of care requires adjustment. The RSNs advised that their confidence increased with experience of delivering HAH, whilst patients mentioned that the quality of the service was dependent on the skill of the attending RSN. This highlights the importance of adequate training and support. *"The only concern I would have is that hospital at home is only as good as the nurses you've got on" (P12L336-337).*

2.4 Negative Influences of HAH

Patients felt less inclined to smoke in hospital. *"When I'm in hospital… I don't think about smoking whatsoever where… at home I do"" (P14L273-276).*

The hospital ward is an opportunity to establish new friendships that may continue post discharge and patients were concerned that their relatives might be frightened seeing/ knowing that they are unwell at home. *"I've made some nice friends… still ring them.. It was nice" (P22L497-505). "It's frightening for them to see when you're not well it's not nice for my husband to sit and watch us when I'm bad" (P25L220-222).*

3. Other Insights

3.1 Unintentional change in UC

Despite efforts to ensure UC was not influenced by trial participation, patients were discharged home earlier than expected. Factors include clinician awareness of low risk of death by DECAF, pressure from patients who were disappointed with allocation to UC and pressure from operational service managers. *"There's much more emphasis on getting people home early… the people who we aren't (treating) in the hospital at home I think people are now more likely to send them home a lot earlier" (Co3 L120-126).*

3.2. Early uncertainty with HAH selected by DECAF

Initially some clinicians expressed concern that, in their judgement, true risk for an individual patient was underestimated by DECAF. This led to delays in return home, which on occasion was unsettling for patients: *"They have certain reservations, so you've got that conflict between their clinical impression and the predictive score…the simple score almost always outperforms clinical judgement, but clinicians don't like to think that's true" (Co18L40-52).* With experience clinicians became more confident using DECAF and the safety within HAH model of care: *"As you get evidence suggesting it works and it's safe you're more confident in doing it" (Co6L154-155).* This was reflected in the wider HAH population as delayed return home was primarily an issue at the start of the trial.

"I think part of the main advantage from my point of view is making me more aware of the safety of the DECAF score and the ability that people can be treated out of the hospital… whether that's because of the hospital at home trial or… because of the gradual change in practice because of the DECAF score I'm not sure" (Co6L99-103).

For peer review only - http://bmjopen.bmj.com/site/about/guidelines.xhtml

For peer review only

Carers

In the RCT, 23 carers in HAH and 18 carers in UC completed Zarit Burden Interviews, including 13 of 15 carers participating in this embedded qualitative study. Higher scores show higher levels of care burden. Baseline scores were similar in UC and HAH, but at 14 and 90 days scores were lower in HAH (table 3).

Table 3: Zarit Burden scores in carers

		Count, n	Median (IQR)	% improved or same v baseline	Missing
Zarit Burden 0 day	UC	18	18 (10-29)	Ref	1
	HAH	23	16 (10-30)	Ref	0
Zarit Burden 14 day	UC	18	23 (16-32)	35.7	3
	HAH	23	13 (8-31)	57.9	4
Zarit Burden 90 day	UC	18	25 (13-35)	16.7	5
	HAH	23	14.5 (10-36)	27.8	5

The percentage of patients with no change or an improvement in carer burden scores was higher in HAH, although this could be a chance finding.

Discussion

We interviewed 89 participants including patients, carers, and healthcare professionals to inform implementation of our HAH model. Whilst our RCT provides the justification for the implementation of HAH regarding cost and clinical outcome, this qualitative study identifies the human and organisational factors that will influence its successful implementation. Positive drivers included: greater independence and freedom; the maintenance of usual activities and perceived shorter recovery time; the maintenance of contact with friends and family (especially grandchildren); better sleep; and more time spent with an expert healthcare professional. Reported barriers from patients included being alone at night and having “strangers” visiting the home. Of note, some of these barriers, such as those relating to privacy and not wanting visitors, may be less of an issue at institutions which already have early supported discharge.

Early concerns from some physicians and RSNs diminished with experience of successful delivery of this model of care. Two patients that were allocated to HAH were kept in hospital at the consultant’s discretion; this occurred in the first quarter of the study. Managers highlighted the inability for HAH activity to be captured within current payment systems. Hospitals planning to implement HAH selected by DECAF should pre-emptively address these issues. Compared to ESD, patients within HAH have higher clinical needs. Respiratory and Emergency Care Physicians require education, particularly highlighting the reliability of the DECAF score. RSNs delivering care at home require additional clinical training, consultant support and capacity to deliver the service. The HAH Manual and assessment sheets used within our service are available to facilitate this process. Of note, training costs were included in the health economic analysis, which showed that this model of care is cost-effective. Maintaining clinical documents in a patient’s home should be supported by local agreement and patient consent.

For peer review only - http://bmjopen.bmj.com/site/about/guidelines.xhtml

The study has several key strengths. We used a qualitative approach with semi-structured interviews to explore the issues around HAH for those involved in the service [13]. We performed a large number of interviews using purposeful sampling of patients to ensure both a wide range of participants and perspectives [14]. Not only were the experiences of patients and carers explored, but operational concerns from managers and clinicians were identified. Of note, we interviewed patients who declined involvement in the trial: this is a group rarely included in such studies. The study sites had no HAH service prior to the RCT, so the emergent themes relate to HAH as a new service. This supports the transferability of our results to other institutions which lack this service. Finally, the views of carers were triangulated with the Zarit Burden Interview.

The study has several limitations. As with all qualitative research, it is impossible to fully remove observer bias. To minimise this risk, interviews were performed by a female trained health psychologist who was separate from the clinical team and had no previous experience or knowledge in the design of the HAH care pathway, and three individuals independently performed analyses of the data to increase trust-worthiness and inter-rated reliability. Certain aspects, such as operational concerns, may only apply to the UK healthcare system. Finally, there is a risk of social desirability bias, in that interviewees may tell us what they think we want to hear. This was minimised by the interviewer emphasising that they were not involved in clinical care and that frank discourse would help develop the service.

Previous RCTs of domiciliary treatment for patients with ECOPD had more exclusion criteria than our RCT, and mostly were of ESD rather than HAH.[15] Our RCT included patients typically deemed too unwell for home treatment in earlier studies, such as those with pneumonic ECOPD. Perceived risk remains a key factor for engagement, particularly during the early phase of service introduction, which will be important to address during educational sessions. In the DECAF study cohorts, 1,266 of 2,645 patients were DECAF 0-1 (low risk), and risk of death remained low regardless of the index scoring. In the HoT DECAF RCT, there were no acute deaths.[1] This is also in line with patient and carer perceptions in other trials. In a study of elderly patients, which included patients with ECOPD, substitutive HAH was reported as being safe by family and carers.[16] Similar findings were shown in a study of six patients with ECOPD.[17] In our study, patients and carers reported that HAH was safe, noting confidence in the clinical team and the availability of 24/ 7 telephone support, with return to hospital if required.

Five of thirteen patients who declined enrolment in the RCT reported fear of being home alone due to breathlessness, anxiety and/ or problems coping at home. Three patients regretted not enrolling, and three declined partly because of unwell family members in the home. Despite the high rates of pneumonia and co-morbidity, overall healthcare professionals were satisfied that the DECAF score selected low risk patients suitable for HAH. In the whole HAH population (n=60), there were only three instances of discordance between clinician judgement and the DECAF score leading to patients randomised to HAH receiving UC, all of whom survived to 90 days follow up.

Carers of patients with COPD are at risk of various psychological health problems, such as anxiety and depression; partners of patients with COPD are typically elderly with their own health problems.[18] Utens and others looked at carer strain in an RCT of ESD compared to usual care, and showed no difference in the caregiver strain index between allocation groups.[19] Given the higher treatment needs of patients in HAH, we anticipated that the carers of patients treated with HAH

For peer review only - http://bmjopen.bmj.com/site/about/guidelines.xhtml

would report increased carer burden. However, carers reported that inpatient stay was more disruptive than HAH, both in terms of time and cost. This was supported by the Zarit Burden Interview scores, which showed that the median carer burden scores were numerically lower with HAH than UC, though this could have been a chance finding. Whilst patients may express a fear of being or becoming a burden on their carers at the end of life,[20] this was not reported in any of the interviews with patients in the context of HAH for an acute exacerbation.

HAH selected by DECAF allows the inclusion of more patients than existing models, is preferred to inpatient care by most patients and their families, and is considered to be safe. HAH and the associated care was valued by many patients, in particularly with regards to continuity of care and in maintaining individuals' independence. Following this study, HAH has been commissioned as a service for all patients with low-risk ECOPD. The results of this study, both in terms of potential drivers and barriers, are important areas of discussion when offering HAH to patients.

For peer review only

For peer review only - http://bmjopen.bmj.com/site/about/guidelines.xhtml

Acknowledgements

This paper presents independent research funded by the National Institute for Health Research (NIHR) under its Research for Patient Benefit (RfPB) Programme (Grant Reference Number PB-PG-0213-30105). The views expressed are those of the authors and not necessarily those of the NHS, the NIHR or the Department of Health.

We thank Teresa Gibson for her input to the trial management group as an expert patient, the members of the Data Monitoring Committee (Rodney Hughes, Nick S Hopkinson and Niall Anderson) and the Trial Steering Committee (Nicholas Hart, Jennifer Quint, Jadwiga Wedzicha, Patrick Murphy, Robert Rutherford). We thank Tracey Finch for her input in the initial draft of the project.

We are grateful to all interviewees for their time and candour.

Contributors

SCB conceived the study and was chief investigator with overall responsibility for the management of the study. SCB, AvW and CE were responsible for the original design and protocol, and obtaining funding. LLD and CE recruited patients to the study. LLD performed all interviews and transcripts. SCB, AvW, and LLD analysed all transcripts for themes. CE performed statistical analyses. LLD and AvW wrote the first draft of the manuscript, and all authors were involved in the final version.

Funding

NIHR Research for Patient Benefit and Northumbria Healthcare NHS Foundation Trust Teaching and Research Fellowship programmes.

Competing interests

All authors have completed a ICMJE uniform disclosure. SCB reports grants from NIHR: Research for Patient Benefit programme, during the conduct of the study; HTA funding, grants from Philips Respironics, grants from Pzifer Open Air, personal fees from Pzifer and AztraZeneca outside the submitted work. AvW reports grants from NIHR: Research for Patient Benefit programme, during the conduct of the study. LD, CE and GJG have no competing interests to declare.

Patient consent

Obtained

Ethics approval

Ethics approval was provided by NRES Committee North East Sunderland (3/NE/0275). All participants gave informed consent before taking part in the study.

For peer review only

References

1. Echevarria C, Gray J, Hartley T, et al. Home treatment of COPD exacerbation selected by DECAF score: a non-inferiority, randomised controlled trial and economic evaluation. *Thorax* 2018.
2. Steer J, Gibson, J. & Bourke, S. The DECAF Score: predicting hospital mortality in exacerbations of chronic obstructive pulmonary disease. *British Medical Journal* 2012; **67**(11): 970-6.
3. Echevarria C SJ, Heslop-Marshall K, Stenton SC, Hickey PM, Hughes R, Wijesinghe M, Harrison RN, Steen N, Simpson AJ, Gibson GJ, Bourke SC. Validation of the DECAF score to predict hospital mortality in acute exacerbations of COPD. *Thorax* 2016; **71**: 133-40.
4. Society. BT. The Burden of Lung Disease. . In: Society BT, editor. (2nd edition) ed; 2006.
5. Seemungal TA, Donaldson GC, Paul EA, Bestall JC, Jeffries DJ, Wedzicha JA. Effect of exacerbation on quality of life in patients with chronic obstructive pulmonary disease. *American Journal of Respiratory & Critical Care Medicine* 1998; **157**(5 Pt 1): 1418-22.
6. Vestbo J, Hurd SS, Agusti AG, et al. Global strategy for the diagnosis, management, and prevention of chronic obstructive pulmonary disease: GOLD executive summary. *Am J Respir Crit Care Med* 2013; **187**(4): 347-65.
7. Excellence NIfHaC. Chronic obstructive pulmonary disease in over 16s: diagnosis and management. In: Excellence NIfHaC, editor. England: NICE; 2010.
8. England N. 5 year forward view. 2014. https://www.england.nhs.uk/wp-content/uploads/2014/10/5yfv-web.pdf.
9. Campbell M, Fitzpatrick R, Haines A, et al. Framework for design and evaluation of complex interventions to improve health. *BMJ (Clinical research ed)* 2000; **321**(7262): 694-6.
10. Bradley F, Wiles R, Kinmonth AL, Mant D, Gantley M. Development and evaluation of complex interventions in health services research: case study of the Southampton heart integrated care project (SHIP). The SHIP Collaborative Group. *BMJ (Clinical research ed)* 1999; **318**(7185): 711-5.
11. Dismore L, van Wersch, A & Swainston, K. Social constructions of the male contraception pill: When are we going to break the vicious circle? *Journal of Health Psychology* 2016; **21**(5): 788-97.
12. Shepperd SI, S. . Hospital-at-home versus in-patient hospital care. *Cochrane Database Systematic Review* 2001; (2).
13. Greenhalgh T, Taylor R. Papers that go beyond numbers (qualitative research). *BMJ (Clinical research ed)* 1997; **315**(7110): 740-3.
14. Bryman A. Social Research Methods: Oxford University Press; 2008.
15. Echevarria C, Brewin K, Horobin H, et al. Early Supported Discharge/ Hospital At Home For Acute Exacerbation Of Chronic Obstructive Pulmonary Disease: A Review and Meta-Analysis. *COPD* 2016: 1-11.
16. Leff B, Burton, L., Mader, S. L., Naughton, B., Burl, J., Koehn, D., Clark, R., Greenough, W., William, B, I., Guido, S., Steinwachs, D. & Burton, J. R. . Comparison of stress experienced by family members of patients treated in hospital at home with that of those receiving traditional acute hospital care. *Journal of the American Geriatrics Society* 2008; **56**(1): 117-23.
17. Wang Y, Torbjorn, H., Steihaug, S. & Werner, A. Patients with acute exacerbation of chronic obstructive pulmonary disease feel safe when treated at home: a qualitative study. *BMC Pulmonary Medicine* 2012; **12**(1): 1.
18. Gardiner C, Gott M, Small N. Living with advanced chronic obstructive pulmonary disease: patients concerns regarding death and dying. *Palliative Medicine* 2009; **23**: 691-7.
19. Utens CM, van Schayck OC, Goossens LM, et al. Informal caregiver strain, preference and satisfaction in hospital-at-home and usual hospital care for COPD exacerbations: results of a randomised controlled trial. *Int J Nurs Stud* 2014; **51**(8): 1093-102.
20. Jones I, Kirby A, Ormiston P, et al. The needs of patients dying of chronic obstructive pulmonary disease in the community. *Fam Pract* 2004; **21**(3): 310-3.

For peer review only - http://bmjopen.bmj.com/site/about/guidelines.xhtml

For peer review only

For peer review only - http://bmjopen.bmj.com/site/about/guidelines.xhtml

Table of themes with quotations

Online supplementary report of full and additional quotations related to themes. Interviewer quotes are shown in bold.

Theme 1. Positive Drivers for HAH	Quotes
1.1 Availability of home comforts and maintaining independence	I couldn't even walk up the ward or anything you know because I only had this like little lead you know and I used to keep forgotten walking off then if you wanted to talk to anybody you had to either shout or they had to come to you you know but that's the only thing just sort of sat there all the time (P22L145-148)<br><br>**And now in the home how would you describe this situation with the oxygen lead?**<br><br>Oh yes I can go upstairs do different pieces up there you know tidy my draws up and that's you know just a couple at a time just err no its nice and I can get up and make myself a meal you know (P22L145-153)<br><br>Yeah well what can you do in hospital you're either lying on a bed or sitting in a chair I can walk back and forward in here you know the and I can go upstairs and down bath wash shave that type of thing you know were as well you can do that in hospital get washed and shaved and that but you're stuck in the one place you're not going anywhere you know you're not getting no exercise nothing (P18L114-118)<br><br>In a lot of ways you're disempowering the patient by keeping them on a ward and keeping them within that sort of very tight sort of role, when you get them home then they're they're dressed, they are doing more things themselves (N7L293-296)
1.1.1 Perception of a quicker recovery during HAH and positive effects on perceived breathlessness	Her recovery time was much quicker so it was easier for me because she was helping herself do you know what I mean (CP28L260-261)<br><br>Just so relaxed content, content with the care everything just made everything because when your anxious obviously it reacts on your breathing and your whole persona really and just everything was so relaxed I don't know how else to describe it (P6L109-112)
1.1.2 Improved sleep and nutrition	People recover better in their own home and there was one lady and I quote her often to the staff there's no bed like your own bed and that stuck because actually she was right absolutely right you know why be sitting in

For peer review only

	a hospital bed when you've got poorly people around you if you have that support to be able to recuperate at home (M19L128-132).<br><br>I was very surprised at the food it wasn't too bad. In fact I had more in the hospital than I did at home (P10L92-97)<br><br>You don't eat the same in hospital for a start and you certainly don't sleep the same you can be awake most of the night (P6L91-92)
1.1.3 More convenient place to be with friends and family	It's easier for my family to visit and everything you know getting cars parked you know when they have been to work all day by the time they get the hospital it's time to come home you know so it's definitely benefitted me you know (P22 L21-24)<br><br>But even the grand children when I'm in hospital I don't see the grandchildren I've got six grandchildren and three of them live next door and they're here every single day when I'm not here they do cry they get really upset (P13 L167-169)<br><br>They don't like fetching them in when they're so young I mean the youngest ones four and the twins and they miss us when I'm not here (P16L87-88)<br><br>It's better my husband being at home and getting the treatment at home instead of like going back and forward to the hospitals and especially with being a carer looking after two children as well you know it's a lot better<br><br>**So when you say you look after two children as well, so are you a carer for those two children and Jim?**<br><br>Yes<br><br>**So hospital at home how does that make it easier in regards to that?**<br><br>Like with looking after Jim and getting the support and like for visit and things you know like when he's at home he's at home and getting looked after at home and I can think of the children and it doesn't put as much

For peer review only

	pressure on us as well because like when you're visiting and you've got children to pick up at school as well you know it's all like the time as well (P23 L537-548)<br><br>It's been much better for me anyway because I used to get knackered go into hospital every day and coming back then having to go back again and coming back whereas been here I can go to work come in do whatever I wanted to do when I knew she was safe she was all right in the house do you know what I mean (P28L225-228)<br><br>Financial yes yes because you see were on a low income my partner only works part time window cleaner and we have to pay full rent and everything and money is really tight and for him to keep coming back and forth to hospital I mean sometime if there's no money he can't go you know so that's the benefits because he even though he's a part time window cleaner he can still go to work when I'm poorly he works local (P13L56-61)<br><br>At first I was a bit concerned because I look after the grandchildren not every day four days a week sometimes erm and I was more concerned about them whether it would interfere with personal life sort of thing err and then went and thought about it it obviously wouldn't I'd get to see them more than what I would actually been in hospital so that was the deciding factor really (P6L12-16)
1.2 Confidence in the continuity of HAH care<br>1.2.1 Feelings of safety, reassurance and appreciation	I had confidence in the team that came out I think I saw four different people over a short period of time they all seemed to me to be very well trained and putting me at ease and I really didn't feel like I was missing anything that I would have had in hospital you know (P1L70-73)<br><br>Now I feel what's the word more secure I don't know yeah more secure because I know all I've got to do is make a phone call like obviously there's name of doctor there's obviously the nurses if I have to there would be you there is more people to talk to now and that's something I never had before which is good' (P13L103-106)<br><br>Few have said but are you not nervous X like if you're in a hospital you're on a ward they get a doctor to you as soon as they can and they might not get it as soon as you can might be two three hours down the line but if your hospital at home if you take really poorly yes you phone up you've got that number they will still be here as quick as they would if you were in a hospital do you know what I mean it doesn't mean to say because you're on a ward that a nurse or a doctors going to get to you do you know and I would phone a the district nurse or community nurse and if they thought you needed a doctor or the hospital they would phone an ambulance straight away (P12L321-328)

For peer review only

	Telephone call before I came out I got a telephone call before the afternoon in the evening check that things were okay I was told on that evening who would be coming the next day so I knew who to expect so I don't have a problem at all with it (P1 P7 L320-323)
1.2.2 Personalised relation and specialist expertise of the RSN	A wash down behind the curtain I was frightening I was rushing (P32L53-55)<br><br>I mean you cannot get individual care can you really because there's 16 on the ward another five people in care and of course at home you're getting the care more attention (P16 L163-165)<br><br>They checked everything every day you know everything ankles everything whereas a rule it's just your blood pressure and your oxygen level in the hospital and your erm temperature yes but I don't know they were just really brilliant they were here quite a long time you know making sure I was feeling all right and I was confident on my own you know but no I found them really good and there are so nice you know really nice and caring (P22L44-49)<br><br>I'll be perfectly honest the times I've been in the hospital the nurses there been too busy or there's only been one on or two on and they can't do anything you know there to busy doing other things you don't get a one to one erm but in your home you do get one to one (P12L208-211)<br><br>She made me feel safe she made me feel I was getting a proper treatment erm comfortable is the word she made me feel really comfortable knew what she was doing explained everything even down to when you were getting your stats done you know when you were getting while you were getting your bloods taken erm explained everything you don't always get that in a hospital (P12L365-370)<br><br>You get told you can get out tomorrow and then they say you've got tablets and everything and by the time its tea time you're still waiting there somebody coming to get you or getting the tablets or getting the letter but everything's so well done there was didn't get you harassed at all and it just seemed like you go home today you've got your tablets you've got your letter you were taken down to the departure place the nurse was there they had been with the oxygen so well planned it was like err just sail a long and I like that because it didn't get as well erm and it made you feel more relaxed and thinking to yourself well if this is how it's going to be then that's okay I can cope (P20L423-431)
2. Potential Barriers and	

For peer review only

Negative Influences of HAH Care Pathway	
2.1 Fear of being alone at home	The hours I would be on my own you know if just say I got up to go the toilet and my legs went what do you do then if you can't breathe you know you've got no phone at hand you know you cannot pull an alarm switch or anything so no (Decliner 1 P2L39-41)<br><br>Maybe it's the generation thing I mean mention a social working oh no I haven't got one of them but I mean you know I think they think if there've got a social worker or they're involved with social services it's like there labelled but it's not that but they don't realise that so you know until its explained to them so there would be sat at home and they wouldn't realise they could just ring social services and ask for help (N4L100-104)<br><br>Some people maybe prefer to be in a cocoon of a hospital environment, they maybe worry if something goes wrong someone's seconds away from them (P1L226-229)
2.2 Privacy issues and not wanting visitors	I thought people coming to my house, I cannot I'm frightened that it wouldn't be tidy and things like that, that's what type of person I am I'm frightened that it wouldn't be tidy (D3L4-7)<br><br>My husband and I are divorcing and my house just having been sold the atmosphere at home wouldn't be conducive in recuperating…but in the future … I would very much like to be involved (D9L14-21).
2.3 Resistance to change<br>2.3.1 Reluctance of the removal of nebulised therapy	The nebulisers seem to clear you and they clear you quickly and they seem as if erm you get relief after you've had them (P12L90-91)<br><br>I mean I take all my self-medication at home anyway but you know I'm on steroids and antibiotics and so I've got them at home anyway as I say it's just if I had a nebuliser at home that would sort of cure everything I think (P7L57-59)<br><br>I would still rather have home treatment you know if I had a nebuliser I would be happy as Larry I would be because once I'm on that and take a couple of doses I'm fine (P7L69-71)<br><br>I feel safer actually with that there (P27L311-313)

For peer review only

	I think a lot of them have the misconception that if they have a nebuliser they're better and that seems to stick in their mind so of course then we have to go down do you realise it increased your heart rate its higher mortality rate ideally it's for emergency you shouldn't be having them all the time so we do sort of stress to them you don't want to be on this treatment your inhalers are far better more practical more easier to use so we do try our best to get them off (N3L223-229)
2.3.2 Challenging clinicians' preconceptions, accepting a new model of care and operational concerns	The patient themselves may not be ready for it I may be ready for it but the patient may be two days away from it and also sometimes the patient needs would like if someone came into a hotel for their illness for a while they want to be away from their sick bed because their home is almost like a toxic area so all I'm doing is what I'm trying to tell the patient is I can actually bring the hospital to your home with some patients it's a good idea but some patients say actually I want to take a break from my home<br><br>**So is that the major difference in those patients who want to stay in hospital?**<br><br>Yeah because I think they need to stay in hospital where they feel safer not because it is safer but basically they feel safer and also it gives a break their carers would like to get rid of them for two or three days there's many different things going on in the patient the mind of the patient<br>(Co2 P2L53-66)<br><br>That's kind of often how people view patients with COPD that they want to come to hospital but actually it's not true (Co3 L198-201)<br><br>Our work in trying to get the centre to change the way erm we can record the data for hospital at home patients erm perhaps by us doing that it would make it easier for other trusts to go down the hospital home route in the future (M1P4 L151-153)<br><br>If we're employing consultants junior doctors respiratory nurses specialists nurses and health cares we're not having the patient in hospital so we're not getting the tariff for that but we're sending those patients out to look after them somebody has to pay the wage bill for that and it's about understanding that payment mechanism and if that's absolutely crystal clear then that becomes a lot easier for everybody because that's the kind of thing that will stop people moving forward (M17L188-193)

For peer review only

	Think the only concern I would have is that hospital at home is only as good as the nurses you've got on (P12L336-337)
2.4 Negative Influences of HAH	I mean for all I'm a smoker when I'm in hospital, things like that, I don't think about smoking whatsoever where I am at home I do but erm whenever I'm in a strange place like that I never smoking never crosses my mind (P14L273-276).<br><br>I've made some nice friends when I was in for a week you know still ring them you know erm but that was nice but I didn't wander round and talk to them you know but erm as I say erm the first time I went in it was nice (P22L497-499)<br><br>It's frightening for them to see when you're not well it's not nice for my husband to sit and watch us when I'm bad (P25L220-222)
3. Other Insights<br>3.1 Unintentional change in UC	There's a much more emphasis on getting people home early and I think even the people who are in the usual care arm so the people who we aren't doing in the hospital at home I think people are now more likely to send them home a lot earlier than they did when I did my stuff four years ago so I think general practice has changed we're not hanging on to these patients as long as we did erm and that's through external pressures erm but you know external pressures make you want to get people home as quickly as possible but the trial itself might be running the trial might also be changing peoples practice and may be making them send people home earlier (Co3 L121-129)
3.2 Early uncertainty with HAH selected by DECAF	They have certain reservations so you've got that conflict between there clinical impression and the predictive score. There are patients where as I said their risk is low but the clinical judgement is that they are not low risk now the difficulty here is that we shouldn't necessarily ignore our clinician judgement because well it's important and it's important to listen to however again and again the studies show that clinical judgement is not as good as prognostic scores so you've got research from the 1950s and literally hundreds of studies now from medicine and kind of baseball wine-making you name it completely different types of erm different types of expertise the simple score almost always out performs clinical judgement but clinicians don't like to think that's true (Co18L40-52).<br><br>For some cases it seems obviously a good thing to do then there's other cases where previously you might have

For peer review only

	thought better keep those inpatients in for a few days rather than sending them home so a couple of patients were that's realise that's different from what you normally do and you do think not anxiety but you do think oh I hope that things are going to alright but that's why we're doing a trial because we don't know (Co3L22-27)<br><br>As you see it works and as you get evidence suggesting it works and its safe you're more confident in doing it (Co6L154-155)<br><br>I think part of the main advantage from my point of view is making me more aware of the safety of the DECAF score and the ability that people can be treated out of the hospital so more whether that's because of the hospital at home trial or whether that's just because of the gradual change in practice because of the DECAF score I'm not sure (Co6L99-103)

For peer review only

Interview questions – Carers

Background to the carer

1) Can you describe your relationship to (X)?
2) Can you describe the care you give at home?
3) What sort of care do you provide?
4) Can you describe a typical day/how much of your day does it take?
5) How has your life changed since you have been providing care?
    - Has it affected your daily routine (what was it like before/how is it now – how would you describe what you do – how do you feel)?
    - What's good or bad about it?
    - Is it becoming easier/harder – in what ways?
    - What do you find hardest about caring for someone? (loneliness/fear/isolation/financial difficulties)
6) How has care changed over time?
7) Is there a change when your relative's health suddenly worsens?
8) What helps you in providing care?
    - (coping/support/faith/family)
    - Do you receive any help/support from anyone?
10) How do you feel about providing care? (burden/guilty/helpless)
11) What do you think about someone from a social services team coming in to help provide care?
12) What does the word carer mean to you/how do you feel about the word carer?

Project Questions

1) What are your perceptions of hospitals?
2) Has your relative been previously hospitalised?
3) Can you describe past experiences of hospital?
4) How do you feel if your relative has to go into hospital?
5) Can you describe a typical day on which your relative is hospitalised – what type of care do you provide?
6) Can you tell me what your initial thoughts were when you were told about HAH?
7) Initially your reasons for preferring home treatment were (QUOTE). Can you explain this in more detail?
8) How did you find (mode of treatment)?
    - Expectations of (mode of treatment)/different from your experience?
9) How was it being at (mode of treatment) with your relative?
10) How would you have felt if (X) was treated (mode of treatment)?
11) Can you describe what happened whilst (X) was being treated (mode of treatment)?
    - What did they do whilst at home? Relaxed or not?
12) What is your preference now following your recent experience?
13) If in the future HAH is available, would you choose to have your relative treated at home or in hospital? Why?

For peer review only

14) Can you describe the care you give your relative comparing when at home and hospitalised? – any differences?
15) Where would you prefer to care for your relative?
16) How does it benefit you?
17) Any disadvantages of having to care for your relative at (mode of treatment)?
18) What concerns would you have in the future if your relative is treated at (mode of treatment)?

Dr-patient Relationship

1) Can you describe your experience of the interaction between yourself and the doctors/nurses or others you have seen?
2) Can you describe the role of the nurse/doctor?
3) Can you describe the relationship you had with the nurse?
    - What do you talk about with the nurses – what do they say to you?
4) In what ways would the relationship differ between a nurse visiting your relative at home compared to on a ward?
5) Home/respiratory specialist nurse, what do you think about this?
6) Did you understand what was required from you? Concerns?
7) Anything missing/any disadvantages?
8) What would have made it better for you?
9) What went particularly well or what were the main benefits for you?

Nebulisers

1) What do you understand about nebulisers/inhalers?
2) Can you describe a situation in which your relative has needed to use one - in (mode of treatment) - What's the difference in using one at home versus in hospital?
3) Why would your relative use a nebuliser/inhaler, what would trigger its usage, anything in particular?
4) Is there any issue of using one at home? Are you using it more often/over using it/is it better at home?
5) How often do you find they use a nebuliser/inhaler at home?
6) What are the advantages/disadvantages of nebulisers/inhalers?

Summary question

1) Anything else you would like to tell me or anything else you think I should know?

Interview questions – Health care professionals (clinician interviews)

1) Can you begin by telling me what you understand about hospital at home (HAH)?
2) Would you be able to share your views of HAH – What do you feel about home treatment?
3) How did you become involved in the project and how long for/would you like more involvement?
4) Can you describe your role? And what affect if any the project has on it?
5) Have you been involved in the treatment of patients at home/in hospital – can you describe this?

For peer review only

6) What do you think about when considering if a patient is eligible for HAH?
7) Have you had any patients that you haven't put through to the trial/any situation where you would not recommend a patient for HAH?
8) What's the difference in care if any a patient receives in HAH?
9) Organisation wise how do you feel the project is running?
10) What were your expectations of HAH? Have these changed?
11) Is there anything that is going particularly well about it or what are the main benefits you can see?
12) Do you see any disadvantages or can you recommend an area of improvement?
13) Do you have any preferences as to where to treat patients?
14) What do you think about the future implementation?
15) Is there anything else you think I should know or anything you would like to tell me?

Interview schedule for managers

1) Can you begin by telling me what you understand about hospital at home (HAH)?
2) Would you be able to share your views of hospital at home – how do you feel about home treatment as a service for patients?
3) With regard to the current RCT of HAH for patients with an exacerbation of COPD, can you describe your role and your involvement in the trial?
4) From the perspective of this Trust, how do you think the trial is running?
5) What are your views on using DECAF to identify low risk patients?
6) Is there any conflict between clinician judgement and DECAF?
7) What were your expectations of HAH? Have these changed?
8) Can you recommend an area of improvement?
9) What are the advantages from a patients'/clinicians' perspective?
10) What are the disadvantages from a patients'/clinicians' perspective?
11) Is there anything that is going particularly well about it or what are the main benefits you can see?
12) Can you describe the role of a respiratory nurse?
13) Can you describe the interaction between the respiratory nurses and consultants?
14) What are the advantages, and challenges, from the perspective of a hospital manager or director/ hospital information services and management?
15) Have there been any issues with the trial?
16) How do we establish and support hospital at home as a clinical service?
17) What support do you think the clinical staff need to effectively implement the service?
18) Should it be possible, if the trial results are positive and no major issues are experienced, that this could be used as a model to establish and implement similar services in other trusts?
19) Are there any barriers towards implementation? If so, how do you think these should be tackled?
20) What do you think of it being a seven day service?
21) If other trusts are to implement the trial how do we support this?
22) What level of central engagement is needed if the trial is successful to make it easier for other trusts?

For peer review only

23) Anything else you would like to discuss?

Interview Questions for patients

Experience

1) Can you describe your experience of how you came to be in hospital?

Project information

1) Can you tell me your initial thoughts when asked to participate in the study?
2) Initially you stated a preference for (mode of treatment). Has your preference changed following your recent experience?
3) Initially your reasons for preferring (mode of treatment) were (quote) can you explain this in more detail?
4) How did you find (mode of treatment)?
5) If in the future HAH is routinely available, would you choose to have treatment at home or in hospital? Why?
6) What were your expectations previously and was your experience different from these?
7) How did you feel when at (mode of treatment)?
8) How would you have felt if treated (mode of treatment)?
9) How do you feel when the nurse or doctor comes to visit you at (mode of treatment)/would this differ if you were (in other mode of treatment)?
10) If any of your friends had any problems would you suggest to them to go for HAH?
11) Would there be circumstances with your friends where you think, "This would not suit this person but it suits me?"
12) Did you ever have to ring the nurse whilst at home – what was your experience?
13) Can you describe the role of the doctors/nurses or others you have seen?
    - Did you understand what was required from you? Concerns?
    - Anything missing/any disadvantages?
    - What would have made it better for you?
    - What went particularly well or what were the main benefits for you?

Nebulisers

1) Can you describe a situation in which you have needed to use a nebuliser? - Hospital/at home?
2) What's the difference between using one at home versus in hospital?
3) Why would you use one at home/what would trigger its usage, anything in particular?
4) (at home) Are you using it more often/over using it/how do you find using it/how often?
5) Any situation where you would prefer not to use one? Why?
6) What are the benefits/disadvantages?
7) Summarise - Is there anything else you would like to tell me?

Interviews Nurses

1) Can you begin by telling me what you understand about hospital at home (HAH)?
2) Would you be able to share your views of HAH – What do you feel about home treatment?

For peer review only

3) How did you become involved in the project – what's you role?
4) What affect does the project have on your role as a nurse – how has it changed your role?
5) Are you happy with your involvement would you like more/less?

Treatment

1) Have you been involved in the treatment of patients at home– can you describe this?
2) What's it like visiting patients in their homes/how do you feel when there?
3) How does this differ to treating someone in hospital? What's the main difference to you?
4) Have you ever done supported discharge visits?
5) How does HAH compare to this – similarities/differences?
6) Is there any anxiety over one or the other – why?
7) How do you feel making decisions for patient's treatment?
8) Have you had a situation where it has not benefited the patient or can you see were this may happen?

Nebulisers

1) Is there a difference in nebuliser treatment in patients at HAH versus UC?
2) Is there a difference in how patients use them? Why?
3) Is there any reluctance to take the nebuliser away from those at home? Difference in how you use them?
4) How often do patients use them at home? What happens if there not used?
5) Any issues with nebulisers that you can see?
6) Do you have a preference as to why you prefer to treat patients?

The project

1) Organisation wise how do you feel the project is running?
2) How has it changed normal procedures – what's now different about it?
3) Is there anything that is different about it that people may not necessarily agree on?
4) Are there any issues with the HAH patients having their clinical notes?
5) What were your expectations of HAH? Have these changed?
6) Is there anything that is going particularly well about it or what are the main benefits you can see?
7) Do you see any disadvantages or can you recommend an area of improvement?
8) How do you feel over all – pleasure/burden? Concerns?
9) What do you think about the future implementation?
10) What are your views on the on call respiratory physician?
11) How has this impacted?

Relationships

1) Can you describe the relationship between yourself and the patients when in a patient's home?
2) What do you speak to patients about?
3) What do they tell you?
4) Can you describe the relationship with patients when seeing them on the ward?
5) What's the relationship like with patients at home/compared to hospital?
6) Is there a difference in relationships between men and women?

For peer review only

7) Ever feel intimidated or are you ever more careful?
8) What do you feel is important for a good working relationship with patients?

Hospital A

1) What are you views on Hospital A?
2) How has Hospital A impacted the process of HAH? Easier/harder?

Anything else?

For peer review only

COREQ (COnsolidated criteria for REporting Qualitative research) Checklist

A checklist of items that should be included in reports of qualitative research. You must report the page number in your manuscript where you consider each of the items listed in this checklist. If you have not included this information, either revise your manuscript accordingly before submitting or note N/A.

Topic	Item No.	Guide Questions/Description	Reported on Page No.
**Domain 1: Research team and reflexivity**			
Personal characteristics			
Interviewer/facilitator	1	Which author/s conducted the interview or focus group?	10
Credentials	2	What were the researcher's credentials? E.g. PhD, MD	10
Occupation	3	What was their occupation at the time of the study?	10
Gender	4	Was the researcher male or female?	10
Experience and training	5	What experience or training did the researcher have?	10
Relationship with participants			
Relationship established	6	Was a relationship established prior to study commencement?	NO
Participant knowledge of the interviewer	7	What did the participants know about the researcher? e.g. personal goals, reasons for doing the research	
Interviewer characteristics	8	What characteristics were reported about the inter viewer/facilitator? e.g. Bias, assumptions, reasons and interests in the research topic	10
**Domain 2: Study design**			
Theoretical framework			
Methodological orientation and Theory	9	What methodological orientation was stated to underpin the study? e.g. grounded theory, discourse analysis, ethnography, phenomenology, content analysis	4
Participant selection			
Sampling	10	How were participants selected? e.g. purposive, convenience, consecutive, snowball	3
Method of approach	11	How were participants approached? e.g. face-to-face, telephone, mail, email	3
Sample size	12	How many participants were in the study?	3-4
Non-participation	13	How many people refused to participate or dropped out? Reasons?	NONE
Setting			
Setting of data collection	14	Where was the data collected? e.g. home, clinic, workplace	3
Presence of non-participants	15	Was anyone else present besides the participants and researchers?	NO
Description of sample	16	What are the important characteristics of the sample? e.g. demographic data, date	4
Data collection			
Interview guide	17	Were questions, prompts, guides provided by the authors? Was it pilot tested?	3
Repeat interviews	18	Were repeat inter views carried out? If yes, how many?	4
Audio/visual recording	19	Did the research use audio or visual recording to collect the data?	4
Field notes	20	Were field notes made during and/or after the inter view or focus group?	
Duration	21	What was the duration of the inter views or focus group?	30-60
Data saturation	22	Was data saturation discussed?	3
Transcripts returned	23	Were transcripts returned to participants for comment and/or	

Topic	Item No.	Guide Questions/Description	Reported on Page No.
		correction?	4
**Domain 3: analysis and findings**			
Data analysis			
Number of data coders	24	How many data coders coded the data?	10
Description of the coding tree	25	Did authors provide a description of the coding tree?	
Derivation of themes	26	Were themes identified in advance or derived from the data?	4
Software	27	What software, if applicable, was used to manage the data?	[illegible]
Participant checking	28	Did participants provide feedback on the findings?	
Reporting			
Quotations presented	29	Were participant quotations presented to illustrate the themes/findings? Was each quotation identified? e.g. participant number	5 – 9
Data and findings consistent	30	Was there consistency between the data presented and the findings?	
Clarity of major themes	31	Were major themes clearly presented in the findings?	5 – 9
Clarity of minor themes	32	Is there a description of diverse cases or discussion of minor themes?	6, 7, 8,

Developed from: Tong A, Sainsbury P, Craig J. Consolidated criteria for reporting qualitative research (COREQ): a 32-item checklist for interviews and focus groups. *International Journal for Quality in Health Care*. 2007. Volume 19, Number 6: pp. 349 – 357

Once you have completed this checklist, please save a copy and upload it as part of your submission. DO NOT include this checklist as part of the main manuscript document. It must be uploaded as a separate file.

BMJ Open

What are the positive drivers and potential barriers to implementation of hospital at home selected by low risk DECAF score in the UK, a qualitative study embedded within a randomised controlled trial.

Journal:	*BMJ Open*
Manuscript ID	bmjopen-2018-026609.R1
Article Type:	Research
Date Submitted by the Author:	23-Jan-2019
Complete List of Authors:	Dismore, Lorelle; Northumbria Healthcare NHS Foundation Trust, Research and Development; Teesside University, School of Social Sciences, Humanities and Law<br>Echevarria, Carlos; Northumbria Healthcare NHS Foundation Trust, Respiratory<br>van Wersch, Anna; Teesside University, School of Social Sciences, Humanities and Law<br>Gibson, John; Newcastle university, Institute of Cellular Medicine<br>Bourke, Stephen; Northumbria Healthcare NHS Foundation Trust, Respiratory
**Primary Subject Heading**:	Qualitative research
Secondary Subject Heading:	Respiratory medicine
Keywords:	QUALITATIVE RESEARCH, Quality in health care < HEALTH SERVICES ADMINISTRATION & MANAGEMENT, Protocols & guidelines < HEALTH SERVICES ADMINISTRATION & MANAGEMENT, Respiratory infections < THORACIC MEDICINE, qualitative, COPD, exacerbation, service development

SCHOLARONE™ Manuscripts

What are the positive drivers and potential barriers to implementation of hospital at home selected by low risk DECAF score in the UK, a qualitative study embedded within a randomised controlled trial.

Authors

Corresponding author and lead author

Lorelle Louise Dismore[1]

Department of Research and Development, North Tyneside General Hospital, Rake Lane, North Shields, Tyne and Wear NE29 8NH, UK, 0191 293 4087;

lorelle_dismore@hotmail.com

Additional authors

Carlos Echevarria,[1,3] Anna van Wersch,[2] John Gibson,[3] Stephen Bourke[1,3]

[1] Northumbria Healthcare NHS Foundation Trust, Tyne and Wear, UK, [2]Teesside University, Middlesbrough, UK, [3]Newcastle University, Newcastle upon Tyne, UK.

Key words; qualitative, COPD, exacerbation, service development

Word count: 4402

For peer review only

Abstract

Objective

Hospital at home (HAH) for COPD exacerbation selected by low-risk DECAF score is clinically and cost effective; DECAF is a prognostic score indicating risk of mortality. Up to 50% of admitted patients are suitable; a much larger proportion than earlier services. Introduction of new models of care is challenging, but may be facilitated by informed engagement with stakeholders. This qualitative study sought to identify facilitators and barriers to implementation of HAH.

Design

Semi-structured interviews, data were analysed using Thematic-Construct Analysis.

Setting

Interviews were conducted within patients' homes and hospitals in North East England.

Participants

Eight nine participants were interviews; 44 patients, 15 carers, 15 physicians, 11 specialist nurses and 4 managers.

Results

Facilitators include: 1) availability of home comforts and maintaining independence (with positive influences on perceived rate of recovery, sleep quality and convenience for friends, family and carers); and 2) confidence in the continuity of HAH care.

Barriers include: 1) fear of being alone at home; 2) privacy issues and not wanting visitors; and 3) resistance to change. Clinician concerns occasionally delayed return home, principally during the early phase of the trial. Nurses cited higher workload and greater responsibility, but with additional resource and training overall they viewed HAH positively. Operational concerns included keeping medical records in a patient's home and inability to capture activity within current payment systems.

Conclusion

HAH selected by DECAF was preferred to inpatient care by most patients and their families. Implementation in other hospitals will require education, training and service planning, tailored to overcome the identified barriers.

ISRCTN29082260.

Strengths and limitations

- A large number of interviews were performed using purposeful sampling to ensure a wide range of participants and perspectives.
- Member checking was performed on physicians.
- Interviews were conducted by a health psychologist separate to the clinical team to minimise risk of observer bias.

For peer review only - http://bmjopen.bmj.com/site/about/guidelines.xhtml

- There is a risk of social desirability bias with interviewees telling us that which we wish to hear.
- The study was conducted within a UK healthcare setting and may not be representative to other countries implementing a HAH care pathway.

For peer review only

For peer review only - http://bmjopen.bmj.com/site/about/guidelines.xhtml

Introduction

In a randomised controlled trial (RCT) we have shown that Hospital at Home (HAH) selected by the DECAF prognostic score is cost-effective, safe, and preferred by 90% of patients.[1] DECAF is a prognostic score that can be used to assess the inpatient risk of death in those with a Chronic Obstructive Pulmonary Disease exacerbation (ECOPD) using indices that are routinely available at admission: Dyspnoea, Eosinopenia, Consolidation, Acidaemia and atrial Fibrillation. It offers excellent performance and identifies a large proportion of patients (45-53%) as low risk (DECAF 0-1) and therefore candidates for HAH.[2, 3] This is of importance as ECOPD account for over one million "bed days" in UK hospitals each year and are associated with substantial morbidity and cost.[4-6]

In ECOPD, key differences of HAH to early supported discharge (ESD) are the inclusion of patients with higher medical dependency, replacing all or most of the inpatient stay, and the provision of 24/7 support. The National Institute for Health and Care Excellence (NICE) endorse both services in patients with a low risk of death.[7] In an era of spiralling healthcare demands and costs, providers are open to new models of care.[8] Qualitative studies aid implementation of novel clinical services and add validity to the results of RCTs.[9, 10] Within our RCT, we undertook an embedded qualitative study to identify positive drivers and potential barriers to HAH for ECOPD selected by DECAF and assist wider implementation.

Methods

Participants

Participants included patients within the HAH and usual care (UC) arms of the RCT, their carers, patients who declined enrolment in the RCT, clinicians and hospital managers. HAH and UC patients were purposively selected (CE, LD) in relation to gender, age, socio-economic background, COPD severity (i.e. breathlessness by the extended, modified research council dyspnoea score; forced expiratory flow volume in one second; and previous admissions) and reasons for their preferred place of care. All respiratory specialist nurses (RSN) and consultants, and key acute physicians and managers were invited. Ethical approval was granted by NRES Committee North East-Sunderland (Ref: 13/NE/0275) and the trial was registered (ISRCTN: 290082260). Participants received a telephone call to confirm participation in interviews, informed consent was obtained for the interviews, and all participants were anonymised/ given pseudonyms.

Semi-structured interviews were carried out with 89 participants, which included 44 patients, 15 carers, 15 physicians, 11 RSNs and 4 managers (Hospital: General Manager, operational service manager, and finance. Community: Adult Social Care manager). Eight patients and carers were interviewed together at their request; all other interviews were one-to-one. One study participant was interviewed in hospital, all other participants and carers were interviewed in their home. Participant characteristics are shown in Table 1. The term "decliners" refers to patients who declined participation in the RCT, but consented to interview within this study. Most decliners were interviewed in hospital. Interviews were conducted until data saturation was reached.

For peer review only - http://bmjopen.bmj.com/site/about/guidelines.xhtml

Patients participating in the RCT who had a carer (unpaid individual who helped with daily needs) were identified. Carers were asked to complete the Zarit Burden Interview at baseline, 14 days and 90 days. The Zarit Burden Interview is a multidimensional scale that measures caregiver burden. We planned to present the median values and data range by group, without inferential statistics.

Interviews

Prior to the RCT, the interview schedules were developed within a pilot HAH service and further informed by feedback from inpatients recovering from ECOPD. The final semi-structured interview schedule (online supplement), contained a series of open-ended questions, such as, "*If in the future HAH is routinely available, would you choose to have treatment at home or in the hospital and why?"*

The interviews were designed to explore patients' experience of receiving HAH and UC, preference and perceived influencing factors. The role of the carer within both structures of care and carer-burden were assessed. Clinical and organisational factors that impact on professional's experiences were explored. Patient and carer interviews were arranged post discharge, and all interviews were audio-recorded and transcribed verbatim.

In the subgroup of decliners, interviews were shorter and more focussed on issues related to participation with HAH.

Participants experiences of HAH and UC were explored, with an aim to uncover the drivers and barriers towards the new care pathway, therefore the transcripts were analysed using a combined inductive-deductive method known as "Thematic-Construct Analysis".[11] Data were read for communalities working from a critical perspective.

Researchers remained as faithful as possible to the participants' own accounts using an inductive approach, whilst looking deductively for discourses that were in line with positive drivers and potential barriers of the new care pathway. Each interview transcript was read and re-read by three authors (LD, AvW, SB) to ensure familiarisation with the data; the authors independently coded the data and identified themes. The interpretations were discussed, and the final themes and subthemes were agreed upon (see Table 2).

Table 1: Description of participants

Participant role	n	Description
Patients participating	31	16 from UC, 15 from HAH. Mean (SD) age= 68 (10) years; 52% female; median (IQR) eMRCD score= 4 (3-4); mean (SD) FEV1= 43.4 (18.7) per cent predicted and previous admissions for an exacerbation= 0 (0-2).
Decliners	13	Mean (SD) age= 73 (11) years; 77% female; median (IQR) eMRCD score= 4 (4-4).
Carers	15	Mean (SD) age= 61 (11) years, 53% female, 80% were the patient's partner. 5 carers' relatives received UC and 10 carers' relatives received HAH.
RSNs	11	Mean (SD) age= 39 (11) years, 100% female, mean (SD) years' experience= 7 (5) years.

For peer review only - http://bmjopen.bmj.com/site/about/guidelines.xhtml

Physicians	15	11 respiratory consultants, 3 acute care physicians, and 1 specialist registrar; mean (SD) age= 42 (6) years, 71.4% were male, mean (SD) years reported experience = 12 (6) years.
Managers	4	3 hospital and 1 social care; mean (SD) age= 50 (2) years, 50% were female.

RSNs, Respiratory specialist nurses; eMRCD, breathlessness score; UC, usual care; HAH, hospital at home; FEV1, forced expiratory volume.

Member-checking was performed with physicians due to non-familiarity of the new care model. All physicians were sent their own transcripts and asked whether they still agreed with their responses to which three agreed to be interviewed again. Member checking was not performed in other groups because interviews with patients and nurses were longer, they were all familiar with the care provided, numbers were larger, and we did not want to overburden individuals.

Decliners were interviewed (with consent) to elicit their reasons for declining enrolment in the RCT and whether they would consider HAH in the future, if shown to be safe and effective.

Clinical structure of care

Hospital at Home

HAH provides care for patients within their home for a condition that would otherwise require inpatient care.[12] HAH involved treating patients at home supported by a nurse-led respiratory specialist service, after a brief inpatient assessment. Patients were not regarded as sufficiently well for discharge. Further information on the service is available including structured clinical assessment sheets and the HAH manual within the online supplement of the RCT.[1]

Usual Care

Patients were managed in accordance with usual hospital care. We engaged with clinical staff, emphasising that clinical decisions should not be influenced by trial participation.

Patient and public involvement

The semi-structured research interviews, intervention (HAH), and outcome measures were informed by interviews with patients and family members, including carers. Most were happy with treatment within HAH provided this was safe. Key concerns with HAH were: clinical deterioration at home, delayed treatment, and lack of social support/ increased carer burden. Direct telephone contact, 24/7, to the respiratory team and the availability of same day social support addressed these issues. Furthermore, we used a validated tool to assess carer burden. An expert patient and non-expert patients informed the selection of health related questionnaires and measures of service acceptability. Patients with AECOPD assessed different home-monitoring devices for acceptability and comfort and reviewed the written description of HAH for clarity which informed the patient information sheet.

For peer review only - http://bmjopen.bmj.com/site/about/guidelines.xhtml

Results

Quotes which best presented the established constructs were identified by capital letters using scripts from patients (P), decliners (D), carers (C), consultants (Co), RSN (N) and managers (M). The Respiratory Specialist Registrar's comments were coded Co for anonymity. Quotations have been shortened; both the full quotation and additional supporting quotations are provided in the outline supplement. All patients that were interviewed survived to the end of the study.

Table 1: Description of participants

Participant role	n	Description
Patients participating	31	16 from UC, 15 from HAH. Mean (SD) age= 68 (10) years; 52% female; median (IQR) eMRCD score= 4 (3-4); mean (SD) FEV1= 43.4 (18.7) per cent predicted and previous admissions for an exacerbation= 0 (0-2).
Decliners	13	Mean (SD) age= 73 (11) years; 77% female; median (IQR) eMRCD score= 4 (4-4).
Carers	15	Mean (SD) age= 61 (11) years, 53% female, 80% were the patient's partner. 5 carers' relatives received UC and 10 carers' relatives received HAH.
RSNs	11	Mean (SD) age= 39 (11) years, 100% female, mean (SD) years' experience= 7 (5) years.
Physicians	15	11 respiratory consultants, 3 acute care physicians, and 1 specialist registrar; mean (SD) age= 41.5 (6) years, 71.4% were male, mean (SD) years reported experience = 12 (6)n years.
Managers	4	3 secondary and 1 social care; mean (SD) age= 50 (2) years, 50% were female.

RSNs, Respiratory specialist nurses; eMRCD, breathlessness score; UC, usual care; HAH, hospital at home; FEV1, forced expiratory volume.

Table 2: Social constructions of Hospital at Home

1. Positive Drivers for HAH

- 1.1 Availability of home comforts and maintaining independence
  - 1.1.1 Perception of a quicker recovery with HAH and positive effects on perceived breathlessness
  - 1.1.2 Improved sleep and nutrition
  - 1.1.3 More convenient place to be with friends and family
- 1.2 Confidence in the continuity of HAH care
  - 1.2.1 Feelings of safety, reassurance and appreciation
  - 1.2.2 Personalised relation and specialist expertise of the RSN

2. Potential Barriers and Negative Influences for HAH Care Pathway

- 2.1 Fear of being alone at home
- 2.2 Privacy issues and not wanting visitors
- 2.3 Resistance to change

For peer review only - http://bmjopen.bmj.com/site/about/guidelines.xhtml

2.3.1 Reluctance to accept removal of nebulised therapy
2.3.2 Challenging clinician's preconceptions, accepting a new model of care and operational concerns
2.4 Negative influences of HAH

3. Other Insights
3.1 Unintentional change in UC
3.2 Early uncertainty with HAH selected by DECAF

HAH, hospital at home; RSN, respiratory specialist nurse; UC, usual case; DECAF, prognostic score.

For peer review only

1. Positive Drivers for Hospital at Home

1.1 Availability of home comforts and maintaining independence

Patients, carers and RSNs, consistently highlighted that patients were comfortable in their home, with positive influences on mood and confidence. Hospital routines were avoided, and patients maintained their independence by engagement in usual activities. Patients requiring oxygen noted that the equipment provided within HAH allowed free movement, whilst provision in hospital limited mobility. *"I couldn't even walk up the ward… I only had this like little lead… I can go upstairs [at home]… it's nice and I can get up and make myself a meal" (P22L145-153).*

1.1.1 Perception of a quicker recovery with HAH and positive effects on perceived breathlessness

Patients and carers perceived that recovery occurred more quickly during HAH, and breathlessness was less marked, despite higher activity levels. This may reflect lower levels of anxiety within HAH: *"Just so relaxed… content with the care… because when you're anxious obviously it reacts on your breathing and your whole persona" (P6L109-112).* Compared to UC, delays in obtaining reliever therapy were also avoided within HAH.

1.1.2 Improved sleep and nutrition

Patients reported sleep disruption in UC related to disturbances from other patients, nurses obtaining regular observations and new admissions, whilst those who experienced HAH reported improved sleep: *"There's no bed like your own bed" (N19L129).*

Opinions were diverse concerning nutrition although most favoured HAH. *"(I) liked hospital food and ate more in hospital than at home" (P10L97)* and, *"You don't eat the same in hospital for a start" (P1L18).*

1.1.3 More convenient place to be with friends and family

Home is more convenient and saves time and money for family and friends. Travel, car parking, work absences, childcare issues, restrictive visiting hours and hospital TV costs are avoided. *"It's easier for*

my family to visit… getting cars parked… when they have been to work all day by the time they get the hospital it's time to come home" (P22L21-24).

A key benefit of HAH was increased time with grandchildren: "*When I'm in hospital I don't see the grandchildren. I've got six… and three of them live next door and they're here every single day. When I'm not here they do cry; they get really upset" (P13L167-169).*

Carers reported that providing care at home was less disruptive, by avoiding hospital visits which take more time and organisation: "*It's better my husband being at home… instead of… going back and forward to the hospitals and especially with being a carer looking after two children" (P17 L537-548).*

1.2 Confidence in the continuity of HAH care

1.2.1 Feelings of safety, reassurance and appreciation

Patients felt safe and reassured during HAH due to daily visits from the RSNs, the 24-hour telephone support line, confidence in the HAH clinical team, and the availability of emergency services if return to hospital was needed. The evening phone call (9pm) was reassuring to those patients living alone: *"I had confidence in the team that came out... they all seemed to me to be very well trained and putt me at ease" (P1L71-74),* and, *"If they thought you needed a doctor or the hospital they would phone an ambulance straight away" (P12L320-327).*

Most patients were not unduly concerned about potential delays in being seen by a doctor/clinician in the event of deterioration. *"If your hospital at home, if you take really poorly yes you phone up… they will still be here as quick as they would if you were in a hospital" (P12L319-327).*

Within HAH, patients appreciated the ability to take their medication at their usual time, with immediate access to reliever therapy.

1.2.2 Personalised relation and specialist expertise of the RSN

Patients in HAH appreciated the better continuity in healthcare professionals compared to inpatient care and described the relationship with RSN as "personal", "individual" and "one to one care". Clinical assessments conducted at home provided privacy and were less rushed with "time to develop rapport". Patients valued the clear explanations provided by RSNs during HAH about all aspects of their care, including monitoring, investigations, and management, commenting that this may be lacking in hospital.

Some patients experienced a lack of dignity and privacy during UC. *"A wash down behind the curtain, I was frightened I was rushing" (P32L53-55).* UC patients acknowledged time constraints as a barrier towards personalised care, which was also recognised by the RSNs.

Discharge from UC incurs delays due to waiting for medication, discharge letter and transport. Such concerns were not raised about transfer home under HAH or subsequent discharge.

For peer review only - http://bmjopen.bmj.com/site/about/guidelines.xhtml

2. Potential Barriers and Negative Influences for HAH Care Pathway

2.1 Fear of being alone at home

A common reason for declining participation in the RCT was fear of being alone when unwell, even when offered (same day) social support. The RSN stated that some patients were reluctant to accept social services because of perceived stigmatisation. *"The hours I would be on my own… say I got up to go the toilet and my legs went what do you do then if you can't breathe… you've got no phone at hand" (D1, P2L39-41)* and, *"They think if they've got a social worker or are involved with social services it's like they are labelled... they wouldn't realise they could just ring social services and ask for help" (N4L102-104).*

Participants recognised there may be a longer delay getting help if they deteriorated at home compared to in-hospital care. *"Some people maybe prefer to be in a cocoon of a hospital environment; they maybe worry if something goes wrong someone's seconds away from them" (P1L226-229).*

2.2 Privacy issues and not wanting visitors

Other decliners referred to privacy issues. "*I thought people coming to my house, I cannot… I'm frightened that it wouldn't be tidy*" (D3L4-7). For some, personal circumstances affected participation: *"My husband and I are divorcing and my house just having been sold the atmosphere at home wouldn't be conducive in recuperating… but in the future... I would very much like to be involved" (D9L14-21).*

2.3 Resistance to change

2.3.1 Reluctance to accept removal of nebulised therapy

During HAH patients received similar treatment to those within UC, including controlled oxygen therapy, nebulised bronchodilators and intravenous medications if required. Patients reported that nebulised bronchodilators helped chest clearance as well as relieving breathlessness. Patients in HAH were more likely to object to the removal of nebulised therapy than controlled oxygen. Similar objections were not raised to the same extent in UC. The challenge for the RSN was to reassure patients that such interventions were usually not required long term. *"I think a lot of them have the misconception that if they have a nebuliser they are better… Your inhalers are far better more practical" (N3L222-229).*

2.3.2 Challenging clinicians' preconceptions, accepting a new model of care and operational concerns

Physicians' preconceptions included the view that hospital care provides respite for carers and ensures patients feel safer. *"The patient themselves may not be ready for it… I think they need to stay in hospital where they feel safer not because it is safer but basically they feel safer and also it gives a break (to) their carer" (Co2 L53-66).* Another physician disagreed, stating: *"That's kind of often how people view patients with COPD that they want to come to hospital but actually it's not true" (Co3 L198-201).*

For peer review only - http://bmjopen.bmj.com/site/about/guidelines.xhtml

Operational concerns included keeping medical records in a patient's home and the inability to capture activity within current payment systems. *"Our work in trying to get the centre to change the way… we can record the data for hospital at home patients… perhaps by us doing that it would make it easier for other trusts to go down the hospital home route in the future" (M1L149-153)* and, *"If we are employing consultants, junior doctors, respiratory nurses, specialists nurses... and we are not having the patient in hospital so we are not getting the tariff for that… somebody has to pay the wage bill… and it's about understanding that payment mechanism… then that becomes a lot easier for everybody because that is the kind of thing that will stop people moving forward" (M17L183-193).*

A different model of care requires adjustment. The RSNs advised that their confidence increased with experience of delivering HAH, whilst patients mentioned that the quality of the service was dependent on the skill of the attending RSN. This highlights the importance of adequate training and support. *"The only concern I would have is that hospital at home is only as good as the nurses you've got on" (P12L336-337).*

2.4 Negative Influences of HAH

Patients felt less inclined to smoke in hospital. *"When I'm in hospital… I don't think about smoking whatsoever where… at home I do"" (P14L273-276).*

The hospital ward is an opportunity to establish new friendships that may continue post discharge and patients were concerned that their relatives might be frightened seeing/ knowing that they are unwell at home. *"I've made some nice friends… still ring them.. It was nice" (P22L497-505). "It's frightening for them to see when you're not well it's not nice for my husband to sit and watch us when I'm bad" (P25L220-222).*

3. Other Insights

3.1 Unintentional change in UC

Despite efforts to ensure UC was not influenced by trial participation, patients were discharged home earlier than expected. Factors include physician awareness of low risk of death by DECAF, pressure from patients who were disappointed with allocation to UC and pressure from operational service managers. "*There's much more emphasis on getting people home early… the people who we aren't (treating) in the hospital at home I think people are now more likely to send them home a lot earlier" (Co3 L120-126).*

3.2. Early uncertainty with HAH selected by DECAF

Initially some physicians expressed concern that, in their judgement, true risk for an individual patient was underestimated by DECAF. This led to delays in return home, which on occasion was unsettling for patients: *"They have certain reservations, so you've got that conflict between their clinical impression and the predictive score…the simple score almost always outperforms clinical judgement, but clinicians don't like to think that's true" (Co18L40-52).* With experience physicians became more confident using DECAF and the safety within HAH model of care: *"As you get evidence suggesting it works and it's safe you're more confident in doing it" (Co6L154-155).* This was reflected in the wider HAH population as delayed return home was primarily an issue at the start of the trial.

For peer review only - http://bmjopen.bmj.com/site/about/guidelines.xhtml

"I think part of the main advantage from my point of view is making me more aware of the safety of the DECAF score and the ability that people can be treated out of the hospital… whether that's because of the hospital at home trial or… because of the gradual change in practice because of the DECAF score I'm not sure" (Co6L99-103).

Carers

In the RCT, 23 carers in HAH and 18 carers in UC completed Zarit Burden Interviews, including 13 of 15 carers participating in this embedded qualitative study. Higher scores show higher levels of care burden. Baseline scores were similar in UC and HAH, but at 14 and 90 days scores were lower in HAH (table 3).

Table 3: Zarit Burden scores in carers

		Count, n	Median (IQR)	% improved or same v baseline	Missing
Zarit Burden 0 day	UC	18	18 (10-29)	Ref	1
	HAH	23	16 (10-30)	Ref	0
Zarit Burden 14 day	UC	18	23 (16-32)	35.7	3
	HAH	23	13 (8-31)	57.9	4
Zarit Burden 90 day	UC	18	25 (13-35)	16.7	5
	HAH	23	14.5 (10-36)	27.8	5

The percentage of patients with no change or an improvement in carer burden scores was higher in HAH, although this could be a chance finding.

Discussion

We interviewed 89 participants including patients, carers, and healthcare professionals to inform implementation of our HAH model. Whilst our RCT provides the justification for the implementation of HAH regarding cost and clinical outcome, this qualitative study identifies the human and organisational factors that will influence its successful implementation. Positive drivers included: greater independence and freedom; the maintenance of usual activities and perceived shorter recovery time; the maintenance of contact with friends and family (especially grandchildren); better sleep; and more time spent with an expert healthcare professional. Reported barriers from patients included being alone at night and having "strangers" visiting the home. Of note, some of these barriers, such as those relating to privacy and not wanting visitors, may be less of an issue at institutions which already have early supported discharge.

Early concerns from some physicians and RSNs diminished with experience of successful delivery of this model of care. Two patients that were allocated to HAH were kept in hospital at the consultant's discretion; this occurred in the first quarter of the study. Managers highlighted the inability for HAH activity to be captured within current payment systems. Hospitals planning to implement HAH selected by DECAF should pre-emptively address these issues. Compared to ESD, patients within HAH have higher clinical needs. Respiratory and Emergency Care Physicians require education, particularly highlighting the reliability of the DECAF score. RSNs delivering care at home require

For peer review only - http://bmjopen.bmj.com/site/about/guidelines.xhtml

additional clinical training, consultant support and capacity to deliver the service. The HAH Manual and assessment sheets used within our service are available to facilitate this process. Of note, training costs were included in the health economic analysis, which showed that this model of care is cost-effective. Maintaining clinical documents in a patient's home should be supported by local agreement and patient consent.

The study has several key strengths. We used a qualitative approach with semi-structured interviews to explore the issues around HAH for those involved in the service.[13] We performed a large number of interviews using purposeful sampling of patients to ensure both a wide range of participants and perspectives.[14] Not only were the experiences of patients and carers explored, but operational concerns from managers and clinicians were identified. Of note, we interviewed patients who declined involvement in the trial: this is a group rarely included in such studies. The study sites had no HAH service prior to the RCT, so the emergent themes relate to HAH as a new service. This supports the transferability of our results to other institutions which lack this service. Finally, the views of carers were triangulated with the Zarit Burden Interview.

The study has several limitations. As with all qualitative research, it is impossible to fully remove observer bias. To minimise this risk, interviews were performed by a female trained health psychologist who was separate from the clinical team and had no previous experience or knowledge in the design of the HAH care pathway, and three individuals independently performed analyses of the data to increase trust-worthiness and inter-rated reliability. Certain aspects, such as operational concerns, may only apply to the UK healthcare system. Whilst the inclusion of interviewed personal was large and broad, other members of the multidisciplinary team such as physiotherapists may have provided useful insights. Finally, there is a risk of social desirability bias, in that interviewees may tell us what they think we want to hear. This was minimised by the interviewer emphasising that they were not involved in clinical care and that frank discourse would help develop the service.

Previous RCTs of domiciliary treatment for patients with ECOPD had more exclusion criteria than our RCT, and mostly were of ESD rather than HAH.[15] Our RCT included patients typically deemed too unwell for home treatment in earlier studies, such as those with pneumonic ECOPD. Perceived risk remains a key factor for engagement, particularly during the early phase of service introduction, which will be important to address during educational sessions. In the DECAF study cohorts, 1,266 of 2,645 patients were DECAF 0-1 (low risk), and risk of death remained low regardless of the index scoring. In the HoT DECAF RCT, there were no acute deaths.[1] This is also in line with patient and carer perceptions in other trials. In a study of elderly patients, which included patients with ECOPD, substitutive HAH was reported as being safe by family and carers.[16] Similar findings were shown in a study of six patients with ECOPD.[17] In our study, patients and carers reported that HAH was safe, noting confidence in the clinical team and the availability of 24/ 7 telephone support, with return to hospital if required.

Five of thirteen patients who declined enrolment in the RCT reported fear of being home alone due to breathlessness, anxiety and/ or problems coping at home. Three patients regretted not enrolling, and three declined partly because of unwell family members in the home. Despite the high rates of pneumonia and co-morbidity, overall healthcare professionals were satisfied that the DECAF score selected low risk patients suitable for HAH. In the whole HAH population (n=60), there were only

For peer review only - http://bmjopen.bmj.com/site/about/guidelines.xhtml

three instances of discordance between clinician judgement and the DECAF score leading to patients randomised to HAH receiving UC, all of whom survived to 90 days follow up.

Carers of patients with COPD are at risk of various psychological health problems, such as anxiety and depression; partners of patients with COPD are typically elderly with their own health problems.[18] Utens and others looked at carer strain in an RCT of ESD compared to usual care, and showed no difference in the caregiver strain index between allocation groups.[19] Given the higher treatment needs of patients in HAH, we anticipated that the carers of patients treated with HAH would report increased carer burden. However, carers reported that inpatient stay was more disruptive than HAH, both in terms of time and cost. This was supported by the Zarit Burden Interview scores, which showed that the median carer burden scores were numerically lower with HAH than UC, though this could have been a chance finding. Whilst patients may express a fear of being or becoming a burden on their carers at the end of life,[20] this was not reported in any of the interviews with patients in the context of HAH for an acute exacerbation.

HAH selected by DECAF allows the inclusion of more patients than existing models, is preferred to inpatient care by most patients and their families, and is considered to be safe. HAH and the associated care was valued by many patients, in particularly with regards to continuity of care and in maintaining individuals' independence. Following this study, HAH has been commissioned as a service for all patients with low-risk ECOPD. The results of this study, both in terms of potential drivers and barriers, are important areas of discussion when offering HAH to patients.

For peer review only - http://bmjopen.bmj.com/site/about/guidelines.xhtml

Acknowledgements

This paper presents independent research funded by the National Institute for Health Research (NIHR) under its Research for Patient Benefit (RfPB) Programme (Grant Reference Number PB-PG-0213-30105). The views expressed are those of the authors and not necessarily those of the NHS, the NIHR or the Department of Health.

We thank Teresa Gibson for her input to the trial management group as an expert patient, the members of the Data Monitoring Committee (Robert Angus, Rodney Hughes, Nick S Hopkinson and Niall Anderson) and the Trial Steering Committee (Nicholas Hart, Jennifer Quint, Jadwiga Wedzicha, Patrick Murphy, Robert Rutherford). We thank Tracey Finch for her input in the initial draft of the project.

We are grateful to all interviewees for their time and candour.

Contributors

SCB conceived the study and was chief investigator with overall responsibility for the management of the study. SCB, AvW and CE were responsible for the original design and protocol, and obtaining funding. LLD and CE recruited patients to the study. LLD performed all interviews and transcripts. SCB, AvW, and LLD analysed all transcripts for themes. CE performed statistical analyses. LLD and AvW wrote the first draft of the manuscript, and all authors were involved in the final version.

Funding

NIHR Research for Patient Benefit and Northumbria Healthcare NHS Foundation Trust Teaching and Research Fellowship programmes.

Competing interests

All authors have completed a ICMJE uniform disclosure. SCB reports grants from NIHR: Research for Patient Benefit programme, during the conduct of the study; HTA funding, grants from Philips Respironics, grants from Pzifer Open Air, personal fees from Pzifer and AztraZeneca outside the submitted work. AvW reports grants from NIHR: Research for Patient Benefit programme, during the conduct of the study. LD, CE and GJG have no competing interests to declare.

Patient consent

Obtained

Ethics approval

Ethics approval was provided by NRES Committee North East Sunderland (3/NE/0275). All participants gave informed consent before taking part in the study.

For peer review only

Data Sharing Statement

Anonymised transcripts are available upon reasonable request; Lorelle.Dismore@northumbria-healthcare.nhs.uk. Additional supporting quotes for the identified themes can be found in the supplementary information.

For peer review only

For peer review only - http://bmjopen.bmj.com/site/about/guidelines.xhtml

References

1. Echevarria C, Gray J, Hartley T, et al. Home treatment of COPD exacerbation selected by DECAF score: a non-inferiority, randomised controlled trial and economic evaluation. *Thorax* 2018.
2. Steer J, Gibson J, Bourke SC. The DECAF Score: predicting hospital mortality in exacerbations of chronic obstructive pulmonary disease. *Thorax* 2012;67(11): 970-6.
3. Echevarria C, Heslop-Marshall K, Stenton SC, et al. Validation of the DECAF score to predict hospital mortality in acute exacerbations of COPD. *Thorax* 2016; 71(2): 133-40.
4. British Thoracic Society. The burden of lung disease: a statistical report from the British Thoracic Society, 2nd ed. London: British Thoracic Society, 2006.. https://www.brit-thoracic.org.uk/document-library/delivery-of-respiratory-care/burden-of-lung-disease/burden-of-lung-disease-2006/
5. Seemungal TA, Donaldson GC, Paul EA, Bestall JC, Jeffries DJ, Wedzicha JA. Effect of exacerbation on quality of life in patients with chronic obstructive pulmonary disease. *Am J Respir Crit Care Med* 1998; 157(5): 1418-22.
6. Vestbo J, Hurd SS, Agusti AG, et al. Global strategy for the diagnosis, management, and prevention of chronic obstructive pulmonary disease: GOLD executive summary. *Am J Respir Crit Care Med* 2013; **187**(4): 347-65.
7. National Institute for Health and Care Excellence. *Chronic obstructive pulmonary disease in over 16s: diagnosis and management.* England: NICE; 2010.
8. NHS England, Public Health England, Health Education England, Monitor, Care Quality Commission, NHS Trust Development Authority Five Year Forward View 2014. https://www.england.nhs.uk/wp-content/uploads/2014/10/5yfv-web.pdf
9. Campbell M, Fitzpatrick R, Haines A, et al. Framework for design and evaluation of complex interventions to improve health. *BMJ.* 2000; 321(7262): 694-6.
10. Bradley F, Wiles R, Kinmonth AL, Mant D, Gantley M. Development and evaluation of complex interventions in health services research: case study of the Southampton heart integrated care project (SHIP). *BMJ.* 1999; 318(7185): 711-5.
11. Dismore L, van Wersch, A, Swainston K. Social constructions of the male contraception pill: When are we going to break the vicious circle? *J Health Psychol* 2016; **21**(5): 788-97.
12. Shepperd S, Lliffe S. Hospital-at-home versus in-patient hospital care. *Cochrane Database Syst Rev.* 2005(3).
13. Greenhalgh T, Taylor R. How to read a paper: papers that go beyond numbers (qualitative research). *BMJ* 1997; 315(7110): 740-3.
14. Bryman A. Social Research Methods: Oxford University Press, 2008.
15. Echevarria C, Brewin K, Horobin H, et al. Early Supported Discharge/ Hospital At Home For Acute Exacerbation Of Chronic Obstructive Pulmonary Disease: A Review and Meta-Analysis. *COPD Journal.* 2016; 13(4): 523-33.
16. Leff B, Burton L, Mader SL, et al. Comparison of stress experienced by family members of patients treated in hospital at home with that of those receiving traditional acute hospital care. *J Am Geriatr Soc* 2008; 56(1): 117-23.
17. Wang Y, Torbjorn H, Steihaug S. & Werner A. Patients with acute exacerbation of chronic obstructive pulmonary disease feel safe when treated at home: a qualitative study. *BMC Pulm Med* 2012; 12(1): 45.
18. Gardiner C, Gott M, & Small N. Living with advanced chronic obstructive pulmonary disease: patients concerns regarding death and dying. *Palliat Med* 2009; 23: 691-7.
19. Utens CM, van Schayck OC, Goossens LM, et al. Informal caregiver strain, preference and satisfaction in hospital-at-home and usual hospital care for COPD exacerbations: results of a randomised controlled trial. *Int J Nurs Stud* 2014; 51(8): 1093-102.

For peer review only - http://bmjopen.bmj.com/site/about/guidelines.xhtml

20. Jones I, Kirby A, Ormiston P, et al. The needs of patients dying of chronic obstructive pulmonary disease in the community. *Fam Pract* 2004; 21(3): 310-3.

For peer review only

Table of themes with quotations

Online supplementary report of full and additional quotations related to themes. Interviewer quotes are shown in bold.

Theme 1. Positive Drivers for HAH	Quotes
1.1 Availability of home comforts and maintaining independence	I couldn't even walk up the ward or anything you know because I only had this like little lead you know and I used to keep forgotten walking off then if you wanted to talk to anybody you had to either shout or they had to come to you you know but that's the only thing just sort of sat there all the time (P22L145-148)<br><br>**And now in the home how would you describe this situation with the oxygen lead?**<br><br>Oh yes I can go upstairs do different pieces up there you know tidy my draws up and that's you know just a couple at a time just err no its nice and I can get up and make myself a meal you know (P22L145-153)<br><br>Yeah well what can you do in hospital you're either lying on a bed or sitting in a chair I can walk back and forward in here you know the and I can go upstairs and down bath wash shave that type of thing you know were as well you can do that in hospital get washed and shaved and that but you're stuck in the one place you're not going anywhere you know you're not getting no exercise nothing (P18L114-118)<br><br>In a lot of ways you're disempowering the patient by keeping them on a ward and keeping them within that sort of very tight sort of role, when you get them home then they're they're dressed, they are doing more things themselves (N7L293-296)
1.1.1 Perception of a quicker recovery during HAH and positive effects on perceived breathlessness	Her recovery time was much quicker so it was easier for me because she was helping herself do you know what I mean (CP28L260-261)<br><br>Just so relaxed content, content with the care everything just made everything because when your anxious obviously it reacts on your breathing and your whole persona really and just everything was so relaxed I don't know how else to describe it (P6L109-112)
1.1.2 Improved sleep and nutrition	People recover better in their own home and there was one lady and I quote her often to the staff there's no bed like your own bed and that stuck because actually she was right absolutely right you know why be sitting in

For peer review only

	a hospital bed when you've got poorly people around you if you have that support to be able to recuperate at home (M19L128-132).<br><br>I was very surprised at the food it wasn't too bad. In fact I had more in the hospital than I did at home (P10L92-97)<br><br>You don't eat the same in hospital for a start and you certainly don't sleep the same you can be awake most of the night (P6L91-92)
1.1.3 More convenient place to be with friends and family	It's easier for my family to visit and everything you know getting cars parked you know when they have been to work all day by the time they get the hospital it's time to come home you know so it's definitely benefitted me you know (P22 L21-24)<br><br>But even the grand children when I'm in hospital I don't see the grandchildren I've got six grandchildren and three of them live next door and they're here every single day when I'm not here they do cry they get really upset (P13 L167-169)<br><br>They don't like fetching them in when they're so young I mean the youngest ones four and the twins and they miss us when I'm not here (P16L87-88)<br><br>It's better my husband being at home and getting the treatment at home instead of like going back and forward to the hospitals and especially with being a carer looking after two children as well you know it's a lot better<br><br>**So when you say you look after two children as well, so are you a carer for those two children and Jim?**<br><br>Yes<br><br>**So hospital at home how does that make it easier in regards to that?**<br><br>Like with looking after Jim and getting the support and like for visit and things you know like when he's at home he's at home and getting looked after at home and I can think of the children and it doesn't put as much

For peer review only

	pressure on us as well because like when you're visiting and you've got children to pick up at school as well you know it's all like the time as well (P23 L537-548)<br><br>It's been much better for me anyway because I used to get knackered go into hospital every day and coming back then having to go back again and coming back whereas been here I can go to work come in do whatever I wanted to do when I knew she was safe she was all right in the house do you know what I mean (P28L225-228)<br><br>Financial yes yes because you see were on a low income my partner only works part time window cleaner and we have to pay full rent and everything and money is really tight and for him to keep coming back and forth to hospital I mean sometime if there's no money he can't go you know so that's the benefits because he even though he's a part time window cleaner he can still go to work when I'm poorly he works local (P13L56-61)<br><br>At first I was a bit concerned because I look after the grandchildren not every day four days a week sometimes erm and I was more concerned about them whether it would interfere with personal life sort of thing err and then went and thought about it it obviously wouldn't I'd get to see them more than what I would actually been in hospital so that was the deciding factor really (P6L12-16)
1.2 Confidence in the continuity of HAH care<br>1.2.1 Feelings of safety, reassurance and appreciation	I had confidence in the team that came out I think I saw four different people over a short period of time they all seemed to me to be very well trained and putting me at ease and I really didn't feel like I was missing anything that I would have had in hospital you know (P1L70-73)<br><br>Now I feel what's the word more secure I don't know yeah more secure because I know all I've got to do is make a phone call like obviously there's name of doctor there's obviously the nurses if I have to there would be you there is more people to talk to now and that's something I never had before which is good' (P13L103-106)<br><br>Few have said but are you not nervous X like if you're in a hospital you're on a ward they get a doctor to you as soon as they can and they might not get it as soon as you can might be two three hours down the line but if your hospital at home if you take really poorly yes you phone up you've got that number they will still be here as quick as they would if you were in a hospital do you know what I mean it doesn't mean to say because you're on a ward that a nurse or a doctors going to get to you do you know and I would phone a the district nurse or community nurse and if they thought you needed a doctor or the hospital they would phone an ambulance straight away (P12L321-328)

For peer review only

	Telephone call before I came out I got a telephone call before the afternoon in the evening check that things were okay I was told on that evening who would be coming the next day so I knew who to expect so I don't have a problem at all with it (P1 P7 L320-323)
1.2.2 Personalised relation and specialist expertise of the RSN	A wash down behind the curtain I was frightening I was rushing (P32L53-55)<br><br>I mean you cannot get individual care can you really because there's 16 on the ward another five people in care and of course at home you're getting the care more attention (P16 L163-165)<br><br>They checked everything every day you know everything ankles everything whereas a rule it's just your blood pressure and your oxygen level in the hospital and your erm temperature yes but I don't know they were just really brilliant they were here quite a long time you know making sure I was feeling all right and I was confident on my own you know but no I found them really good and there are so nice you know really nice and caring (P22L44-49)<br><br>I'll be perfectly honest the times I've been in the hospital the nurses there been too busy or there's only been one on or two on and they can't do anything you know there to busy doing other things you don't get a one to one erm but in your home you do get one to one (P12L208-211)<br><br>She made me feel safe she made me feel I was getting a proper treatment erm comfortable is the word she made me feel really comfortable knew what she was doing explained everything even down to when you were getting your stats done you know when you were getting while you were getting your bloods taken erm explained everything you don't always get that in a hospital (P12L365-370)<br><br>You get told you can get out tomorrow and then they say you've got tablets and everything and by the time its tea time you're still waiting there somebody coming to get you or getting the tablets or getting the letter but everything's so well done there was didn't get you harassed at all and it just seemed like you go home today you've got your tablets you've got your letter you were taken down to the departure place the nurse was there they had been with the oxygen so well planned it was like err just sail a long and I like that because it didn't get as well erm and it made you feel more relaxed and thinking to yourself well if this is how it's going to be then that's okay I can cope (P20L423-431)
2. Potential Barriers and	

For peer review only

Negative Influences of HAH Care Pathway	
2.1 Fear of being alone at home	The hours I would be on my own you know if just say I got up to go the toilet and my legs went what do you do then if you can't breathe you know you've got no phone at hand you know you cannot pull an alarm switch or anything so no (Decliner 1 P2L39-41)<br><br>Maybe it's the generation thing I mean mention a social working oh no I haven't got one of them but I mean you know I think they think if there've got a social worker or they're involved with social services it's like there labelled but it's not that but they don't realise that so you know until its explained to them so there would be sat at home and they wouldn't realise they could just ring social services and ask for help (N4L100-104)<br><br>Some people maybe prefer to be in a cocoon of a hospital environment, they maybe worry if something goes wrong someone's seconds away from them (P1L226-229)
2.2 Privacy issues and not wanting visitors	I thought people coming to my house, I cannot I'm frightened that it wouldn't be tidy and things like that, that's what type of person I am I'm frightened that it wouldn't be tidy (D3L4-7)<br><br>My husband and I are divorcing and my house just having been sold the atmosphere at home wouldn't be conducive in recuperating...but in the future ... I would very much like to be involved (D9L14-21).
2.3 Resistance to change<br>2.3.1 Reluctance of the removal of nebulised therapy	The nebulisers seem to clear you and they clear you quickly and they seem as if erm you get relief after you've had them (P12L90-91)<br><br>I mean I take all my self-medication at home anyway but you know I'm on steroids and antibiotics and so I've got them at home anyway as I say it's just if I had a nebuliser at home that would sort of cure everything I think (P7L57-59)<br><br>I would still rather have home treatment you know if I had a nebuliser I would be happy as Larry I would be because once I'm on that and take a couple of doses I'm fine (P7L69-71)<br><br>I feel safer actually with that there (P27L311-313)

For peer review only

	I think a lot of them have the misconception that if they have a nebuliser they're better and that seems to stick in their mind so of course then we have to go down do you realise it increased your heart rate its higher mortality rate ideally it's for emergency you shouldn't be having them all the time so we do sort of stress to them you don't want to be on this treatment your inhalers are far better more practical more easier to use so we do try our best to get them off (N3L223-229)
2.3.2 Challenging clinicians' preconceptions, accepting a new model of care and operational concerns	The patient themselves may not be ready for it I may be ready for it but the patient may be two days away from it and also sometimes the patient needs would like if someone came into a hotel for their illness for a while they want to be away from their sick bed because their home is almost like a toxic area so all I'm doing is what I'm trying to tell the patient is I can actually bring the hospital to your home with some patients it's a good idea but some patients say actually I want to take a break from my home<br><br>**So is that the major difference in those patients who want to stay in hospital?**<br><br>Yeah because I think they need to stay in hospital where they feel safer not because it is safer but basically they feel safer and also it gives a break their carers would like to get rid of them for two or three days there's many different things going on in the patient the mind of the patient<br>(Co2 P2L53-66)<br><br>That's kind of often how people view patients with COPD that they want to come to hospital but actually it's not true (Co3 L198-201)<br><br>Our work in trying to get the centre to change the way erm we can record the data for hospital at home patients erm perhaps by us doing that it would make it easier for other trusts to go down the hospital home route in the future (M1P4 L151-153)<br><br>If we're employing consultants junior doctors respiratory nurses specialists nurses and health cares we're not having the patient in hospital so we're not getting the tariff for that but we're sending those patients out to look after them somebody has to pay the wage bill for that and it's about understanding that payment mechanism and if that's absolutely crystal clear then that becomes a lot easier for everybody because that's the kind of thing that will stop people moving forward (M17L188-193)

For peer review only

	Think the only concern I would have is that hospital at home is only as good as the nurses you've got on (P12L336-337)
2.4 Negative Influences of HAH	I mean for all I'm a smoker when I'm in hospital, things like that, I don't think about smoking whatsoever where I am at home I do but erm whenever I'm in a strange place like that I never smoking never crosses my mind (P14L273-276).<br><br>I've made some nice friends when I was in for a week you know still ring them you know erm but that was nice but I didn't wander round and talk to them you know but erm as I say erm the first time I went in it was nice (P22L497-499)<br><br>It's frightening for them to see when you're not well it's not nice for my husband to sit and watch us when I'm bad (P25L220-222)
3. Other Insights<br>3.1 Unintentional change in UC	There's a much more emphasis on getting people home early and I think even the people who are in the usual care arm so the people who we aren't doing in the hospital at home I think people are now more likely to send them home a lot earlier than they did when I did my stuff four years ago so I think general practice has changed we're not hanging on to these patients as long as we did erm and that's through external pressures erm but you know external pressures make you want to get people home as quickly as possible but the trial itself might be running the trial might also be changing peoples practice and may be making them send people home earlier (Co3 L121-129)
3.2 Early uncertainty with HAH selected by DECAF	They have certain reservations so you've got that conflict between there clinical impression and the predictive score. There are patients where as I said their risk is low but the clinical judgement is that they are not low risk now the difficulty here is that we shouldn't necessarily ignore our clinician judgement because well it's important and it's important to listen to however again and again the studies show that clinical judgement is not as good as prognostic scores so you've got research from the 1950s and literally hundreds of studies now from medicine and kind of baseball wine-making you name it completely different types of erm different types of expertise the simple score almost always out performs clinical judgement but clinicians don't like to think that's true (Co18L40-52).<br><br>For some cases it seems obviously a good thing to do then there's other cases where previously you might have

For peer review only

	thought better keep those inpatients in for a few days rather than sending them home so a couple of patients were that's realise that's different from what you normally do and you do think not anxiety but you do think oh I hope that things are going to alright but that's why we're doing a trial because we don't know (Co3L22-27)<br><br>As you see it works and as you get evidence suggesting it works and its safe you're more confident in doing it (Co6L154-155)<br><br>I think part of the main advantage from my point of view is making me more aware of the safety of the DECAF score and the ability that people can be treated out of the hospital so more whether that's because of the hospital at home trial or whether that's just because of the gradual change in practice because of the DECAF score I'm not sure (Co6L99-103)

For peer review only

Interview questions – Carers

Background to the carer

1) Can you describe your relationship to (X)?
2) Can you describe the care you give at home?
3) What sort of care do you provide?
4) Can you describe a typical day/how much of your day does it take?
5) How has your life changed since you have been providing care?
    - Has it affected your daily routine (what was it like before/how is it now – how would you describe what you do – how do you feel)?
    - What's good or bad about it?
    - Is it becoming easier/harder – in what ways?
    - What do you find hardest about caring for someone? (loneliness/fear/isolation/financial difficulties)
6) How has care changed over time?
7) Is there a change when your relative's health suddenly worsens?
8) What helps you in providing care?
    - (coping/support/faith/family)
    - Do you receive any help/support from anyone?
10) How do you feel about providing care? (burden/guilty/helpless)
11) What do you think about someone from a social services team coming in to help provide care?
12) What does the word carer mean to you/how do you feel about the word carer?

Project Questions

1) What are your perceptions of hospitals?
2) Has your relative been previously hospitalised?
3) Can you describe past experiences of hospital?
4) How do you feel if your relative has to go into hospital?
5) Can you describe a typical day on which your relative is hospitalised – what type of care do you provide?
6) Can you tell me what your initial thoughts were when you were told about HAH?
7) Initially your reasons for preferring home treatment were (QUOTE). Can you explain this in more detail?
8) How did you find (mode of treatment)?
    - Expectations of (mode of treatment)/different from your experience?
9) How was it being at (mode of treatment) with your relative?
10) How would you have felt if (X) was treated (mode of treatment)?
11) Can you describe what happened whilst (X) was being treated (mode of treatment)?
    - What did they do whilst at home? Relaxed or not?
12) What is your preference now following your recent experience?
13) If in the future HAH is available, would you choose to have your relative treated at home or in hospital? Why?

For peer review only

14) Can you describe the care you give your relative comparing when at home and hospitalised? – any differences?
15) Where would you prefer to care for your relative?
16) How does it benefit you?
17) Any disadvantages of having to care for your relative at (mode of treatment)?
18) What concerns would you have in the future if your relative is treated at (mode of treatment)?

Dr-patient Relationship

1) Can you describe your experience of the interaction between yourself and the doctors/nurses or others you have seen?
2) Can you describe the role of the nurse/doctor?
3) Can you describe the relationship you had with the nurse?
    - What do you talk about with the nurses – what do they say to you?
4) In what ways would the relationship differ between a nurse visiting your relative at home compared to on a ward?
5) Home/respiratory specialist nurse, what do you think about this?
6) Did you understand what was required from you? Concerns?
7) Anything missing/any disadvantages?
8) What would have made it better for you?
9) What went particularly well or what were the main benefits for you?

Nebulisers

1) What do you understand about nebulisers/inhalers?
2) Can you describe a situation in which your relative has needed to use one - in (mode of treatment) - What's the difference in using one at home versus in hospital?
3) Why would your relative use a nebuliser/inhaler, what would trigger its usage, anything in particular?
4) Is there any issue of using one at home? Are you using it more often/over using it/is it better at home?
5) How often do you find they use a nebuliser/inhaler at home?
6) What are the advantages/disadvantages of nebulisers/inhalers?

Summary question

1) Anything else you would like to tell me or anything else you think I should know?

Interview questions – Health care professionals (clinician interviews)

1) Can you begin by telling me what you understand about hospital at home (HAH)?
2) Would you be able to share your views of HAH – What do you feel about home treatment?
3) How did you become involved in the project and how long for/would you like more involvement?
4) Can you describe your role? And what affect if any the project has on it?
5) Have you been involved in the treatment of patients at home/in hospital – can you describe this?

For peer review only

6) What do you think about when considering if a patient is eligible for HAH?
7) Have you had any patients that you haven't put through to the trial/any situation where you would not recommend a patient for HAH?
8) What's the difference in care if any a patient receives in HAH?
9) Organisation wise how do you feel the project is running?
10) What were your expectations of HAH? Have these changed?
11) Is there anything that is going particularly well about it or what are the main benefits you can see?
12) Do you see any disadvantages or can you recommend an area of improvement?
13) Do you have any preferences as to where to treat patients?
14) What do you think about the future implementation?
15) Is there anything else you think I should know or anything you would like to tell me?

Interview schedule for managers

1) Can you begin by telling me what you understand about hospital at home (HAH)?
2) Would you be able to share your views of hospital at home – how do you feel about home treatment as a service for patients?
3) With regard to the current RCT of HAH for patients with an exacerbation of COPD, can you describe your role and your involvement in the trial?
4) From the perspective of this Trust, how do you think the trial is running?
5) What are your views on using DECAF to identify low risk patients?
6) Is there any conflict between clinician judgement and DECAF?
7) What were your expectations of HAH? Have these changed?
8) Can you recommend an area of improvement?
9) What are the advantages from a patients'/clinicians' perspective?
10) What are the disadvantages from a patients'/clinicians' perspective?
11) Is there anything that is going particularly well about it or what are the main benefits you can see?
12) Can you describe the role of a respiratory nurse?
13) Can you describe the interaction between the respiratory nurses and consultants?
14) What are the advantages, and challenges, from the perspective of a hospital manager or director/ hospital information services and management?
15) Have there been any issues with the trial?
16) How do we establish and support hospital at home as a clinical service?
17) What support do you think the clinical staff need to effectively implement the service?
18) Should it be possible, if the trial results are positive and no major issues are experienced, that this could be used as a model to establish and implement similar services in other trusts?
19) Are there any barriers towards implementation? If so, how do you think these should be tackled?
20) What do you think of it being a seven day service?
21) If other trusts are to implement the trial how do we support this?
22) What level of central engagement is needed if the trial is successful to make it easier for other trusts?

For peer review only

23) Anything else you would like to discuss?

Interview Questions for patients

Experience

1) Can you describe your experience of how you came to be in hospital?

Project information

1) Can you tell me your initial thoughts when asked to participate in the study?
2) Initially you stated a preference for (mode of treatment). Has your preference changed following your recent experience?
3) Initially your reasons for preferring (mode of treatment) were (quote) can you explain this in more detail?
4) How did you find (mode of treatment)?
5) If in the future HAH is routinely available, would you choose to have treatment at home or in hospital? Why?
6) What were your expectations previously and was your experience different from these?
7) How did you feel when at (mode of treatment)?
8) How would you have felt if treated (mode of treatment)?
9) How do you feel when the nurse or doctor comes to visit you at (mode of treatment)/would this differ if you were (in other mode of treatment)?
10) If any of your friends had any problems would you suggest to them to go for HAH?
11) Would there be circumstances with your friends where you think, "This would not suit this person but it suits me?"
12) Did you ever have to ring the nurse whilst at home – what was your experience?
13) Can you describe the role of the doctors/nurses or others you have seen?
    - Did you understand what was required from you? Concerns?
    - Anything missing/any disadvantages?
    - What would have made it better for you?
    - What went particularly well or what were the main benefits for you?

Nebulisers

1) Can you describe a situation in which you have needed to use a nebuliser? - Hospital/at home?
2) What's the difference between using one at home versus in hospital?
3) Why would you use one at home/what would trigger its usage, anything in particular?
4) (at home) Are you using it more often/over using it/how do you find using it/how often?
5) Any situation where you would prefer not to use one? Why?
6) What are the benefits/disadvantages?
7) Summarise - Is there anything else you would like to tell me?

Interviews Nurses

1) Can you begin by telling me what you understand about hospital at home (HAH)?
2) Would you be able to share your views of HAH – What do you feel about home treatment?

For peer review only

3) How did you become involved in the project – what's you role?
4) What affect does the project have on your role as a nurse – how has it changed your role?
5) Are you happy with your involvement would you like more/less?

Treatment

1) Have you been involved in the treatment of patients at home– can you describe this?
2) What's it like visiting patients in their homes/how do you feel when there?
3) How does this differ to treating someone in hospital? What's the main difference to you?
4) Have you ever done supported discharge visits?
5) How does HAH compare to this – similarities/differences?
6) Is there any anxiety over one or the other – why?
7) How do you feel making decisions for patient's treatment?
8) Have you had a situation where it has not benefited the patient or can you see were this may happen?

Nebulisers

1) Is there a difference in nebuliser treatment in patients at HAH versus UC?
2) Is there a difference in how patients use them? Why?
3) Is there any reluctance to take the nebuliser away from those at home? Difference in how you use them?
4) How often do patients use them at home? What happens if there not used?
5) Any issues with nebulisers that you can see?
6) Do you have a preference as to why you prefer to treat patients?

The project

1) Organisation wise how do you feel the project is running?
2) How has it changed normal procedures – what's now different about it?
3) Is there anything that is different about it that people may not necessarily agree on?
4) Are there any issues with the HAH patients having their clinical notes?
5) What were your expectations of HAH? Have these changed?
6) Is there anything that is going particularly well about it or what are the main benefits you can see?
7) Do you see any disadvantages or can you recommend an area of improvement?
8) How do you feel over all – pleasure/burden? Concerns?
9) What do you think about the future implementation?
10) What are your views on the on call respiratory physician?
11) How has this impacted?

Relationships

1) Can you describe the relationship between yourself and the patients when in a patient's home?
2) What do you speak to patients about?
3) What do they tell you?
4) Can you describe the relationship with patients when seeing them on the ward?
5) What's the relationship like with patients at home/compared to hospital?
6) Is there a difference in relationships between men and women?

For peer review only

7) Ever feel intimidated or are you ever more careful?
8) What do you feel is important for a good working relationship with patients?

Hospital A

1) What are you views on Hospital A?
2) How has Hospital A impacted the process of HAH? Easier/harder?

Anything else?

For peer review only

COREQ (COnsolidated criteria for REporting Qualitative research) Checklist

A checklist of items that should be included in reports of qualitative research. You must report the page number in your manuscript where you consider each of the items listed in this checklist. If you have not included this information, either revise your manuscript accordingly before submitting or note N/A.

Topic	Item No.	Guide Questions/Description	Reported on Page No.
**Domain 1: Research team and reflexivity**			
Personal characteristics			
Interviewer/facilitator	1	Which author/s conducted the interview or focus group?	10
Credentials	2	What were the researcher's credentials? E.g. PhD, MD	10
Occupation	3	What was their occupation at the time of the study?	10
Gender	4	Was the researcher male or female?	10
Experience and training	5	What experience or training did the researcher have?	10
Relationship with participants			
Relationship established	6	Was a relationship established prior to study commencement?	NO
Participant knowledge of the interviewer	7	What did the participants know about the researcher? e.g. personal goals, reasons for doing the research	
Interviewer characteristics	8	What characteristics were reported about the inter viewer/facilitator? e.g. Bias, assumptions, reasons and interests in the research topic	10
**Domain 2: Study design**			
Theoretical framework			
Methodological orientation and Theory	9	What methodological orientation was stated to underpin the study? e.g. grounded theory, discourse analysis, ethnography, phenomenology, content analysis	4
Participant selection			
Sampling	10	How were participants selected? e.g. purposive, convenience, consecutive, snowball	3
Method of approach	11	How were participants approached? e.g. face-to-face, telephone, mail, email	3
Sample size	12	How many participants were in the study?	3-4
Non-participation	13	How many people refused to participate or dropped out? Reasons?	NONE
Setting			
Setting of data collection	14	Where was the data collected? e.g. home, clinic, workplace	3
Presence of non-participants	15	Was anyone else present besides the participants and researchers?	NO
Description of sample	16	What are the important characteristics of the sample? e.g. demographic data, date	4
Data collection			
Interview guide	17	Were questions, prompts, guides provided by the authors? Was it pilot tested?	3
Repeat interviews	18	Were repeat inter views carried out? If yes, how many?	4
Audio/visual recording	19	Did the research use audio or visual recording to collect the data?	4
Field notes	20	Were field notes made during and/or after the inter view or focus group?	
Duration	21	What was the duration of the inter views or focus group?	30-60
Data saturation	22	Was data saturation discussed?	3
Transcripts returned	23	Were transcripts returned to participants for comment and/or	

Topic	Item No.	Guide Questions/Description	Reported on Page No.
		correction?	4
**Domain 3: analysis and findings**			
Data analysis			
Number of data coders	24	How many data coders coded the data?	10
Description of the coding tree	25	Did authors provide a description of the coding tree?	
Derivation of themes	26	Were themes identified in advance or derived from the data?	4
Software	27	What software, if applicable, was used to manage the data?	[illegible]
Participant checking	28	Did participants provide feedback on the findings?	
Reporting			
Quotations presented	29	Were participant quotations presented to illustrate the themes/findings? Was each quotation identified? e.g. participant number	5 – 9
Data and findings consistent	30	Was there consistency between the data presented and the findings?	
Clarity of major themes	31	Were major themes clearly presented in the findings?	5 – 9
Clarity of minor themes	32	Is there a description of diverse cases or discussion of minor themes?	6, 7, 8,

Developed from: Tong A, Sainsbury P, Craig J. Consolidated criteria for reporting qualitative research (COREQ): a 32-item checklist for interviews and focus groups. *International Journal for Quality in Health Care*. 2007. Volume 19, Number 6: pp. 349 – 357

Once you have completed this checklist, please save a copy and upload it as part of your submission. DO NOT include this checklist as part of the main manuscript document. It must be uploaded as a separate file.

BMJ Open

What are the positive drivers and potential barriers to implementation of hospital at home selected by low risk DECAF score in the UK, a qualitative study embedded within a randomised controlled trial.

Journal:	*BMJ Open*
Manuscript ID	bmjopen-2018-026609.R2
Article Type:	Research
Date Submitted by the Author:	20-Feb-2019
Complete List of Authors:	Dismore, Lorelle; Northumbria Healthcare NHS Foundation Trust, Research and Development; Teesside University, School of Social Sciences, Humanities and Law<br>Echevarria, Carlos; Northumbria Healthcare NHS Foundation Trust, Respiratory<br>van Wersch, Anna; Teesside University, School of Social Sciences, Humanities and Law<br>Gibson, John; Newcastle university, Institute of Cellular Medicine<br>Bourke, Stephen; Northumbria Healthcare NHS Foundation Trust, Respiratory
**Primary Subject Heading**:	Qualitative research
Secondary Subject Heading:	Respiratory medicine
Keywords:	QUALITATIVE RESEARCH, Quality in health care < HEALTH SERVICES ADMINISTRATION & MANAGEMENT, Protocols & guidelines < HEALTH SERVICES ADMINISTRATION & MANAGEMENT, Respiratory infections < THORACIC MEDICINE, qualitative, COPD, exacerbation, service development

SCHOLARONE™
Manuscripts

What are the positive drivers and potential barriers to implementation of hospital at home selected by low risk DECAF score in the UK, a qualitative study embedded within a randomised controlled trial.

Authors

Corresponding author and lead author

Lorelle Louise Dismore[1]

Department of Research and Development, North Tyneside General Hospital, Rake Lane, North Shields, Tyne and Wear NE29 8NH, UK, 0191 293 4087;

lorelle_dismore@hotmail.com

Additional authors

Carlos Echevarria,[1,3] Anna van Wersch,[2] John Gibson,[3] Stephen Bourke[1,3]

[1] Northumbria Healthcare NHS Foundation Trust, Tyne and Wear, UK, [2]Teesside University, Middlesbrough, UK, [3]Newcastle University, Newcastle upon Tyne, UK.

Key words; qualitative, COPD, exacerbation, service development

Word count: 4402

For peer review only - http://bmjopen.bmj.com/site/about/guidelines.xhtml

Abstract

Objective

Hospital at home (HAH) for COPD exacerbation selected by low-risk DECAF score is clinically and cost effective; DECAF is a prognostic score indicating risk of mortality. Up to 50% of admitted patients are suitable; a much larger proportion than earlier services. Introduction of new models of care is challenging, but may be facilitated by informed engagement with stakeholders. This qualitative study sought to identify facilitators and barriers to implementation of HAH.

Design

Semi-structured interviews, data were analysed using Thematic-Construct Analysis.

Setting

Interviews were conducted within patients' homes and hospitals in North East England.

Participants

Eighty-nine participants were interviews; 44 patients, 15 carers, 15 physicians, 11 specialist nurses and 4 managers.

Results

Facilitators include: 1) availability of home comforts and maintaining independence (with positive influences on perceived rate of recovery, sleep quality and convenience for friends, family and carers); and 2) confidence in the continuity of HAH care.

Barriers include: 1) fear of being alone at home; 2) privacy issues and not wanting visitors; and 3) resistance to change. Clinician concerns occasionally delayed return home, principally during the early phase of the trial. Nurses cited higher workload and greater responsibility, but with additional resource and training overall they viewed HAH positively. Operational concerns included keeping medical records in a patient's home and inability to capture activity within current payment systems.

Conclusion

HAH selected by DECAF was preferred to inpatient care by most patients and their families. Implementation in other hospitals will require education, training and service planning, tailored to overcome the identified barriers.

ISRCTN29082260.

Strengths and limitations

- A large number of interviews were performed using purposeful sampling to ensure a wide range of participants and perspectives.
- Member checking was performed on physicians.
- Interviews were conducted by a health psychologist separate to the clinical team to minimise risk of observer bias.

For peer review only - http://bmjopen.bmj.com/site/about/guidelines.xhtml

For peer review only

- There is a risk of social desirability bias with interviewees telling us that which we wish to hear.
- The study was conducted within a UK healthcare setting and may not be representative to other countries implementing a HAH care pathway.

For peer review only

For peer review only - http://bmjopen.bmj.com/site/about/guidelines.xhtml

Introduction

In a randomised controlled trial (RCT) we have shown that Hospital at Home (HAH) selected by the DECAF prognostic score is cost-effective, safe, and preferred by 90% of patients.[1] DECAF is a prognostic score that can be used to assess the inpatient risk of death in those with a Chronic Obstructive Pulmonary Disease exacerbation (ECOPD) using indices that are routinely available at admission: Dyspnoea, Eosinopenia, Consolidation, Acidaemia and atrial Fibrillation. It offers excellent performance and identifies a large proportion of patients (45-53%) as low risk (DECAF 0-1) and therefore candidates for HAH.[2, 3] This is of importance as ECOPD account for over one million "bed days" in UK hospitals each year and are associated with substantial morbidity and cost.[4-6]

In ECOPD, key differences of HAH to early supported discharge (ESD) are the inclusion of patients with higher medical dependency, replacing all or most of the inpatient stay, and the provision of 24/7 support. The National Institute for Health and Care Excellence (NICE) endorse both services in patients with a low risk of death.[7] In an era of spiralling healthcare demands and costs, providers are open to new models of care.[8] Qualitative studies aid implementation of novel clinical services and add validity to the results of RCTs.[9, 10] Within our RCT, we undertook an embedded qualitative study to identify positive drivers and potential barriers to HAH for ECOPD selected by DECAF and assist wider implementation.

Methods

Participants

Participants included patients within the HAH and usual care (UC) arms of the RCT, their carers, patients who declined enrolment in the RCT, clinicians and hospital managers. HAH and UC patients were purposively selected (CE, LD) in relation to gender, age, socio-economic background, COPD severity (i.e. breathlessness by the extended, modified research council dyspnoea score; forced expiratory flow volume in one second; and previous admissions) and reasons for their preferred place of care. All respiratory specialist nurses (RSN) and consultants, and key acute physicians and managers were invited. Ethical approval was granted by NRES Committee North East-Sunderland (Ref: 13/NE/0275) and the trial was registered (ISRCTN: 290082260). Participants received a telephone call to confirm participation in interviews, informed consent was obtained for the interviews, and all participants were anonymised/ given pseudonyms.

Semi-structured interviews were carried out with 89 participants, which included 44 patients, 15 carers, 15 physicians, 11 RSNs and 4 managers (Hospital: General Manager, operational service manager, and finance. Community: Adult Social Care manager). Eight patients and carers were interviewed together at their request; all other interviews were one-to-one. One study participant was interviewed in hospital, all other participants and carers were interviewed in their home. Participant characteristics are shown in Table 1. The term "decliners" refers to patients who declined participation in the RCT, but consented to interview within this study. Most decliners were interviewed in hospital. Interviews were conducted until data saturation was reached.

For peer review only - http://bmjopen.bmj.com/site/about/guidelines.xhtml

Patients participating in the RCT who had a carer (unpaid individual who helped with daily needs) were identified. Carers were asked to complete the Zarit Burden Interview at baseline, 14 days and 90 days. The Zarit Burden Interview is a multidimensional scale that measures caregiver burden. We planned to present the median values and data range by group, without inferential statistics.

Interviews

Prior to the RCT, the interview schedules were developed within a pilot HAH service and further informed by feedback from inpatients recovering from ECOPD. The final semi-structured interview schedule (online supplement), contained a series of open-ended questions, such as, *"If in the future HAH is routinely available, would you choose to have treatment at home or in the hospital and why?"*

The interviews were designed to explore patients' experience of receiving HAH and UC, preference and perceived influencing factors. The role of the carer within both structures of care and carer-burden were assessed. Clinical and organisational factors that impact on professional's experiences were explored. Patient and carer interviews were arranged post discharge, and all interviews were audio-recorded and transcribed verbatim.

In the subgroup of decliners, interviews were shorter and more focussed on issues related to participation with HAH.

Participants experiences of HAH and UC were explored, with an aim to uncover the drivers and barriers towards the new care pathway, therefore the transcripts were analysed using a combined inductive-deductive method known as "Thematic-Construct Analysis".[11] Data were read for communalities working from a critical perspective.

Researchers remained as faithful as possible to the participants' own accounts using an inductive approach, whilst looking deductively for discourses that were in line with positive drivers and potential barriers of the new care pathway. Each interview transcript was read and re-read by three authors (LD, AvW, SB) to ensure familiarisation with the data; the authors independently coded the data and identified themes. The interpretations were discussed, and the final themes and subthemes were agreed upon (see Table 2).

Member-checking was performed with physicians due to non-familiarity of the new care model. All physicians were sent their own transcripts and asked whether they still agreed with their responses to which three agreed to be interviewed again. Member checking was not performed in other groups because interviews with patients and nurses were longer, they were all familiar with the care provided, numbers were larger, and we did not want to overburden individuals.

Decliners were interviewed (with consent) to elicit their reasons for declining enrolment in the RCT and whether they would consider HAH in the future, if shown to be safe and effective.

Clinical structure of care

Hospital at Home

HAH provides care for patients within their home for a condition that would otherwise require inpatient care.[12] HAH involved treating patients at home supported by a nurse-led respiratory specialist service, after a brief inpatient assessment. Patients were not regarded as sufficiently well

For peer review only - http://bmjopen.bmj.com/site/about/guidelines.xhtml

for discharge. Further information on the service is available including structured clinical assessment sheets and the HAH manual within the online supplement of the RCT.[1]

Usual Care

Patients were managed in accordance with usual hospital care. We engaged with clinical staff, emphasising that clinical decisions should not be influenced by trial participation.

Patient and public involvement

The semi-structured research interviews, intervention (HAH), and outcome measures were informed by interviews with patients and family members, including carers. Most were happy with treatment within HAH provided this was safe. Key concerns with HAH were: clinical deterioration at home, delayed treatment, and lack of social support/ increased carer burden. Direct telephone contact, 24/7, to the respiratory team and the availability of same day social support addressed these issues. Furthermore, we used a validated tool to assess carer burden. An expert patient and non-expert patients informed the selection of health related questionnaires and measures of service acceptability. Patients with AECOPD assessed different home-monitoring devices for acceptability and comfort and reviewed the written description of HAH for clarity which informed the patient information sheet.

Results

Quotes which best presented the established constructs were identified by capital letters using scripts from patients (P), decliners (D), carers (C), consultants (Co), RSN (N) and managers (M). The Respiratory Specialist Registrar's comments were coded Co for anonymity. Quotations have been shortened; both the full quotation and additional supporting quotations are provided in the outline supplement. All patients that were interviewed survived to the end of the study.

Table 1: Description of participants

Participant role	n	Description
Patients participating	31	16 from UC, 15 from HAH. Mean (SD) age= 68 (10) years; 52% female; median (IQR) eMRCD score= 4 (3-4); mean (SD) FEV1= 43.4 (18.7) per cent predicted and previous admissions for an exacerbation= 0 (0-2).
Decliners	13	Mean (SD) age= 73 (11) years; 77% female; median (IQR) eMRCD score= 4 (4-4).
Carers	15	Mean (SD) age= 61 (11) years, 53% female, 80% were the patient's partner. 5 carers' relatives received UC and 10 carers' relatives received HAH.
RSNs	11	Mean (SD) age= 39 (11) years, 100% female, mean (SD) years' experience= 7 (5) years.
Physicians	15	11 respiratory consultants, 3 acute care physicians, and 1 specialist registrar; mean (SD) age= 41.5 (6) years, 71.4% were male, mean (SD) years reported experience = 12 (6)n years.

For peer review only - http://bmjopen.bmj.com/site/about/guidelines.xhtml

Managers	4	3 secondary and 1 social care; mean (SD) age= 50 (2) years, 50% were female.

RSNs, Respiratory specialist nurses; eMRCD, breathlessness score; UC, usual care; HAH, hospital at home; FEV1, forced expiratory volume.

Table 2: Social constructions of Hospital at Home

1. Positive Drivers for HAH
    - 1.1 Availability of home comforts and maintaining independence
        - 1.1.1 Perception of a quicker recovery with HAH and positive effects on perceived breathlessness
        - 1.1.2 Improved sleep and nutrition
        - 1.1.3 More convenient place to be with friends and family
    - 1.2 Confidence in the continuity of HAH care
        - 1.2.1 Feelings of safety, reassurance and appreciation
        - 1.2.2 Personalised relation and specialist expertise of the RSN

2. Potential Barriers and Negative Influences for HAH Care Pathway
    - 2.1 Fear of being alone at home
    - 2.2 Privacy issues and not wanting visitors
    - 2.3 Resistance to change
        - 2.3.1 Reluctance to accept removal of nebulised therapy
        - 2.3.2 Challenging clinician's preconceptions, accepting a new model of care and operational concerns
    - 2.4 Negative influences of HAH

3. Other Insights
    - 3.1 Unintentional change in UC
    - 3.2 Early uncertainty with HAH selected by DECAF

HAH, hospital at home; RSN, respiratory specialist nurse; UC, usual case; DECAF, prognostic score.

1. Positive Drivers for Hospital at Home

1.1 Availability of home comforts and maintaining independence

Patients, carers and RSNs, consistently highlighted that patients were comfortable in their home, with positive influences on mood and confidence. Hospital routines were avoided, and patients maintained their independence by engagement in usual activities. Patients requiring oxygen noted that the equipment provided within HAH allowed free movement, whilst provision in hospital limited mobility. *"I couldn't even walk up the ward… I only had this like little lead… I can go upstairs [at home]… it's nice and I can get up and make myself a meal" (P22L145-153).*

For peer review only - http://bmjopen.bmj.com/site/about/guidelines.xhtml

1.1.1 Perception of a quicker recovery with HAH and positive effects on perceived breathlessness

Patients and carers perceived that recovery occurred more quickly during HAH, and breathlessness was less marked, despite higher activity levels. This may reflect lower levels of anxiety within HAH: *"Just so relaxed… content with the care… because when you're anxious obviously it reacts on your breathing and your whole persona" (P6L109-112).* Compared to UC, delays in obtaining reliever therapy were also avoided within HAH.

1.1.2 Improved sleep and nutrition

Patients reported sleep disruption in UC related to disturbances from other patients, nurses obtaining regular observations and new admissions, whilst those who experienced HAH reported improved sleep: *"There's no bed like your own bed" (N19L129).*

Opinions were diverse concerning nutrition although most favoured HAH. *"(I) liked hospital food and ate more in hospital than at home" (P10L97)* and, *"You don't eat the same in hospital for a start" (P1L18).*

1.1.3 More convenient place to be with friends and family

Home is more convenient and saves time and money for family and friends. Travel, car parking, work absences, childcare issues, restrictive visiting hours and hospital TV costs are avoided. *"It's easier for my family to visit… getting cars parked… when they have been to work all day by the time they get the hospital it's time to come home" (P22L21-24).*

A key benefit of HAH was increased time with grandchildren: "*When I'm in hospital I don't see the grandchildren. I've got six… and three of them live next door and they're here every single day. When I'm not here they do cry; they get really upset" (P13L167-169).*

Carers reported that providing care at home was less disruptive, by avoiding hospital visits which take more time and organisation: "*It's better my husband being at home… instead of… going back and forward to the hospitals and especially with being a carer looking after two children" (P17 L537-548).*

1.2 Confidence in the continuity of HAH care

1.2.1 Feelings of safety, reassurance and appreciation

Patients felt safe and reassured during HAH due to daily visits from the RSNs, the 24-hour telephone support line, confidence in the HAH clinical team, and the availability of emergency services if return to hospital was needed. The evening phone call (9pm) was reassuring to those patients living alone: *"I had confidence in the team that came out... they all seemed to me to be very well trained and putt me at ease" (P1L71-74),* and, *"If they thought you needed a doctor or the hospital they would phone an ambulance straight away" (P12L320-327).*

Most patients were not unduly concerned about potential delays in being seen by a doctor/clinician in the event of deterioration. *"If your hospital at home, if you take really poorly yes you phone up… they will still be here as quick as they would if you were in a hospital" (P12L319-327).*

For peer review only - http://bmjopen.bmj.com/site/about/guidelines.xhtml

Within HAH, patients appreciated the ability to take their medication at their usual time, with immediate access to reliever therapy.

1.2.2 Personalised relation and specialist expertise of the RSN

Patients in HAH appreciated the better continuity in healthcare professionals compared to inpatient care and described the relationship with RSN as “personal”, “individual” and “one to one care”. Clinical assessments conducted at home provided privacy and were less rushed with “time to develop rapport”. Patients valued the clear explanations provided by RSNs during HAH about all aspects of their care, including monitoring, investigations, and management, commenting that this may be lacking in hospital.

Some patients experienced a lack of dignity and privacy during UC. *“A wash down behind the curtain, I was frightened I was rushing” (P32L53-55).* UC patients acknowledged time constraints as a barrier towards personalised care, which was also recognised by the RSNs.

Discharge from UC incurs delays due to waiting for medication, discharge letter and transport. Such concerns were not raised about transfer home under HAH or subsequent discharge.

2. Potential Barriers and Negative Influences for HAH Care Pathway

2.1 Fear of being alone at home

A common reason for declining participation in the RCT was fear of being alone when unwell, even when offered (same day) social support. The RSN stated that some patients were reluctant to accept social services because of perceived stigmatisation. “*The hours I would be on my own… say I got up to go the toilet and my legs went what do you do then if you can’t breathe… you’ve got no phone at hand” (D1, P2L39-41)* and, *“They think if they’ve got a social worker or are involved with social services it’s like they are labelled... they wouldn’t realise they could just ring social services and ask for help” (N4L102-104).*

Participants recognised there may be a longer delay getting help if they deteriorated at home compared to in-hospital care. *“Some people maybe prefer to be in a cocoon of a hospital environment; they maybe worry if something goes wrong someone’s seconds away from them” (P1L226-229).*

2.2 Privacy issues and not wanting visitors

Other decliners referred to privacy issues. “*I thought people coming to my house, I cannot… I’m frightened that it wouldn’t be tidy*” (D3L4-7). For some, personal circumstances affected participation: *“My husband and I are divorcing and my house just having been sold the atmosphere at home wouldn’t be conducive in recuperating… but in the future... I would very much like to be involved” (D9L14-21).*

2.3 Resistance to change

2.3.1 Reluctance to accept removal of nebulised therapy

During HAH patients received similar treatment to those within UC, including controlled oxygen therapy, nebulised bronchodilators and intravenous medications if required. Patients reported that

For peer review only - http://bmjopen.bmj.com/site/about/guidelines.xhtml

nebulised bronchodilators helped chest clearance as well as relieving breathlessness. Patients in HAH were more likely to object to the removal of nebulised therapy than controlled oxygen. Similar objections were not raised to the same extent in UC. The challenge for the RSN was to reassure patients that such interventions were usually not required long term. *"I think a lot of them have the misconception that if they have a nebuliser they are better… Your inhalers are far better more practical" (N3L222-229).*

2.3.2 Challenging clinicians' preconceptions, accepting a new model of care and operational concerns

Physicians' preconceptions included the view that hospital care provides respite for carers and ensures patients feel safer. *"The patient themselves may not be ready for it… I think they need to stay in hospital where they feel safer not because it is safer but basically they feel safer and also it gives a break (to) their carer" (Co2 L53-66).* Another physician disagreed, stating: *"That's kind of often how people view patients with COPD that they want to come to hospital but actually it's not true" (Co3 L198-201).*

Operational concerns included keeping medical records in a patient's home and the inability to capture activity within current payment systems. *"Our work in trying to get the centre to change the way… we can record the data for hospital at home patients… perhaps by us doing that it would make it easier for other trusts to go down the hospital home route in the future" (M1L149-153)* and, *"If we are employing consultants, junior doctors, respiratory nurses, specialists nurses... and we are not having the patient in hospital so we are not getting the tariff for that… somebody has to pay the wage bill… and it's about understanding that payment mechanism… then that becomes a lot easier for everybody because that is the kind of thing that will stop people moving forward" (M17L183-193).*

A different model of care requires adjustment. The RSNs advised that their confidence increased with experience of delivering HAH, whilst patients mentioned that the quality of the service was dependent on the skill of the attending RSN. This highlights the importance of adequate training and support. *"The only concern I would have is that hospital at home is only as good as the nurses you've got on" (P12L336-337).*

2.4 Negative Influences of HAH

Patients felt less inclined to smoke in hospital. *"When I'm in hospital… I don't think about smoking whatsoever where… at home I do"" (P14L273-276).*

The hospital ward is an opportunity to establish new friendships that may continue post discharge and patients were concerned that their relatives might be frightened seeing/ knowing that they are unwell at home. *"I've made some nice friends… still ring them.. It was nice" (P22L497-505). "It's frightening for them to see when you're not well it's not nice for my husband to sit and watch us when I'm bad" (P25L220-222).*

For peer review only - http://bmjopen.bmj.com/site/about/guidelines.xhtml

3. Other Insights

3.1 Unintentional change in UC

Despite efforts to ensure UC was not influenced by trial participation, patients were discharged home earlier than expected. Factors include physician awareness of low risk of death by DECAF, pressure from patients who were disappointed with allocation to UC and pressure from operational service managers. *"There's much more emphasis on getting people home early… the people who we aren't (treating) in the hospital at home I think people are now more likely to send them home a lot earlier" (Co3 L120-126).*

3.2. Early uncertainty with HAH selected by DECAF

Initially some physicians expressed concern that, in their judgement, true risk for an individual patient was underestimated by DECAF. This led to delays in return home, which on occasion was unsettling for patients: *"They have certain reservations, so you've got that conflict between their clinical impression and the predictive score…the simple score almost always outperforms clinical judgement, but clinicians don't like to think that's true" (Co18L40-52).* With experience physicians became more confident using DECAF and the safety within HAH model of care: *"As you get evidence suggesting it works and it's safe you're more confident in doing it" (Co6L154-155).* This was reflected in the wider HAH population as delayed return home was primarily an issue at the start of the trial.

"I think part of the main advantage from my point of view is making me more aware of the safety of the DECAF score and the ability that people can be treated out of the hospital… whether that's because of the hospital at home trial or… because of the gradual change in practice because of the DECAF score I'm not sure" (Co6L99-103).

Carers

In the RCT, 23 carers in HAH and 18 carers in UC completed Zarit Burden Interviews, including 13 of 15 carers participating in this embedded qualitative study. Higher scores show higher levels of care burden. Baseline scores were similar in UC and HAH, but at 14 and 90 days scores were lower in HAH (table 3).

Table 3: Zarit Burden scores in carers

		Count, n	Median (IQR)	% improved or same v baseline	Missing
Zarit Burden 0 day	UC	18	18 (10-29)	Ref	1
	HAH	23	16 (10-30)	Ref	0
Zarit Burden 14 day	UC	18	23 (16-32)	35.7	3
	HAH	23	13 (8-31)	57.9	4
Zarit Burden 90 day	UC	18	25 (13-35)	16.7	5
	HAH	23	14.5 (10-36)	27.8	5

HAH, hospital at home; UC, usual care; IQR, interquartile range

The percentage of patients with no change or an improvement in carer burden scores was higher in HAH, although this could be a chance finding.

For peer review only - http://bmjopen.bmj.com/site/about/guidelines.xhtml

For peer review only

Discussion

We interviewed 89 participants including patients, carers, and healthcare professionals to inform implementation of our HAH model. Whilst our RCT provides the justification for the implementation of HAH regarding cost and clinical outcome, this qualitative study identifies the human and organisational factors that will influence its successful implementation. Positive drivers included: greater independence and freedom; the maintenance of usual activities and perceived shorter recovery time; the maintenance of contact with friends and family (especially grandchildren); better sleep; and more time spent with an expert healthcare professional. Reported barriers from patients included being alone at night and having "strangers" visiting the home. Of note, some of these barriers, such as those relating to privacy and not wanting visitors, may be less of an issue at institutions which already have early supported discharge.

Early concerns from some physicians and RSNs diminished with experience of successful delivery of this model of care. Two patients that were allocated to HAH were kept in hospital at the consultant's discretion; this occurred in the first quarter of the study. Managers highlighted the inability for HAH activity to be captured within current payment systems. Hospitals planning to implement HAH selected by DECAF should pre-emptively address these issues. Compared to ESD, patients within HAH have higher clinical needs. Respiratory and Emergency Care Physicians require education, particularly highlighting the reliability of the DECAF score. RSNs delivering care at home require additional clinical training, consultant support and capacity to deliver the service. The HAH Manual and assessment sheets used within our service are available to facilitate this process. Of note, training costs were included in the health economic analysis, which showed that this model of care is cost-effective. Maintaining clinical documents in a patient's home should be supported by local agreement and patient consent.

The study has several key strengths. We used a qualitative approach with semi-structured interviews to explore the issues around HAH for those involved in the service.[13] We performed a large number of interviews using purposeful sampling of patients to ensure both a wide range of participants and perspectives.[14] Not only were the experiences of patients and carers explored, but operational concerns from managers and clinicians were identified. Of note, we interviewed patients who declined involvement in the trial: this is a group rarely included in such studies. The study sites had no HAH service prior to the RCT, so the emergent themes relate to HAH as a new service. This supports the transferability of our results to other institutions which lack this service. Finally, the views of carers were triangulated with the Zarit Burden Interview.

The study has several limitations. As with all qualitative research, it is impossible to fully remove observer bias. To minimise this risk, interviews were performed by a female trained health psychologist who was separate from the clinical team and had no previous experience or knowledge in the design of the HAH care pathway, and three individuals independently performed analyses of the data to increase trust-worthiness and inter-rated reliability. Certain aspects, such as operational concerns, may only apply to the UK healthcare system. Whilst the inclusion of interviewed personal was large and broad, other members of the multidisciplinary team such as physiotherapists may have provided useful insights. Finally, there is a risk of social desirability bias, in that interviewees may tell us what they think we want to hear. This was minimised by the interviewer emphasising that they were not involved in clinical care and that frank discourse would help develop the service.

For peer review only - http://bmjopen.bmj.com/site/about/guidelines.xhtml

Previous RCTs of domiciliary treatment for patients with ECOPD had more exclusion criteria than our RCT, and mostly were of ESD rather than HAH.[15] Our RCT included patients typically deemed too unwell for home treatment in earlier studies, such as those with pneumonic ECOPD. Perceived risk remains a key factor for engagement, particularly during the early phase of service introduction, which will be important to address during educational sessions. In the DECAF study cohorts, 1,266 of 2,645 patients were DECAF 0-1 (low risk), and risk of death remained low regardless of the index scoring. In the HoT DECAF RCT, there were no acute deaths.[1] This is also in line with patient and carer perceptions in other trials. In a study of elderly patients, which included patients with ECOPD, substitutive HAH was reported as being safe by family and carers.[16] Similar findings were shown in a study of six patients with ECOPD.[17] In our study, patients and carers reported that HAH was safe, noting confidence in the clinical team and the availability of 24/ 7 telephone support, with return to hospital if required.

Five of thirteen patients who declined enrolment in the RCT reported fear of being home alone due to breathlessness, anxiety and/ or problems coping at home. Three patients regretted not enrolling, and three declined partly because of unwell family members in the home. Despite the high rates of pneumonia and co-morbidity, overall healthcare professionals were satisfied that the DECAF score selected low risk patients suitable for HAH. In the whole HAH population (n=60), there were only three instances of discordance between clinician judgement and the DECAF score leading to patients randomised to HAH receiving UC, all of whom survived to 90 days follow up.

Carers of patients with COPD are at risk of various psychological health problems, such as anxiety and depression; partners of patients with COPD are typically elderly with their own health problems.[18] Utens and others looked at carer strain in an RCT of ESD compared to usual care, and showed no difference in the caregiver strain index between allocation groups.[19] Given the higher treatment needs of patients in HAH, we anticipated that the carers of patients treated with HAH would report increased carer burden. However, carers reported that inpatient stay was more disruptive than HAH, both in terms of time and cost. This was supported by the Zarit Burden Interview scores, which showed that the median carer burden scores were numerically lower with HAH than UC, though this could have been a chance finding. Whilst patients may express a fear of being or becoming a burden on their carers at the end of life,[20] this was not reported in any of the interviews with patients in the context of HAH for an acute exacerbation.

HAH selected by DECAF allows the inclusion of more patients than existing models, is preferred to inpatient care by most patients and their families, and is considered to be safe. HAH and the associated care was valued by many patients, in particularly with regards to continuity of care and in maintaining individuals' independence. Following this study, HAH has been commissioned as a service for all patients with low-risk ECOPD. The results of this study, both in terms of potential drivers and barriers, are important areas of discussion when offering HAH to patients.

For peer review only

Acknowledgements

This paper presents independent research funded by the National Institute for Health Research (NIHR) under its Research for Patient Benefit (RfPB) Programme (Grant Reference Number PB-PG-0213-30105). The views expressed are those of the authors and not necessarily those of the NHS, the NIHR or the Department of Health.

We thank Teresa Gibson for her input to the trial management group as an expert patient, the members of the Data Monitoring Committee (Robert Angus, Rodney Hughes, Nick S Hopkinson and Niall Anderson) and the Trial Steering Committee (Nicholas Hart, Jennifer Quint, Jadwiga Wedzicha, Patrick Murphy, Robert Rutherford). We thank Tracey Finch for her input in the initial draft of the project.

We are grateful to all interviewees for their time and candour.

Contributors

Stephen Bourke conceived the study and was chief investigator with overall responsibility for the management of the study. Stephen Bourke, Anna van Wersch and Carlos Echevarria were responsible for the original design and protocol, and obtaining funding. John Gibson contributed to the study design methodology. Lorelle Dismore and Carlos Echevarria recruited patients to the study. Lorelle Dismore performed all interviews and transcripts. Stephen Bourke, Anna van Wersch, and Lorelle Dismore analysed all transcripts for themes. Carlos Echevarria performed statistical analyses. Lorelle Dismore and Anna van Wersch wrote the first draft of the manuscript. John Gibson provided a critical review for important intellectual content. All authors were involved in the final version of the manuscript and approved the manuscript for publication.

Funding

NIHR Research for Patient Benefit and Northumbria Healthcare NHS Foundation Trust Teaching and Research Fellowship programmes.

Competing interests

All authors have completed a ICMJE uniform disclosure. SCB reports grants from NIHR: Research for Patient Benefit programme, during the conduct of the study; HTA funding, grants from Philips Respironics, grants from Pzifer Open Air, personal fees from Pzifer and AztraZeneca outside the submitted work. AvW reports grants from NIHR: Research for Patient Benefit programme, during the conduct of the study. LD, CE and GJG have no competing interests to declare.

Patient consent

Obtained

For peer review only - http://bmjopen.bmj.com/site/about/guidelines.xhtml

Ethics approval

Ethics approval was provided by NRES Committee North East Sunderland (3/NE/0275). All participants gave informed consent before taking part in the study.

Data Sharing Statement

The data are anonymised transcripts and are available upon reasonable request by emailing Lorelle.Dismore@northumbria-healthcare.nhs.uk. However, additional supporting quotes for the identified themes can be found in the supplementary information.

For peer review only

For peer review only - http://bmjopen.bmj.com/site/about/guidelines.xhtml

References

1. Echevarria C, Gray J, Hartley T, et al. Home treatment of COPD exacerbation selected by DECAF score: a non-inferiority, randomised controlled trial and economic evaluation. *Thorax* 2018; 73(8): 713-22.
2. Steer J, Gibson J, Bourke SC. The DECAF Score: predicting hospital mortality in exacerbations of chronic obstructive pulmonary disease. *Thorax* 2012;67(11): 970-6.
3. Echevarria C, Heslop-Marshall K, Stenton SC, et al. Validation of the DECAF score to predict hospital mortality in acute exacerbations of COPD. *Thorax* 2016; 71(2): 133-40.
4. British Thoracic Society. The burden of lung disease: a statistical report from the British Thoracic Society, 2nd ed. London: British Thoracic Society, 2006. https://www.brit-thoracic.org.uk/document-library/delivery-of-respiratory-care/burden-of-lung-disease/burden-of-lung-disease-2006/
5. Seemungal TA, Donaldson GC, Paul EA, Bestall JC, Jeffries DJ, Wedzicha JA. Effect of exacerbation on quality of life in patients with chronic obstructive pulmonary disease. *Am J Respir Crit Care Med* 1998; 157(5): 1418-22.
6. Vestbo J, Hurd SS, Agusti AG, et al. Global strategy for the diagnosis, management, and prevention of chronic obstructive pulmonary disease: GOLD executive summary. *Am J Respir Crit Care Med* 2013; **187**(4): 347-65.
7. National Institute for Health and Care Excellence. *Chronic obstructive pulmonary disease in over 16s: diagnosis and management.* England: NICE; 2010.
8. NHS England, Public Health England, Health Education England, Monitor, Care Quality Commission, NHS Trust Development Authority Five Year Forward View 2014. https://www.england.nhs.uk/wp-content/uploads/2014/10/5yfv-web.pdf
9. Campbell M, Fitzpatrick R, Haines A, et al. Framework for design and evaluation of complex interventions to improve health. *BMJ.* 2000; 321(7262): 694-6.
10. Bradley F, Wiles R, Kinmonth AL, Mant D, Gantley M. Development and evaluation of complex interventions in health services research: case study of the Southampton heart integrated care project (SHIP). *BMJ.* 1999; 318(7185): 711-5.
11. Dismore L, van Wersch, A, Swainston K. Social constructions of the male contraception pill: When are we going to break the vicious circle? *J Health Psychol* 2016; **21**(5): 788-97.
12. Shepperd S, Lliffe S. Hospital at home versus in-patient hospital care. *Cochrane Database Syst Rev.* 2005(3). Issue 2. CD000356
13. Greenhalgh T, Taylor R. How to read a paper: papers that go beyond numbers (qualitative research). *BMJ* 1997; 315(7110): 740-3.
14. Bryman A. Social Research Methods: Oxford University Press, 2008.
15. Echevarria C, Brewin K, Horobin H, et al. Early Supported Discharge/ Hospital At Home For Acute Exacerbation Of Chronic Obstructive Pulmonary Disease: A Review and Meta-Analysis. *COPD Journal.* 2016; 13(4): 523-33.
16. Leff B, Burton L, Mader SL, et al. Comparison of stress experienced by family members of patients treated in hospital at home with that of those receiving traditional acute hospital care. *J Am Geriatr Soc* 2008; 56(1): 117-23.
17. Wang Y, Torbjorn H, Steihaug S. & Werner A. Patients with acute exacerbation of chronic obstructive pulmonary disease feel safe when treated at home: a qualitative study. *BMC Pulm Med* 2012; 12(1): 45.
18. Gardiner C, Gott M, & Small N. Living with advanced chronic obstructive pulmonary disease: patients concerns regarding death and dying. *Palliat Med* 2009; 23: 691-7.
19. Utens CM, van Schayck OC, Goossens LM, et al. Informal caregiver strain, preference and satisfaction in hospital-at-home and usual hospital care for COPD exacerbations: results of a randomised controlled trial. *Int J Nurs Stud* 2014; 51(8): 1093-102.

For peer review only - http://bmjopen.bmj.com/site/about/guidelines.xhtml

20. Jones I, Kirby A, Ormiston P, et al. The needs of patients dying of chronic obstructive pulmonary disease in the community. *Fam Pract* 2004; 21(3): 310-3.

For peer review only

For peer review only - http://bmjopen.bmj.com/site/about/guidelines.xhtml

Table of themes with quotations

Online supplementary report of full and additional quotations related to themes. Interviewer quotes are shown in bold.

Theme 1. Positive Drivers for HAH	Quotes
1.1 Availability of home comforts and maintaining independence	I couldn't even walk up the ward or anything you know because I only had this like little lead you know and I used to keep forgotten walking off then if you wanted to talk to anybody you had to either shout or they had to come to you, you know but that's the only thing just sort of sat there all the time (P22L145-148)<br><br>**And now in the home how would you describe this situation with the oxygen lead?**<br><br>Oh yes I can go upstairs do different pieces up there you know tidy my draws up and that's you know just a couple at a time just err no its nice and I can get up and make myself a meal you know (P22L145-153)<br><br>Yeah well what can you do in hospital you're either lying on a bed or sitting in a chair I can walk back and forward in here you know the and I can go upstairs and down bath wash shave that type of thing you know were as well you can do that in hospital get washed and shaved and that but you're stuck in the one place you're not going anywhere you know you're not getting no exercise nothing (P18L114-118)<br><br>In a lot of ways you're disempowering the patient by keeping them on a ward and keeping them within that sort of very tight sort of role, when you get them home then they're, they're dressed, they are doing more things themselves (N7L293-296)
1.1.1 Perception of a quicker recovery during HAH and positive effects on perceived breathlessness	Her recovery time was much quicker so it was easier for me because she was helping herself do you know what I mean (CP28L260-261)<br><br>Just so relaxed content, content with the care everything just made everything because when your anxious obviously it reacts on your breathing and your whole persona really and just everything was so relaxed I don't know how else to describe it (P6L109-112)
1.1.2 Improved sleep and nutrition	People recover better in their own home and there was one lady and I quote her often to the staff there's no bed like your own bed and that stuck because actually she was right absolutely right you know why be sitting in

For peer review only

	a hospital bed when you've got poorly people around you if you have that support to be able to recuperate at home (M19L128-132).<br><br>I was very surprised at the food it wasn't too bad. In fact I had more in the hospital than I did at home (P10L92-97)<br><br>You don't eat the same in hospital for a start and you certainly don't sleep the same you can be awake most of the night (P6L91-92)
1.1.3 More convenient place to be with friends and family	It's easier for my family to visit and everything you know getting cars parked you know when they have been to work all day by the time they get the hospital it's time to come home you know so it's definitely benefitted me you know (P22 L21-24)<br><br>But even the grand children when I'm in hospital I don't see the grandchildren I've got six grandchildren and three of them live next door and they're here every single day when I'm not here they do cry they get really upset (P13 L167-169)<br><br>They don't like fetching them in when they're so young I mean the youngest ones four and the twins and they miss us when I'm not here (P16L87-88)<br><br>It's better my husband being at home and getting the treatment at home instead of like going back and forward to the hospitals and especially with being a carer looking after two children as well you know it's a lot better<br><br>**So when you say you look after two children as well, so are you a carer for those two children and Jim?**<br><br>Yes<br><br>**So hospital at home how does that make it easier in regards to that?**<br><br>Like with looking after Jim and getting the support and like for visit and things you know like when he's at home he's at home and getting looked after at home and I can think of the children and it doesn't put as much

For peer review only

	pressure on us as well because like when you're visiting and you've got children to pick up at school as well you know it's all like the time as well (P23 L537-548)<br><br>It's been much better for me anyway because I used to get knackered go into hospital every day and coming back then having to go back again and coming back whereas been here I can go to work come in do whatever I wanted to do when I knew she was safe she was all right in the house do you know what I mean (P28L225-228)<br><br>Financial yes, yes because you see were on a low income my partner only works part time window cleaner and we have to pay full rent and everything and money is really tight and for him to keep coming back and forth to hospital I mean sometime if there's no money he can't go you know so that's the benefits because he even though he's a part time window cleaner he can still go to work when I'm poorly he works local (P13L56-61)<br><br>At first I was a bit concerned because I look after the grandchildren not every day four days a week sometimes erm and I was more concerned about them whether it would interfere with personal life sort of thing err and then went and thought about it, it obviously wouldn't I'd get to see them more than what I would actually been in hospital so that was the deciding factor really (P6L12-16)
1.2 Confidence in the continuity of HAH care<br>1.2.1 Feelings of safety, reassurance and appreciation	I had confidence in the team that came out I think I saw four different people over a short period of time they all seemed to me to be very well trained and putting me at ease and I really didn't feel like I was missing anything that I would have had in hospital you know (P1L70-73)<br><br>Now I feel what's the word more secure I don't know yeah more secure because I know all I've got to do is make a phone call like obviously there's name of doctor there's obviously the nurses if I have to there would be you there is more people to talk to now and that's something I never had before which is good' (P13L103-106)<br><br>Few have said but are you not nervous X like if you're in a hospital you're on a ward they get a doctor to you as soon as they can and they might not get it as soon as you can might be two three hours down the line but if your hospital at home if you take really poorly yes you phone up you've got that number they will still be here as quick as they would if you were in a hospital do you know what I mean it doesn't mean to say because you're on a ward that a nurse or a doctors going to get to you do you know and I would phone a the district nurse or community nurse and if they thought you needed a doctor or the hospital they would phone an ambulance straight away (P12L321-328)

For peer review only

	Telephone call before I came out I got a telephone call before the afternoon in the evening check that things were okay I was told on that evening who would be coming the next day so I knew who to expect so I don't have a problem at all with it (P1 P7 L320-323)
1.2.2 Personalised relation and specialist expertise of the RSN	A wash down behind the curtain I was frightening I was rushing (P32L53-55)<br><br>I mean you cannot get individual care can you really because there's 16 on the ward another five people in care and of course at home you're getting the care more attention (P16 L163-165)<br><br>They checked everything every day you know everything ankles everything whereas a rule it's just your blood pressure and your oxygen level in the hospital and your erm temperature yes but I don't know they were just really brilliant they were here quite a long time you know making sure I was feeling all right and I was confident on my own you know but no I found them really good and there are so nice you know really nice and caring (P22L44-49)<br><br>I'll be perfectly honest the times I've been in the hospital the nurses there been too busy or there's only been one on or two on and they can't do anything you know there to busy doing other things you don't get a one to one erm but in your home you do get one to one (P12L208-211)<br><br>She made me feel safe she made me feel I was getting a proper treatment erm comfortable is the word she made me feel really comfortable knew what she was doing explained everything even down to when you were getting your stats done you know when you were getting while you were getting your bloods taken erm explained everything you don't always get that in a hospital (P12L365-370)<br><br>You get told you can get out tomorrow and then they say you've got tablets and everything and by the time its tea time you're still waiting there somebody coming to get you or getting the tablets or getting the letter but everything's so well done there was didn't get you harassed at all and it just seemed like you go home today you've got your tablets you've got your letter you were taken down to the departure place the nurse was there they had been with the oxygen so well planned it was like err just sail a long and I like that because it didn't get as well erm and it made you feel more relaxed and thinking to yourself well if this is how it's going to be then that's okay I can cope (P20L423-431)
2. Potential Barriers and	

For peer review only

Negative Influences of HAH Care Pathway	
2.1 Fear of being alone at home	The hours I would be on my own you know if just say I got up to go the toilet and my legs went what do you do then if you can't breathe you know you've got no phone at hand you know you cannot pull an alarm switch or anything so no (Decliner 1 P2L39-41)<br><br>Maybe it's the generation thing I mean mention a social working oh no I haven't got one of them but I mean you know I think they think if there've got a social worker or they're involved with social services it's like there labelled but it's not that but they don't realise that so you know until its explained to them so there would be sat at home and they wouldn't realise they could just ring social services and ask for help (N4L100-104)<br><br>Some people maybe prefer to be in a cocoon of a hospital environment, they maybe worry if something goes wrong someone's seconds away from them (P1L226-229)
2.2 Privacy issues and not wanting visitors	I thought people coming to my house, I cannot I'm frightened that it wouldn't be tidy and things like that, that's what type of person I am I'm frightened that it wouldn't be tidy (D3L4-7)<br><br>My husband and I are divorcing and my house just having been sold the atmosphere at home wouldn't be conducive in recuperating…but in the future ... I would very much like to be involved (D9L14-21).
2.3 Resistance to change<br>2.3.1 Reluctance of the removal of nebulised therapy	The nebulisers seem to clear you and they clear you quickly and they seem as if erm you get relief after you've had them (P12L90-91)<br><br>I mean I take all my self-medication at home anyway but you know I'm on steroids and antibiotics and so I've got them at home anyway as I say it's just if I had a nebuliser at home that would sort of cure everything I think (P7L57-59)<br><br>I would still rather have home treatment you know if I had a nebuliser I would be happy as Larry I would be because once I'm on that and take a couple of doses I'm fine (P7L69-71)<br><br>I feel safer actually with that there (P27L311-313)

For peer review only

	I think a lot of them have the misconception that if they have a nebuliser they're better and that seems to stick in their mind so of course then we have to go down do you realise it increased your heart rate its higher mortality rate ideally it's for emergency you shouldn't be having them all the time so we do sort of stress to them you don't want to be on this treatment your inhalers are far better more practical more easier to use so we do try our best to get them off (N3L223-229)
2.3.2 Challenging clinicians' preconceptions, accepting a new model of care and operational concerns	The patient themselves may not be ready for it I may be ready for it but the patient may be two days away from it and also sometimes the patient needs would like if someone came into a hotel for their illness for a while they want to be away from their sick bed because their home is almost like a toxic area so all I'm doing is what I'm trying to tell the patient is I can actually bring the hospital to your home with some patients it's a good idea but some patients say actually I want to take a break from my home<br><br>**So is that the major difference in those patients who want to stay in hospital?**<br><br>Yeah because I think they need to stay in hospital where they feel safer not because it is safer but basically they feel safer and also it gives a break their carers would like to get rid of them for two or three days there's many different things going on in the patient the mind of the patient<br>(Co2 P2L53-66)<br><br>That's kind of often how people view patients with COPD that they want to come to hospital but actually it's not true (Co3 L198-201)<br><br>Our work in trying to get the centre to change the way erm we can record the data for hospital at home patients erm perhaps by us doing that it would make it easier for other trusts to go down the hospital home route in the future (M1P4 L151-153)<br><br>If we're employing consultants junior doctors respiratory nurses specialists nurses and health cares we're not having the patient in hospital so we're not getting the tariff for that but we're sending those patients out to look after them somebody has to pay the wage bill for that and it's about understanding that payment mechanism and if that's absolutely crystal clear then that becomes a lot easier for everybody because that's the kind of thing that will stop people moving forward (M17L188-193)

For peer review only

	Think the only concern I would have is that hospital at home is only as good as the nurses you've got on (P12L336-337)
2.4 Negative Influences of HAH	I mean for all I'm a smoker when I'm in hospital, things like that, I don't think about smoking whatsoever where I am at home I do but erm whenever I'm in a strange place like that I never smoking never crosses my mind (P14L273-276).<br><br>I've made some nice friends when I was in for a week you know still ring them you know erm but that was nice but I didn't wander round and talk to them you know but erm as I say erm the first time I went in it was nice (P22L497-499)<br><br>It's frightening for them to see when you're not well it's not nice for my husband to sit and watch us when I'm bad (P25L220-222)
3. Other Insights<br>3.1 Unintentional change in UC	There's a much more emphasis on getting people home early and I think even the people who are in the usual care arm so the people who we aren't doing in the hospital at home I think people are now more likely to send them home a lot earlier than they did when I did my stuff four years ago so I think general practice has changed we're not hanging on to these patients as long as we did erm and that's through external pressures erm but you know external pressures make you want to get people home as quickly as possible but the trial itself might be running the trial might also be changing peoples practice and may be making them send people home earlier (Co3 L121-129)
3.2 Early uncertainty with HAH selected by DECAF	They have certain reservations so you've got that conflict between there clinical impression and the predictive score. There are patients where as I said their risk is low but the clinical judgement is that they are not low risk now the difficulty here is that we shouldn't necessarily ignore our clinician judgement because well it's important and it's important to listen to however again and again the studies show that clinical judgement is not as good as prognostic scores so you've got research from the 1950s and literally hundreds of studies now from medicine and kind of baseball wine-making you name it completely different types of erm different types of expertise the simple score almost always out performs clinical judgement but clinicians don't like to think that's true (Co18L40-52).<br><br>For some cases it seems obviously a good thing to do then there's other cases where previously you might have

For peer review only

	thought better keep those inpatients in for a few days rather than sending them home so a couple of patients were that's realise that's different from what you normally do and you do think not anxiety but you do think oh I hope that things are going to alright but that's why we're doing a trial because we don't know (Co3L22-27)<br><br>As you see it works and as you get evidence suggesting it works and its safe you're more confident in doing it (Co6L154-155)<br><br>I think part of the main advantage from my point of view is making me more aware of the safety of the DECAF score and the ability that people can be treated out of the hospital so more whether that's because of the hospital at home trial or whether that's just because of the gradual change in practice because of the DECAF score I'm not sure (Co6L99-103)

For peer review only

Interview questions – Carers

Background to the carer

1) Can you describe your relationship to (X)?
2) Can you describe the care you give at home?
3) What sort of care do you provide?
4) Can you describe a typical day/how much of your day does it take?
5) How has your life changed since you have been providing care?
    - Has it affected your daily routine (what was it like before/how is it now – how would you describe what you do – how do you feel)?
    - What's good or bad about it?
    - Is it becoming easier/harder – in what ways?
    - What do you find hardest about caring for someone? (loneliness/fear/isolation/financial difficulties)
6) How has care changed over time?
7) Is there a change when your relative's health suddenly worsens?
8) What helps you in providing care?
    - (coping/support/faith/family)
    - Do you receive any help/support from anyone?
10) How do you feel about providing care? (burden/guilty/helpless)
11) What do you think about someone from a social services team coming in to help provide care?
12) What does the word carer mean to you/how do you feel about the word carer?

Project Questions

1) What are your perceptions of hospitals?
2) Has your relative been previously hospitalised?
3) Can you describe past experiences of hospital?
4) How do you feel if your relative has to go into hospital?
5) Can you describe a typical day on which your relative is hospitalised – what type of care do you provide?
6) Can you tell me what your initial thoughts were when you were told about HAH?
7) Initially your reasons for preferring home treatment were (QUOTE). Can you explain this in more detail?
8) How did you find (mode of treatment)?
    - Expectations of (mode of treatment)/different from your experience?
9) How was it being at (mode of treatment) with your relative?
10) How would you have felt if (X) was treated (mode of treatment)?
11) Can you describe what happened whilst (X) was being treated (mode of treatment)?
    - What did they do whilst at home? Relaxed or not?
12) What is your preference now following your recent experience?
13) If in the future HAH is available, would you choose to have your relative treated at home or in hospital? Why?

For peer review only

14) Can you describe the care you give your relative comparing when at home and hospitalised? – any differences?
15) Where would you prefer to care for your relative?
16) How does it benefit you?
17) Any disadvantages of having to care for your relative at (mode of treatment)?
18) What concerns would you have in the future if your relative is treated at (mode of treatment)?

Dr-patient Relationship

1) Can you describe your experience of the interaction between yourself and the doctors/nurses or others you have seen?
2) Can you describe the role of the nurse/doctor?
3) Can you describe the relationship you had with the nurse?
    - What do you talk about with the nurses – what do they say to you?
4) In what ways would the relationship differ between a nurse visiting your relative at home compared to on a ward?
5) Home/respiratory specialist nurse, what do you think about this?
6) Did you understand what was required from you? Concerns?
7) Anything missing/any disadvantages?
8) What would have made it better for you?
9) What went particularly well or what were the main benefits for you?

Nebulisers

1) What do you understand about nebulisers/inhalers?
2) Can you describe a situation in which your relative has needed to use one - in (mode of treatment) - What's the difference in using one at home versus in hospital?
3) Why would your relative use a nebuliser/inhaler, what would trigger its usage, anything in particular?
4) Is there any issue of using one at home? Are you using it more often/over using it/is it better at home?
5) How often do you find they use a nebuliser/inhaler at home?
6) What are the advantages/disadvantages of nebulisers/inhalers?

Summary question

1) Anything else you would like to tell me or anything else you think I should know?

Interview questions – Health care professionals (clinician interviews)

1) Can you begin by telling me what you understand about hospital at home (HAH)?
2) Would you be able to share your views of HAH – What do you feel about home treatment?
3) How did you become involved in the project and how long for/would you like more involvement?
4) Can you describe your role? And what affect if any the project has on it?
5) Have you been involved in the treatment of patients at home/in hospital – can you describe this?

For peer review only

6) What do you think about when considering if a patient is eligible for HAH?
7) Have you had any patients that you haven't put through to the trial/any situation where you would not recommend a patient for HAH?
8) What's the difference in care if any a patient receives in HAH?
9) Organisation wise how do you feel the project is running?
10) What were your expectations of HAH? Have these changed?
11) Is there anything that is going particularly well about it or what are the main benefits you can see?
12) Do you see any disadvantages or can you recommend an area of improvement?
13) Do you have any preferences as to where to treat patients?
14) What do you think about the future implementation?
15) Is there anything else you think I should know or anything you would like to tell me?

Interview schedule for managers

1) Can you begin by telling me what you understand about hospital at home (HAH)?
2) Would you be able to share your views of hospital at home – how do you feel about home treatment as a service for patients?
3) With regard to the current RCT of HAH for patients with an exacerbation of COPD, can you describe your role and your involvement in the trial?
4) From the perspective of this Trust, how do you think the trial is running?
5) What are your views on using DECAF to identify low risk patients?
6) Is there any conflict between clinician judgement and DECAF?
7) What were your expectations of HAH? Have these changed?
8) Can you recommend an area of improvement?
9) What are the advantages from a patients'/clinicians' perspective?
10) What are the disadvantages from a patients'/clinicians' perspective?
11) Is there anything that is going particularly well about it or what are the main benefits you can see?
12) Can you describe the role of a respiratory nurse?
13) Can you describe the interaction between the respiratory nurses and consultants?
14) What are the advantages, and challenges, from the perspective of a hospital manager or director/ hospital information services and management?
15) Have there been any issues with the trial?
16) How do we establish and support hospital at home as a clinical service?
17) What support do you think the clinical staff need to effectively implement the service?
18) Should it be possible, if the trial results are positive and no major issues are experienced, that this could be used as a model to establish and implement similar services in other trusts?
19) Are there any barriers towards implementation? If so, how do you think these should be tackled?
20) What do you think of it being a seven day service?
21) If other trusts are to implement the trial how do we support this?
22) What level of central engagement is needed if the trial is successful to make it easier for other trusts?

For peer review only

23) Anything else you would like to discuss?

Interview Questions for patients

Experience

1) Can you describe your experience of how you came to be in hospital?

Project information

1) Can you tell me your initial thoughts when asked to participate in the study?
2) Initially you stated a preference for (mode of treatment). Has your preference changed following your recent experience?
3) Initially your reasons for preferring (mode of treatment) were (quote) can you explain this in more detail?
4) How did you find (mode of treatment)?
5) If in the future HAH is routinely available, would you choose to have treatment at home or in hospital? Why?
6) What were your expectations previously and was your experience different from these?
7) How did you feel when at (mode of treatment)?
8) How would you have felt if treated (mode of treatment)?
9) How do you feel when the nurse or doctor comes to visit you at (mode of treatment)/would this differ if you were (in other mode of treatment)?
10) If any of your friends had any problems would you suggest to them to go for HAH?
11) Would there be circumstances with your friends where you think, "This would not suit this person but it suits me?"
12) Did you ever have to ring the nurse whilst at home – what was your experience?
13) Can you describe the role of the doctors/nurses or others you have seen?
    - Did you understand what was required from you? Concerns?
    - Anything missing/any disadvantages?
    - What would have made it better for you?
    - What went particularly well or what were the main benefits for you?

Nebulisers

1) Can you describe a situation in which you have needed to use a nebuliser? - Hospital/at home?
2) What's the difference between using one at home versus in hospital?
3) Why would you use one at home/what would trigger its usage, anything in particular?
4) (at home) Are you using it more often/over using it/how do you find using it/how often?
5) Any situation where you would prefer not to use one? Why?
6) What are the benefits/disadvantages?
7) Summarise - Is there anything else you would like to tell me?

Interviews Nurses

1) Can you begin by telling me what you understand about hospital at home (HAH)?
2) Would you be able to share your views of HAH – What do you feel about home treatment?

For peer review only

3) How did you become involved in the project – what's you role?
4) What affect does the project have on your role as a nurse – how has it changed your role?
5) Are you happy with your involvement would you like more/less?

Treatment

1) Have you been involved in the treatment of patients at home– can you describe this?
2) What's it like visiting patients in their homes/how do you feel when there?
3) How does this differ to treating someone in hospital? What's the main difference to you?
4) Have you ever done supported discharge visits?
5) How does HAH compare to this – similarities/differences?
6) Is there any anxiety over one or the other – why?
7) How do you feel making decisions for patient's treatment?
8) Have you had a situation where it has not benefited the patient or can you see were this may happen?

Nebulisers

1) Is there a difference in nebuliser treatment in patients at HAH versus UC?
2) Is there a difference in how patients use them? Why?
3) Is there any reluctance to take the nebuliser away from those at home? Difference in how you use them?
4) How often do patients use them at home? What happens if there not used?
5) Any issues with nebulisers that you can see?
6) Do you have a preference as to why you prefer to treat patients?

The project

1) Organisation wise how do you feel the project is running?
2) How has it changed normal procedures – what's now different about it?
3) Is there anything that is different about it that people may not necessarily agree on?
4) Are there any issues with the HAH patients having their clinical notes?
5) What were your expectations of HAH? Have these changed?
6) Is there anything that is going particularly well about it or what are the main benefits you can see?
7) Do you see any disadvantages or can you recommend an area of improvement?
8) How do you feel over all – pleasure/burden? Concerns?
9) What do you think about the future implementation?
10) What are your views on the on call respiratory physician?
11) How has this impacted?

Relationships

1) Can you describe the relationship between yourself and the patients when in a patient's home?
2) What do you speak to patients about?
3) What do they tell you?
4) Can you describe the relationship with patients when seeing them on the ward?
5) What's the relationship like with patients at home/compared to hospital?
6) Is there a difference in relationships between men and women?

For peer review only

7) Ever feel intimidated or are you ever more careful?
8) What do you feel is important for a good working relationship with patients?

Hospital A

1) What are you views on Hospital A?
2) How has Hospital A impacted the process of HAH? Easier/harder?

Anything else?

For peer review only

COREQ (COnsolidated criteria for REporting Qualitative research) Checklist

A checklist of items that should be included in reports of qualitative research. You must report the page number in your manuscript where you consider each of the items listed in this checklist. If you have not included this information, either revise your manuscript accordingly before submitting or note N/A.

Topic	Item No.	Guide Questions/Description	Reported on Page No.
**Domain 1: Research team and reflexivity**			
Personal characteristics			
Interviewer/facilitator	1	Which author/s conducted the interview or focus group?	10
Credentials	2	What were the researcher's credentials? E.g. PhD, MD	10
Occupation	3	What was their occupation at the time of the study?	10
Gender	4	Was the researcher male or female?	10
Experience and training	5	What experience or training did the researcher have?	10
Relationship with participants			
Relationship established	6	Was a relationship established prior to study commencement?	NO
Participant knowledge of the interviewer	7	What did the participants know about the researcher? e.g. personal goals, reasons for doing the research	
Interviewer characteristics	8	What characteristics were reported about the inter viewer/facilitator? e.g. Bias, assumptions, reasons and interests in the research topic	10
**Domain 2: Study design**			
Theoretical framework			
Methodological orientation and Theory	9	What methodological orientation was stated to underpin the study? e.g. grounded theory, discourse analysis, ethnography, phenomenology, content analysis	4
Participant selection			
Sampling	10	How were participants selected? e.g. purposive, convenience, consecutive, snowball	3
Method of approach	11	How were participants approached? e.g. face-to-face, telephone, mail, email	3
Sample size	12	How many participants were in the study?	3-4
Non-participation	13	How many people refused to participate or dropped out? Reasons?	NONE
Setting			
Setting of data collection	14	Where was the data collected? e.g. home, clinic, workplace	3
Presence of non-participants	15	Was anyone else present besides the participants and researchers?	NO
Description of sample	16	What are the important characteristics of the sample? e.g. demographic data, date	4
Data collection			
Interview guide	17	Were questions, prompts, guides provided by the authors? Was it pilot tested?	3
Repeat interviews	18	Were repeat inter views carried out? If yes, how many?	4
Audio/visual recording	19	Did the research use audio or visual recording to collect the data?	4
Field notes	20	Were field notes made during and/or after the inter view or focus group?	
Duration	21	What was the duration of the inter views or focus group?	30-60
Data saturation	22	Was data saturation discussed?	3
Transcripts returned	23	Were transcripts returned to participants for comment and/or	

For peer review only - http://bmjopen.bmj.com/site/about/guidelines.xhtml

Topic	Item No.	Guide Questions/Description	Reported on Page No.
		correction?	4
**Domain 3: analysis and findings**			
Data analysis			
Number of data coders	24	How many data coders coded the data?	10
Description of the coding tree	25	Did authors provide a description of the coding tree?	
Derivation of themes	26	Were themes identified in advance or derived from the data?	4
Software	27	What software, if applicable, was used to manage the data?	[illegible]
Participant checking	28	Did participants provide feedback on the findings?	
Reporting			
Quotations presented	29	Were participant quotations presented to illustrate the themes/findings? Was each quotation identified? e.g. participant number	5 – 9
Data and findings consistent	30	Was there consistency between the data presented and the findings?	
Clarity of major themes	31	Were major themes clearly presented in the findings?	5 – 9
Clarity of minor themes	32	Is there a description of diverse cases or discussion of minor themes?	6, 7, 8,

Developed from: Tong A, Sainsbury P, Craig J. Consolidated criteria for reporting qualitative research (COREQ): a 32-item checklist for interviews and focus groups. *International Journal for Quality in Health Care*. 2007. Volume 19, Number 6: pp. 349 – 357

Once you have completed this checklist, please save a copy and upload it as part of your submission. DO NOT include this checklist as part of the main manuscript document. It must be uploaded as a separate file.
